# Partial-breast radiotherapy after breast conservation surgery for patients with early breast cancer (UK IMPORT LOW trial): 5-year results from a multicentre, randomised, controlled, phase 3, non-inferiority trial

**DOI:** 10.1016/S0140-6736(17)31145-5

**Published:** 2017-09-09

**Authors:** Charlotte E Coles, Clare L Griffin, Anna M Kirby, Jenny Titley, Rajiv K Agrawal, Abdulla Alhasso, Indrani S Bhattacharya, Adrian M Brunt, Laura Ciurlionis, Charlie Chan, Ellen M Donovan, Marie A Emson, Adrian N Harnett, Joanne S Haviland, Penelope Hopwood, Monica L Jefford, Ronald Kaggwa, Elinor J Sawyer, Isabel Syndikus, Yat M Tsang, Duncan A Wheatley, Maggie Wilcox, John R Yarnold, Judith M Bliss, IMPORT Trialists, Wail Al Sarakbi, Sarah Barber, Gillian Barnett, Peter Bliss, John Dewar, David Eaton, Stephen Ebbs, Ian Ellis, Philip Evans, Emma Harris, Hayley James, Cliona Kirwan, Julie Kirk, Helen Mayles, Anne McIntyre, Judith Mills, Andrew Poynter, Elena Provenzano, Christine Rawlings, Mark Sculpher, Georges Sumo, Mark Sydenham, Andrew Tutt, Nicola Twyman, Karen Venables, Anna Winship, John Winstanley, Gordon Wishart, Alastair Thompson

**Affiliations:** a Department of Oncology, University of Cambridge, Cambridge, UK; b Clinical Trials and Statistics Unit, The Institute of Cancer Research, London, UK; c Department of Radiotherapy and Imaging, The Institute of Cancer Research, London, UK; d Department of Radiotherapy and Imaging, Royal Marsden NHS Foundation Trust and Institute of Cancer Research, London, UK; e Department of Oncology, Shrewsbury and Telford Hospital NHS Trust, Shrewsbury, UK; f Department of Clinical Oncology, Beatson West of Scotland Cancer Centre, Glasgow, UK; g Cancer Centre, University Hospitals of North Midlands and Keele University, Stoke-on-Trent, UK; h Department of Radiation Oncology, Auckland City Hospital, Auckland, New Zealand; i Department of Breast Surgery, Nuffield Health Cheltenham Hospital, Cheltenham, UK; j Department of Health and Medical Sciences, University of Surrey, Guildford, UK; k Department of Oncology, Norfolk and Norwich University Hospital NHS Foundation Trust, Norwich, UK; l Patient Advocate, London, UK; m Department of Research Oncology, King's College London, London, UK; n Cancer Centre, The Clatterbridge Cancer Centre NHS Foundation Trust, Bebington, UK; o Department of Radiotherapy, Mount Vernon Cancer Centre Northwood, Northwood, UK; p Department of Oncology, Royal Cornwall Hospitals NHS Trust, Truro, UK

## Abstract

**Background:**

Local cancer relapse risk after breast conservation surgery followed by radiotherapy has fallen sharply in many countries, and is influenced by patient age and clinicopathological factors. We hypothesise that partial-breast radiotherapy restricted to the vicinity of the original tumour in women at lower than average risk of local relapse will improve the balance of beneficial versus adverse effects compared with whole-breast radiotherapy.

**Methods:**

IMPORT LOW is a multicentre, randomised, controlled, phase 3, non-inferiority trial done in 30 radiotherapy centres in the UK. Women aged 50 years or older who had undergone breast-conserving surgery for unifocal invasive ductal adenocarcinoma of grade 1–3, with a tumour size of 3 cm or less (pT1–2), none to three positive axillary nodes (pN0–1), and minimum microscopic margins of non-cancerous tissue of 2 mm or more, were recruited. Patients were randomly assigned (1:1:1) to receive 40 Gy whole-breast radiotherapy (control), 36 Gy whole-breast radiotherapy and 40 Gy to the partial breast (reduced-dose group), or 40 Gy to the partial breast only (partial-breast group) in 15 daily treatment fractions. Computer-generated random permuted blocks (mixed sizes of six and nine) were used to assign patients to groups, stratifying patients by radiotherapy treatment centre. Patients and clinicians were not masked to treatment allocation. Field-in-field intensity-modulated radiotherapy was delivered using standard tangential beams that were simply reduced in length for the partial-breast group. The primary endpoint was ipsilateral local relapse (80% power to exclude a 2·5% increase [non-inferiority margin] at 5 years for each experimental group; non-inferiority was shown if the upper limit of the two-sided 95% CI for the local relapse hazard ratio [HR] was less than 2·03), analysed by intention to treat. Safety analyses were done in all patients for whom data was available (ie, a modified intention-to-treat population). This study is registered in the ISRCTN registry, number ISRCTN12852634.

**Findings:**

Between May 3, 2007, and Oct 5, 2010, 2018 women were recruited. Two women withdrew consent for use of their data in the analysis. 674 patients were analysed in the whole-breast radiotherapy (control) group, 673 in the reduced-dose group, and 669 in the partial-breast group. Median follow-up was 72·2 months (IQR 61·7–83·2), and 5-year estimates of local relapse cumulative incidence were 1·1% (95% CI 0·5–2·3) of patients in the control group, 0·2% (0·02–1·2) in the reduced-dose group, and 0·5% (0·2–1·4) in the partial-breast group. Estimated 5-year absolute differences in local relapse compared with the control group were −0·73% (−0·99 to 0·22) for the reduced-dose and −0·38% (−0·84 to 0·90) for the partial-breast groups. Non-inferiority can be claimed for both reduced-dose and partial-breast radiotherapy, and was confirmed by the test against the critical HR being more than 2·03 (p=0·003 for the reduced-dose group and p=0·016 for the partial-breast group, compared with the whole-breast radiotherapy group). Photographic, patient, and clinical assessments recorded similar adverse effects after reduced-dose or partial-breast radiotherapy, including two patient domains achieving statistically significantly lower adverse effects (change in breast appearance [p=0·007 for partial-breast] and breast harder or firmer [p=0·002 for reduced-dose and p<0·0001 for partial-breast]) compared with whole-breast radiotherapy.

**Interpretation:**

We showed non-inferiority of partial-breast and reduced-dose radiotherapy compared with the standard whole-breast radiotherapy in terms of local relapse in a cohort of patients with early breast cancer, and equivalent or fewer late normal-tissue adverse effects were seen. This simple radiotherapy technique is implementable in radiotherapy centres worldwide.

**Funding:**

Cancer Research UK.

**Research in context**

**Evidence before this study**

A comprehensive literature search using PubMed and MEDLINE was done before the trial opened to identify all previous pathological and clinical breast radiotherapy studies investigating patterns of recurrence within the ipsilateral breast, and also to identify results of previous partial-breast radiotherapy studies. Search terms included “early breast cancer”, “partial irradiation”, and “partial breast radiotherapy”. Existing research suggests that most local relapses occur in the vicinity of the original tumour bed and that older trials testing partial-breast radiotherapy were uninformative because of suboptimal patient selection, poor localisation of the tumour, and hence, inaccurate radiotherapy. We hypothesised that partial-breast radiotherapy using modern methods of radiotherapy planning and treatment would be non-inferior in terms of local relapse incidence and might have reduced normal-tissue toxicity in a low-risk of relapse population. This formed part of our peer-reviewed funding application for the trial.

**Added value of this study**

IMPORT LOW is the first phase 3 trial reporting 5-year outcome data for local relapses and adverse effects after partial-breast radiotherapy delivered using standard external beam radiotherapy techniques, and is the only trial, to the best of our knowledge, testing the importance of treatment volume unconfounded by radiotherapy dose-time factors. Additionally, the study is unique because it includes very comprehensive patient-reported outcome measures.

At 5 years, partial-breast radiotherapy delivered using a simple and standard technique, showed no increase in local relapse rates compared with whole-breast radiotherapy, and produced equivalent or reduced late adverse effects. Follow-up is continuing and 10-year local relapse incidence and toxicity will be reported in future.

**Implications of all the available evidence**

IMPORT LOW has similar local relapse incidence to the recently reported GEC-ESTRO brachytherapy partial-breast radiotherapy trial that also confirmed non-inferiority of partial-breast versus whole-breast radiotherapy. Our method of partial-breast radiotherapy seems to be safe and effective and has a key advantage of being relatively simple compared with conformal or inverse-planned intensity-modulated radiotherapy or brachytherapy. The use of standard medial and lateral tangential beams also minimises the mean heart dose without the need for breath hold in most patients with left-sided breast cancer, given that most patients have tumours in the upper half of the breast and above the level of the heart. Implementation of this technique will not require additional resources or training in most countries worldwide.

## Introduction

Breast radiotherapy after breast-conserving surgery has been shown to reduce the risk of any recurrence of breast cancer by a half and breast cancer-related mortality by a sixth in patients with early breast cancer.^1^ Whole-breast radiotherapy is the standard of care in the UK and internationally.^2^, ^3^, ^4^, ^5^ Current treatment guidelines discuss partial-breast radiotherapy for selected patients at low risk of recurrence because of age, small tumour size, and early stage, the evidence for which comes mainly from retrospective and prospective cohort studies in patients who received treatment using the MammoSite system and long-term results of a single, small, well conducted randomised trial of interstitial brachytherapy.^6^, ^7^, ^8^, ^9^, ^10^

One challenge in treating patients with early breast cancer is to reduce the morbidity of radiotherapy without compromising its ability to cure the cancer. The rationale for investigating partial-breast radiotherapy is based on international reports of reductions in local relapse incidence, and the recognition that the majority of ipsilateral local relapses occur close to the region of the index tumour (the so-called tumour bed).^11^, ^12^ Rapid technical advances in radiotherapy combined with accurate localisation of the tumour bed using titanium surgical clips enable more precise matching of radiotherapy dose intensity to the spatial variation in local relapse risk. Precise matching can now be achieved using a linear accelerator.^13^, ^14^, ^15^ This approach is predicted to have fewer chronic adverse effects than whole-breast radiotherapy, given the lower exposure of organs at risk, including breast tissue, ribcage, lung, and heart, without loss of local tumour control. Thousands of patients are currently being followed up in randomised studies, but long-term data (5 years or older) are available for few patients.^7^, ^16^, ^17^, ^18^ We report 5-year results of the first phase 3 trial testing partial-breast radiotherapy using a standard external beam technique and delivered after complete local tumour excision of low-risk early breast cancer.

## Methods

### Study design

IMPORT LOW is a multicentre, randomised, controlled, phase 3, non-inferiority trial comparing the safety and efficacy of standard whole-breast radiotherapy (control, whole-breast group) with experimental schedules of radiotherapy to the whole breast and partial breast (reduced-dose group), and to the partial breast only (partial-breast group). For the study protocol, see appendix pp 11–85. All treatment groups received simple forward-planned intensity-modulated radiation techniques (IMRT) to optimise dose homogeneity. In addition to the main study, two substudies addressing late adverse effects were done in a subset of centres, including photographic assessments of the breast and comprehensive patient-reported outcomes; centres declared upfront whether they wished to participate in the substudies. Patients were recruited to the substudies from the participating centres until the planned sample size had been obtained, and separate consent was given for the main trial and substudies. The study was approved by the Oxfordshire Research Ethics Committee B (06/Q1605/128) and done in accordance with the principles of Good Clinical Practice.

### Participants

Women who were aged 50 years or older who had breast-conserving surgery for unifocal invasive ductal adenocarcinoma (excluding invasive carcinoma of classical lobular type) of any grade (1–3) were recruited. Other inclusion criteria were pathological tumour size 3 cm or less (pT1–2), axillary node negative or one to three positive nodes (pN0–1), and minimum microscopic margins of non-cancerous tissue of 2 mm or more. Patients were not eligible if they had distant metastases, a previous malignancy of any kind (unless non-melanomatous skin cancer), undergone a mastectomy, or received neoadjuvant chemotherapy or concurrent adjuvant chemoradiotherapy. Primary endocrine therapy was allowed as long as the tumour was less than 3·0 cm, all other inclusion criteria were met, and breast-conserving surgery had been done. Eligibility criteria were amended twice during the trial. Women with grade 3 tumours or tumours with a diameter greater than 2 cm, or both, were excluded before a protocol amendment (approved March 4, 2008). A subsequent amendment (approved May 7, 2009) allowed inclusion of lymphovascular invasion and patients with one to three positive nodes (pN1; original criteria were that patients were node negative). The reduced local relapse incidence in the START trial^19^ and other recent studies compared with older trials indicated that broadening of the eligibility criteria was safe.^11^ All patients provided written informed consent. The study was sponsored by The Institute of Cancer Research. The Institute of Cancer Research Clinical Trials and Statistics Unit (ICR-CTSU; London, UK) was responsible for study management and central statistical data monitoring and all analyses. The Trial Management Group was responsible for day-to-day running of the trial and was overseen by an independent trial steering committee (TSC) and interim data reviewed confidentially by an independent data monitoring committee (IDMC). Patient advocates were involved at every stage of the trial, from initial study design through to preparation of the final manuscript.

### Randomisation and masking

Women were randomly assigned (in a 1:1:1 ratio) to receive conventional whole-breast radiotherapy or one of the two experimental schedules (reduced-dose or partial-breast radiotherapy). To randomly assign a patient, research staff at the centres telephoned ICR-CTSU to obtain the treatment allocation and trial ID number. Computer-generated random permuted blocks (mixed sizes of six and nine) were used to assign patients to groups, stratifying patients by radiotherapy treatment centre. Treatment allocation was not masked from patients, clinicians, or those analysing the data.

### Procedures

**Figure 1:**
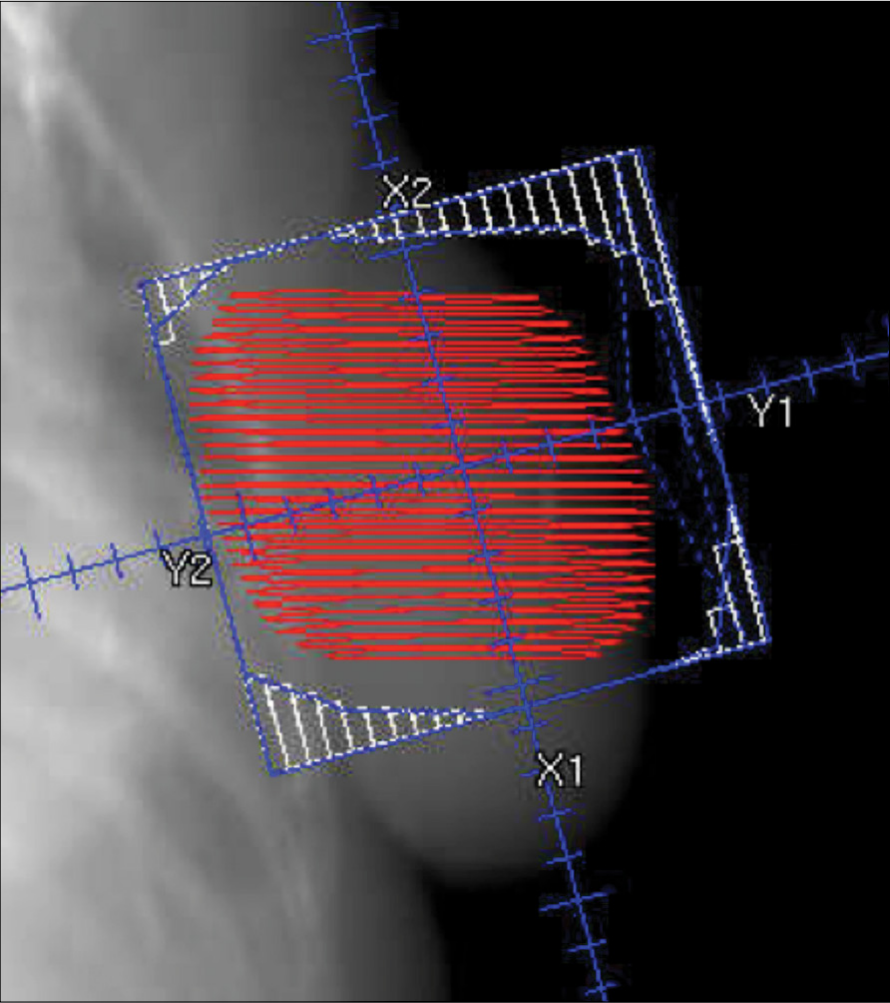
Radiotherapy technique for partial-breast group Red shows the partial-breast planning target volume and blue shows the radiotherapy field arrangements shaped with multileaf collimators. See appendix p 9 for further details.

Patients assigned to whole-breast radiotherapy (control) received 40 Gy in 15 fractions to the whole breast, those assigned to the reduced-dose group received 36 Gy in 15 fractions to the whole breast and 40 Gy in 15 fractions to the partial breast containing the tumour bed, and those assigned to the partial-breast group received 40 Gy in 15 fractions to the partial breast only. For localisation of the tumour bed, it was strongly recommended by the Trial Management Group to insert surgical clips, but if this was not possible, ultrasound, MRI, or CT was used.^13^, ^20^ If one of the recommended localisation procedures could not be done, entry into the study was permissible if the clinician was confident that clinical localisation was accurate—eg, if an obvious palpable tissue deficit was detected (appendix p 2).^13^, ^20^ The protocol specified forward-planned field-in-field IMRT delivered by standard medial and lateral tangential beams reduced in length but not in width. Non-target breast tissue medial or lateral to the planning target volume was thereby included in the high-dose zone (figure 1). Details of contouring and planning are described in the IMPORT LOW radiotherapy planning pack, which was used in addition to the clinical protocol (appendix p 1) and developed in partnership with the UK Radiotherapy Trials Quality Assurance (RTTQA) team. Each centre completed an initial questionnaire to establish details of their intended technique. Additionally, the RTTQA team visited each radiotherapy centre before opening of recruitment to independently validate the technique in use against the information given in the questionnaire. Measurements were made of the treatment volume with a purpose-made breast phantom, with particular reference to dose homogeneity. All plans together with corresponding CT datasets were collected electronically and stored at the RTTQA repository. Additionally, a subset of approximately one in ten patients (every tenth patient enrolled) were selected at randomisation to have thermoluminescence dosimetry measurements, which were also sent to the RTTQA team.

After radiotherapy, patients were scheduled for annual follow-up for 10 years. The mammography schedule was followed according to local practice, and was typically done annually for the first 5 years and then every 3 years as part of the national screening programme. Normal-tissue effects were assessed by clinicians, patients, and using photographs. Clinicians assessed breast shrinkage, distortion, induration, breast oedema, and telangiectasia at 1, 2, 5, and 10 years using a four-point scale (not at all, a little, quite a bit, or very much), comparing the ipsilateral breast with the contralateral breast when relevant.^21^ The assessment after 1 year was only required after protocol amendment (approved March 4, 2008). For the photographic substudy, photographs were taken at baseline (after surgery and before radiotherapy), at 2 years, and at 5 years.^22^ Patients in the patient-reported outcomes substudy completed the European Organisation for Research and Treatment of Cancer (EORTC) QLQ-C30 core questionnaire, EORTC QLQ-BR23 breast cancer module, body-image scale, protocol-specific questions (has skin appearance changed, overall breast appearance changed, breast become smaller, breast become harder or firmer to touch, or is shoulder stiffness present?), Hospital Anxiety and Depression Scale, and the EuroQol EQ-5D-3L health status questionnaire. These were scheduled at baseline (before randomisation), 6 months, and 1, 2, and 5 years. Symptomatic rib fracture, symptomatic lung fibrosis, and ischaemic heart disease incidence were recorded at 1, 2, 5, and 10-year follow-up.

### Outcomes

The primary outcome measure was local relapse, defined as the presence of any invasive or non-invasive carcinoma in any location in the ipsilateral breast parenchyma or overlying skin, assessed at each centre. Secondary efficacy outcomes were location of local tumour relapse, time to regional relapse (axilla, supraclavicular fossa, and internal mammary chain), time to distant relapse, disease-free survival (an event was defined as any local, regional, or distant relapse, contralateral breast cancer, or death due to breast cancer), overall survival, contralateral breast cancers, and other second primary cancers. Secondary outcomes relating to late-onset normal-tissue effects were assessed by clinicians for all patients, and also by patients and from photographs in the substudies.

Patient-reported outcomes focused on key items (arm or shoulder and breast) from the EORTC QLQ-BR23 module and protocol-specific questions that were recorded on the same 4-point scale as for the clinician assessments (not at all, a little, quite a bit, or very much). This manuscript reports on selected items from the BR23 breast cancer module and protocol-specific questions that correspond to clinician-reported assessments. Further analysis of patient-reported outcomes will be reported separately.

Digital photographs were scored as showing no change (none), mild, or marked change in breast appearance at 2 and 5 years compared with baseline by three observers (CC, AK, and JRY) using a previously described and validated consensus method.^22^ These observers were masked to treatment allocation but not to year of follow-up.

### Statistical analysis

The trial was powered to assess non-inferiority of the cumulative incidence of local relapse for each of the experimental groups compared with the control group. A 2·5% incidence of local relapse at 5 years was assumed with whole-breast radiotherapy, and the trial aimed to show that an increase of more than 2·5% in the cumulative incidence of local relapse would not occur in either experimental group. 645 patients were needed in each group to give 80% power with an α of 2·5% (one sided), allowing for 5% of patients to be lost to follow-up by 5 years. A target number of events was not stated in the protocol but data maturity was reviewed and discussed by the IDMC and TSC. The IDMC considered data to be sufficiently mature once at least 80% of forms were returned at 5 years.

The photographic substudy required 400 patients per group to have more than 90% power to detect at least a 10% difference in change of overall breast appearance for each experimental group compared with control (two-sided α of 0·025). With 400 patients per group, the patient-reported outcome substudy had more than 80% power to detect differences of at least 15% in the prevalence of normal-tissue effects (two-sided α of 0·005 to allow for multiple testing) and allowing for 10% attrition (due to death or illness). The same 0·005 threshold for significance was used for the clinician-reported normal-tissue effects.

Survival analysis methods were used to compare efficacy outcomes between the control group and experimental schedules with time measured from randomisation. For time to local relapse, patients were censored at death or at final follow-up for those who had no events. For distant relapse, disease-free survival, and overall survival, patients who had no events were censored at final follow-up. Nelson-Aalen cumulative hazard functions were plotted by treatment group.

Kaplan-Meier analyses were used to estimate event rates at 5 years with 95% CIs. Estimates of treatment effect were made using unadjusted Cox regression models, with hazard ratios (HRs) less than 1 indicating a decreased risk of the event in the experimental group compared with the control group. Absolute treatment differences in local relapses were calculated on the basis of the Kaplan-Meier estimate of patients who did not have local relapse in the control group and the HR. Each experimental group could be considered non-inferior to the control group if the upper limit of the two-sided 95% CI for local relapse HR was less than 2·03 (critical HR; excluding an increase in local relapse from 2·5% to 5·0%). Superiority of each experimental group compared with the control could be tested if non-inferiority could be claimed (using a 0·025 significance level). Analyses were done in the intention-to-treat population. The primary outcome was also analysed in the per-protocol population (all patients who completed their protocol-defined radiotherapy regimen) because this was a non-inferiority trial.

Patient and clinician-reported late normal tissue effects were dichotomised for the analysis as none or a little versus quite a bit or very much (defined as none or mild versus moderate or marked). The proportion of late moderate or marked events at 5 years is reported for each clinician-reported and patient-reported late normal-tissue event. Fisher's exact tests were used to compare each experimental schedule with the control group. All analyses of late normal tissue effects were done on a modified intention-to-treat basis—ie, all patients with available data, according to randomised treatment allocation. Time to first moderate or marked event was analysed using Kaplan-Meier analysis. Patients with no events were censored at last assessment of normal tissues (by clinician or patient as appropriate) or death. For the patient-reported outcomes, the Cox model was adjusted for baseline scores. Photographic data is presented as the proportion of patients who had photographs taken, with no change (none) or mild or marked change in breast appearance at 2 and 5 years compared with baseline. The Fisher's exact test was used to compare each experimental schedule with the control group at both time points. There was no imputation of missing normal-tissue data.

For all time-to-event analyses, the proportional hazards assumption of the Cox model was tested using Schoenfeld residuals and found to hold. Analyses were based on a database snapshot taken on June 15, 2016, and done using STATA version 13. This study is registered in the ISRCTN registry, number ISRCTN12852634, and ClinicalTrials.gov, number NCT00814567.

### Role of the funding source

Cancer Research UK provided peer-reviewed approval for the trial but had no other role in study design, data collection, data analysis, data interpretation, or writing of the report. The corresponding author had full access to all the study data and had final responsibility for the decision to submit for publication. CLG and JMB also had full access to study data.

## Results

**Figure 2:**
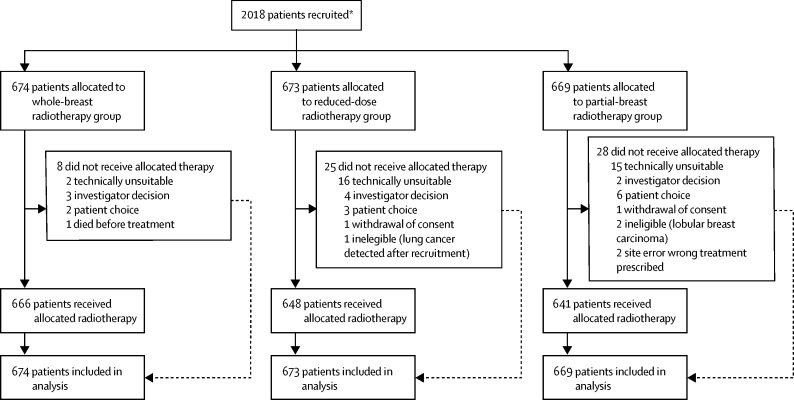
Trial profile *Two patients withdrew consent for any of their data to be used in the analysis.

Between May 3, 2007, and Oct 5, 2010, 2018 patients were recruited to the study. Two individuals withdrew consent for use of their data in the analysis; these two patients were removed from the intention-to-treat population. Patients were randomly assigned to either the whole-breast group (n=675), reduced-dose group (n=674), or the partial-breast group (n=669). Five patients were found to be ineligible after randomisation (three patients had lobular breast carcinoma, one had renal cell carcinoma, and one had lung cancer). Three of these patients did not receive their allocation treatment, but the other two were in the control group and so received standard treatment regardless. Seven patients did not receive any radiotherapy and 54 did not receive their allocated treatment (figure 2). 1482 (74%) of 2016 patients had surgical clips, 494 (25%) had imaging (either CT or ultrasound), and for 40 (2%), clinical methods alone were used to localise the tumour bed. Demographic and clinical characteristics were similar across the three treatment groups (table 1). 104 (5%) of 2016 women had chemotherapy, 1826 (91%) had endocrine therapy, and 36 (2%) had trastuzumab.

**Table 1:** Demographic and clinical characteristics at randomisation by treatment group (n=2016*)

		**Whole-breast radiotherapy (n=674)**	**Reduced-dose radiotherapy (n=673)**	**Partial-breast radiotherapy (n=669)**
Age, years		62 (57–67)	63 (57–67)	62 (57–67)
Side of primary tumour				
	Left breast	336/674 (50%)	344/673 (51%)	348/669 (52%)
	Right breast	338/674 (50%)	329/673 (49%)	321/669 (48%)
Pathological tumour size, cm†		1·2 (0·8–1·5)	1·1 (0·8–1·6)	1·2 (0·8–1·6)
Tumour grade‡				
	1	298/672 (44%)	272/673 (40%)	284/668 (43%)
	2	310/672 (46%)	328/673 (49%)	320/668 (48%)
	3	64/672 (10%)	73/673 (11%)	63/668 (9%)
Re-excision				
	Yes	93/673 (14%)	78/673 (12%)	87/667 (13%)
	No	580/673 (86%)	595/673 (88%)	580/667 (87%)
Axillary surgery				
	Yes	672/673 (>99%)	673/673 (100%)	666/667 (>99%)
	No	1/673 (<1%)	0	1/667 (<1%)
Pathological node status				
	Positive	24/674 (4%)	19/673 (3%)	16/669 (2%)
	Negative	650/674 (96%)	654/673 (97%)	653/669 (98%)
Histological type				
	Infiltrating ductal	578/671 (86%)	581/672 (86%)	563/665 (85%)
	Mixed	14/671 (2%)	18/672 (3%)	22/665 (3%)
	Other	79/671 (12%)	73/672 (11%)	80/665 (12%)
Lymphovascular invasion				
	Present	34/493 (7%)	47/492 (10%)	35/494 (7%)
	Absent	459/493 (93%)	445/492 (90%)	459/494 (93%)
ER status				
	Positive	640/672 (95%)	638/672 (95%)	633/667 (95%)
	Poor§	32/672 (5%)	34/672 (5%)	34/667 (5%)
PR status				
	Positive	400/493 (81%)	393/477 (82%)	380/475 (80%)
	Poor§	93/493 (19%)	84/477 (18%)	95/475 (20%)
HER2 status				
	Negative	599/622 (96%)	603/628 (96%)	580/614 (94%)
	Positive	23/622 (4%)	25/628 (4%)	34/614 (6%)
Adjuvant therapy received¶				
	Chemotherapy	29/673 (4%)	42/670 (6%)	33/665 (5%)
	Endocrine therapy	610/673 (91%)	614/670 (92%)	602/665 (91%)
	Trastuzumab	7/673 (1%)	15/670 (2%)	14/665 (2%)

Data are n/N (%) or median (IQR). N is total number of patients for whom the test result or measurement was available. ER=oestrogen receptor. PR=progesterone receptor.

* Two patients withdrew consent for any of their data to be used in analysis.

† Result unknown in one patient from partial-breast radiotherapy group.

‡ Tumours of two patients in the whole-breast group and one patient in the partial-breast group were ungradeable.

§ Poor refers to less than 10% receptor staining.

¶ Not mutually exclusive (ie, patients could have had more than one type of therapy).

**Figure 3:**
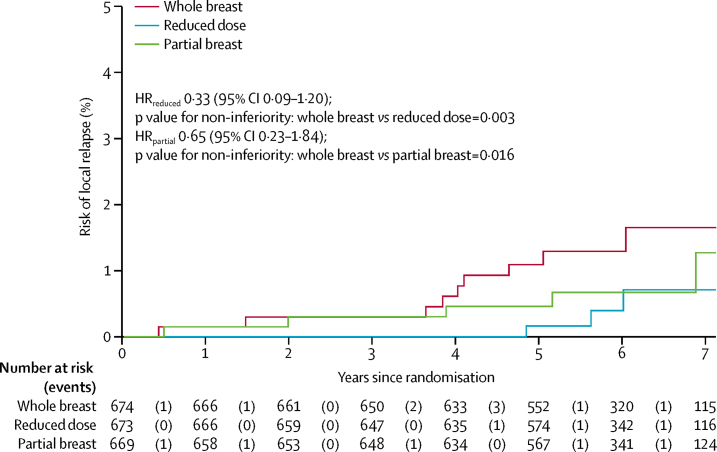
Cumulative hazard of local relapse by treatment group HR=hazard ratio.

After a median follow-up of 72·2 months (IQR 61·7–83·2), local relapse had been reported for 18 patients, nine (1%) of whom were in the whole-breast group, three (<1%) in the reduced-dose group, and six (1%) in the partial-breast group. 5-year estimated cumulative incidence of local relapse was 1·1% (95% CI 0·5–2·3) in the whole-breast group, 0·2% (0·02–1·2) in the reduced-dose group, and 0·5% (0·2–1·4) in the partial-breast group. The estimated absolute differences in local relapse by 5 years in the experimental groups compared with whole-breast radiotherapy at 5 years was −0·73% (95% CI −0·99 to 0·22) for the reduced-dose group and −0·38% (−0·84 to 0·90) for the partial-breast group. Since the upper limit of the two-sided 95% CI ruled out a greater than 2·5% increase in local relapse risk for each of the test schedules, non-inferiority can be claimed for both reduced-dose and partial-breast radiotherapy. Confirmation of this assertion is illustrated by a test against the critical HR greater than 2·03, with p=0·003 for the reduced-dose group and p=0·016 for the partial-breast group compared with the whole-breast radiotherapy group (table 2; figure 3). Analyses in the per-protocol population were consistent (p=0·003 for the reduced-dose group and p=0·017 for the partial-breast group; full data for per-protocol analyses not shown because treatment compliance was high). Local relapses occurred most frequently in patients with at least one high-risk feature (appendix p 86).

**Table 2:** Relapse and mortality by treatment group

	**Cumulative number of events, n/N (%)**	**5-year cumulative incidence, % (95% CI)**	Hazard ratio*(95% CI)	**p value**†
**Local relapse**				
Whole breast	9/674 (1%)	1·1% (0·5–2·3)	1	··
Reduced dose	3/673 (<1%)	0·2% (0·02–1·2)	0·33 (0·09–1·20)	0·077
Partial breast	6/669 (1%)	0·5% (0·2–1·4)	0·65 (0·23–1·84)	0·420
**Local-regional relapse**				
Whole breast	9/674 (1%)	1·1% (0·5–2·3)	1	··
Reduced dose	3/673 (<1%)	0·2% (0·02–1·2)	0·33 (0·09–1·21)	0·077
Partial breast	8/669 (1%)	0·8% (0·3–1·8)	0·88 (0·34–2·27)	0·761
**Distant relapse**				
Whole breast	13/674 (2%)	1·4% (0·7–2·6)	1	··
Reduced dose	10/673 (1%)	1·5% (0·8–2·8)	0·77 (0·34–1·75)	0·525
Partial breast	12/669 (2%)	1·6% (0·8–2·9)	0·92 (0·42–2·03)	0·838
**Any breast-cancer-related event**				
Whole breast	33/674 (5%)	3·7% (2·5–5·4)	1	··
Reduced dose	24/673 (4%)	3·4% (2·2–5·1)	0·72 (0·43–1·22)	0·223
Partial breast	33/669 (5%)	4·0% (2·8–5·9)	1·00 (0·62–1·62)	0·982
**All-cause mortality**				
Whole breast	40/674 (6%)	5·0% (3·6–7·0)	1	··
Reduced dose	39/673 (6%)	4·1% (2·8–5·9)	0·97 (0·62–1·50)	0·883
Partial breast	37/669 (6%)	3·7% (2·5–5·4)	0·91 (0·58–1·42)	0·693

* A hazard ratio of less than 1 favours the experimental group.

† Log-rank test, for each experimental group compared with whole-breast radiotherapy.

Four regional relapses were reported: one in the whole-breast group, one in the reduced-dose group, and two in the partial-breast group. Two of these relapses coincided with local relapse and two were isolated axillary relapses. Incidence of distant relapse, disease-free survival, and overall survival were similar across treatment groups, with low numbers of overall events and no statistically significant differences observed between experimental and control groups (table 2). 32 (2%) of 2016 patients developed invasive contralateral breast primary cancers: ten (1%) of 674 in the whole-breast group, 11 (2%) of 673 in the reduced-dose group, and 11 (2%) of 669 in the partial-breast group (table 3). Non-breast second primary cancers were reported for 96 (5%) of 2016 patients: 35 (5%) of 674 in the whole-breast group, 37 (5%) of 673 in the reduced-dose group, and 24 (4%) of 669 in the partial-breast group. Colorectal, lung, and gynaecological cancers were the most common. 18 of the 19 cases of lung cancer developed within 5 years of randomisation and similar numbers were ipsilateral and contralateral to the treated breast (appendix p 87).

**Table 3:** Local relapse, second cancers, and deaths by treatment group

		**Whole breast (n=674)**	**Reduced dose (n=673)**	**Partial breast (n=669)**	**Total (n=2016)**
Local relapse		9* (1%)	3† (<1%)	6 (1%)	18 (1%)
	Within radiotherapy field‡	9 (1%)	1 (<1%)	4 (1%)	14 (1%)
	Borderline with radiotherapy field	0	0	1 (<1%)	1 (<1%)
	Not documented	0	2 (<1%)	1 (<1%)	3 (<1%)
Contralateral breast second primary		12 (2%)	13 (2%)	13 (2%)	38 (2%)
	Invasive	10 (1%)	11 (2%)	11 (2%)	32 (2%)
	DCIS	2 (<1%)	2 (<1%)	2 (<1%)	6 (<1%)
Non-breast second primary		35 (5%)	37 (5%)	24 (4%)	96 (5%)
	Colorectal	10§ (1%)	7 (1%)	3 (<1%)	20 (1%)
	Lung	11§ (2%)	4 (1%)	4 (1%)	19 (1%)
	Gynaecological	5 (1%)	8 (1%)	4 (1%)	17 (1%)
	Other¶	4 (1%)	3 (<1%)	1 (<1%)	8 (<1%)
	Oesophagus	0	3 (<1%)	3 (<1%)	6 (<1%)
	Pancreas	1 (<1%)	2 (<1%)	3 (<1%)	6 (<1%)
	Lymphoma	0	2 (<1%)	3 (<1%)	5 (<1%)
	Genitourinary	3 (<1%)	1 (<1%)	0	4 (<1%)
	Head and neck	1 (<1%)	2 (<1%)	0	3 (<1%)
	Liver	0	2 (<1%)	1 (<1%)	3 (<1%)
	Cancer of unknown primary	0	0	2 (<1%)	2 (<1%)
	Peritoneal	0	2 (<1%)	0	2 (<1%)
	Sarcoma	1 (<1%)	1|| (<1%)	0	2 (<1%)
Deaths		40 (6%)	39 (6%)	37 (6%)	116 (6%)
	Breast cancer	9** (1%)	7†† (1%)	10‡‡ (1%)	26 (1%)
	Second cancer	14 (2%)	16 (2%)	12 (2%)	42 (2%)
	Cardiac	5 (1%)	2 (<1%)	2 (<1%)	9 (<1%)
	Cerebrovascular accident	1 (<1%)	2 (<1%)	1 (<1%)	4 (<1%)
	Pulmonary embolism	0	2 (<1%)	0	2 (<1%)
	Other	11 (2%)	10 (1%)	10 (1%)	31 (2%)
	Unknown	0	0	2 (<1%)	2 (<1%)

Data are n (%). DCIS=ductal carcinoma in situ.

* Two patients with DCIS.

† One patient with DCIS.

‡ No relapses were documented outside of the radiotherapy field.

§ One patient reported a colorectal second cancer followed by a lung second cancer and is included in both categories.

¶ Other includes adrenal, squamous cell carcinoma of the skin, melanoma, leukaemia, and mesothelioma.

|| Angiosarcoma developed in the treated breast.

** One patient with distant relapse before death died from mesothelioma.

†† One patient with distant relapse before death died from renal failure.

‡‡ Two patients with distant relapse before death also died from other causes, one sepsis and one cardiac related.

116 (6%) of 2016 patients died: 26 (1%) from breast cancer, 88 (4%) from other causes (including 42 [2%] from second cancers and nine [<1%] cardiac-related), and two (<1%) with unknown cause of death with no evidence of disease relapse before death (table 3). Numbers of cardiac deaths were similar between patients with left-side and right-sided breast cancers (appendix p 88).

In relation to normal-tissue effects, at the 5-year assessment, patients generally reported fewer moderate or marked events for the protocol-specific questions (skin change, overall breast appearance change, breast smaller, and breast harder or firmer to touch) in the partial-breast group than in the whole-breast group (table 4), although this reduction was statistically significant for change in breast appearance only (p<0·0001). At 5 years, change in breast appearance had the highest cumulative incidence of items reported as moderate or marked by patients in all groups. Reports of breast becoming harder or firmer were significantly reduced in both the reduced-dose group (p=0·002) and partial-breast group (p<0·0001) compared with the whole-breast group. Cumulative incidence of reports of the breast becoming harder or firmer were higher than the point prevalence at 5 years because this value included events reported earlier in follow-up, many of which were likely to be temporary post-surgical effects. The proportion of patients reporting any arm and shoulder symptoms as moderate or marked at 5 years was low in all groups with no significant differences for either experimental schedule compared with the control group. Similarly, cumulative incidence estimates indicated similar rates of arm and shoulder symptoms between groups.

**Table 4:** Patient assessments of moderate or marked late adverse events

		**Cumulative number of adverse events**			**Adverse events at 5 years**	
		n/N (%)	5-year cumulative incidence*, % (95% CI)	HR (95% CI), p value†	n/N (%)	p value‡
**Protocol-specific items**						
Breast appearance changed						
	Whole breast	158/411 (38%)	47·7% (41·1–54·8)	1	80/295 (27%)	··
	Reduced dose	123/433 (28%)	36·7% (30·6–43·6)	0·74 (0·54–1·00), p=0·051	66/325 (20%)	0·047
	Partial breast	113/421 (27%)	35·1% (28·7–42·5)	0·64 (0·46–0·89), p=0·007	49/331 (15%)	<0·0001
Breast smaller						
	Whole breast	119/411 (29%)	37·3% (30·9–44·4)	1	66/294 (22%)	··
	Reduced dose	110/433 (25%)	31·9% (26·3–38·4)	0·83 (0·59–1·16), p=0·280	63/326 (19%)	0·373
	Partial breast	104/421 (25%)	34·7% (27·5–43·0)	0·78 (0·54–1·11), p=0·162	56/331 (17%)	0·086
Breast harder or firmer						
	Whole breast	115/411 (28%)	35·3% (28·4–43·3)	1	27/292 (9%)	··
	Reduced dose	74/433 (17%)	21·0% (16·2–26·9)	0·53 (0·36–0·79), p=0·002	23/325 (7%)	0·376
	Partial breast	58/421 (14%)	15·3% (12·0–19·5)	0·47 (0·32–0·71), p<0·0001	15/330 (5%)	0·024
Shoulder stiffness						
	Whole breast	56/411 (14%)	19·3% (14·0–26·5)	1	12/296 (4%)	··
	Reduced dose	56/433 (13%)	19·3% (13·9–26·4)	0·93 (0·64–1·35), p=0·701	22/328 (7%)	0·161
	Partial breast	58/421 (14%)	15·3% (12·0–19·5)	1·06 (0·73–1·54), p=0·756	13/331 (4%)	0·999
Skin appearance changed						
	Whole breast	63/411 (15%)	21·0% (15·5–27·9)	1	22/294 (7%)	··
	Reduced dose	59/433 (14%)	17·9% (13·2–24·0)	1·07 (0·68–1·68), p=0·775	23/325 (7%)	0·878
	Partial breast	49/421 (12%)	14·6% (10·4–20·5)	0·87 (0·54–1·40), p=0·569	12/330 (4%)	0·051
**EORTC QLQ-BR23**						
Arm or shoulder pain						
	Whole breast	98/411 (24%)	32·6% (26·3–39·9)	1	33/297 (11%)	··
	Reduced dose	104/433(24%)	30·1% (24·7–36·4)	0·94 (0·71–1·25), p=0·678	43/329 (13%)	0·465
	Partial breast	97/421 (23%)	27·2% (21·9–33·6)	0·97 (0·73–1·28), p=0·809	24/331 (7%)	0·097
Swollen arm or hand						
	Whole breast	21/411 (5%)	6·2% (4·1–9·5)	1	5/295 (2%)	··
	Reduced dose	26/433 (6%)	9·8% (6·2–15·3)	1·19 (0·67–2·11), p=0·558	15/330 (5%)	0·066
	Partial breast	16/421 (4%)	4·4% (2·7–7·3)	0·59 (0·30–1·15), p=0·123	2/330 (1%)	0·264
Difficulty raising arm						
	Whole breast	42/411 (10%)	13·6% (9·2–19·8)	1	10/297 (3%)	··
	Reduced dose	45/433 (10%)	14·0% (9·8–19·8)	0·98 (0·64–1·50), p=0·913	17/328 (5%)	0·326
	Partial breast	47/421 (11%)	13·5% (10·1–18·0)	1·08 (0·71–1·64), p=0·726	15/331 (5%)	0·542
Breast pain						
	Whole breast	67/411 (16%)	19·1% (14·9–24·3)	1	13/295 (4%)	··
	Reduced dose	65/433 (15%)	16·9% (12·9–22·1)	0·96 (0·68–1·35), p=0·812	18/330 (5%)	0·584
	Partial breast	64/421 (15%)	18·2% (14·1–23·4)	0·96 (0·68–1·36), p=0·830	13/328 (4%)	0·842
Breast swollen						
	Whole breast	31/411 (8%)	8·1% (5·7–11·3)	1	1/295 (<1%)	··
	Reduced dose	26/433 (6%)	6·8% (4·7–9·9)	0·84 (0·49–1·41), p=0·503	4/329 (1%)	0·377
	Partial breast	17/421 (4%)	4·7% (2·9–7·6)	0·49 (0·27–0·89), p=0·019	1/328 (<1%)	0·999
Breast oversensitive						
	Whole breast	64/411 (16%)	17·2% (13·7–21·5)	1	9/296 (3%)	··
	Reduced dose	59/433 (14%)	16·5% (12·0–22·4)	0·89 (0·62–1·27), p=0·526	16/330 (5%)	0·308
	Partial breast	54/421 (13%)	18·3% (13·0–25·5)	0·80 (0·55–1·14), p=0·220	13/330 (4%)	0·665
Skin problems in breast						
	Whole breast	50/411 (12%)	15·7% (11·1–21·9)	1	7/296 (2%)	··
	Reduced dose	42/433 (10%)	13·4% (9·2–19·2)	0·78 (0·52–1·18), p=0·237	10/328 (3%)	0·632
	Partial breast	35/421 (8%)	9·2% (6·7–12·7)	0·64 (0·42–0·99), p=0·045	9/330 (3%)	0·806

EORTC=European Organisation for Research and Treatment of Cancer.

* Estimated at 5 years and 3 months.

† Wald test.

‡ Fisher's exact test.

1319 women consented to the photographic substudy, and baseline photographs were received and assessed for 1222 patients. Photographs taken at 2 years were assessed in 1000 women. The most common reasons for photographs not being available were centre administrative oversight so that photographic appointments were not made, patients not attending hospital visits, and patients withdrawing consent from the substudy. At 2 years, mild or marked changes in breast appearance were observed in 37 (11%) of 332 in the whole-breast group, 32 (10%) of 335 in the reduced-dose group, and 31 (10%) of 333 people in the partial-breast group. At 5 years, photographs were available for 805 women and, compared with the 2-year results, the proportion of patients with mild or marked changes had increased across all groups (whole-breast 60 (23%) of 262, reduced-dose 59 (22%) of 264, and partial-breast 50 (18%) of 279). No evidence of a statistically significant difference was seen in the proportion of patients with a change in breast appearance for either experimental schedule compared with whole-breast radiotherapy at 2 years (reduced-dose p=0·527; partial-breast p=0·446) or 5 years (reduced-dose p=0·917; partial-breast p=0·165).

Clinical assessment of late normal-tissue effects at 5 years showed a low occurrence of moderate or marked events across all treatment groups (table 5). At 5 years, breast shrinkage had the highest prevalence of moderate or marked events (whole-breast 41 (9%) of 452, reduced-dose 37 (8%) of 478, and partial-breast 33 (7%) of 472), whereas breast oedema was rare (whole-breast four (1%) of 446; reduced-dose two (<1%) of 468; partial-breast none of 468). The cumulative incidences also indicated breast shrinkage to be the most common late normal-tissue effect. The HRs for all late effects were consistently less than 1, but no evidence of statistically significant differences for individual events was seen. Severe late adverse effects were rare, and included four confirmed reports of rib fractures, eight of lung fibrosis, and five of ischaemic heart disease (appendix p 89).

**Table 5:** Clinician assessment of moderate or marked late adverse events

	**Cumulative number of adverse events**			**Adverse events at 5 years**	
	n/N (%)	5-year cumulative incidence*, % (95% CI)	HR (95% CI), p value†	n/N (%)	p value‡
**Worst normal-tissue effects**					
Whole breast	134/674 (20%)	27·6% (22·5–33·6)	1	60/457 (13%)	··
Reduced dose	108/673 (16%)	21·1% (17·2–25·7)	0·77 (0·60–0·99), p=0·043	48/480 (10%)	0·152
Partial breast	94/669 (14%)	20·0% (15·6–25·4)	0·69 (0·53–0·90), p=0·006	49/474 (10%)	0·221
**Breast shrinkage**					
Whole breast	79/674 (12%)	18·4% (13·7–24·5)	1	41/452 (9%)	··
Reduced dose	70/673 (10%)	13·6% (10·6–17·5)	0·86 (0·62–1·18), p=0·345	37/478 (8%)	0·480
Partial breast	61/669 (9%)	13·9% (10·1–19·0)	0·78 (0·56–1·08), p=0·134	33/472 (7%)	0·276
**Breast induration (index)**					
Whole breast	63/674 (9%)	12·7% (9·5–16·8)	1	21/453 (5%)	··
Reduced dose	43/673 (6%)	8·4% (6·0–11·6)	0·66 (0·45–0·98), p=0·040	13/474 (3%)	0·161
Partial breast	48/669 (7%)	10·8% (7·7–15·1)	0·77 (0·53–1·12), p=0·165	24/471 (5%)	0·762
**Breast induration (outside index)**§					
Whole breast	15/674 (2%)	2·3% (1·4–3·8)	1	2/450 (<1%)	··
Reduced dose	10/673 (1%)	2·1% (1·0–4·1)	0·66 (0·30–1·48), p=0·310	2/464 (<1%)	>0·999
**Telangiectasia**					
Whole breast	8/674 (1%)	1·6% (0·8–3·3)	1	3/445 (1%)	··
Reduced dose	8/673 (1%)	3·0% (1·3–6·8)	0·96 (0·36–2·57), p=0·976	6/468 (1%)	0·507
Partial breast	5/669 (1%)	0·6% (0·2–1·7)	0·62 (0·21–1·92), p=0·401	4/465 (1%)	>0·999
**Breast oedema**					
Whole breast	24/674 (4%)	4·0% (2·6–6·2)	1	4/446 (1%)	··
Reduced dose	18/673 (3%)	3·2% (2·0–5·3)	0·74 (0·40–1·37), p=0·338	2/468 (<1%)	0·441
Partial breast	11/669 (2%)	1·7% (0·9–3·0)	0·46 (0·23–0·94), p=0·029	0/468	0·056
**Other radiotherapy related**					
Whole breast	11/674 (2%)	1·7% (1·0–3·1)	1	3/457 (1%)	··
Reduced dose	9/673 (1%)	1·4% (0·7–2·6)	0·81 (0·34–1·97), p=0·646	0/480	0·263
Partial breast	6/669 (1%)	0·9% (0·4–2·0)	0·55 (0·20–1·49), p=0·234	0/474	0·221

HR=hazard ratio.

* Estimated at 5 years and 3 months.

† Log-rank test.

‡ Fisher's exact test.

§ No cases of moderate or marked breast induration (outside index) were reported in the partial-breast group.

## Discussion

Our 5-year results confirm that local relapse was scarce across all trial groups and that non-inferiority was shown for both partial-breast and reduced-dose radiotherapy. Late normal-tissue effects were also uncommon across all groups, and significantly fewer patients reported breast hardness in the partial-breast radiotherapy group compared with control. These findings support our hypothesis that partial-breast radiotherapy using a standard radiation technique can reduce late toxicity without jeopardising local tumour control.

IMPORT LOW is the only phase 3 trial of partial-breast radiotherapy to use the same dose-fractionation regimen and radiation technique in the whole-breast and partial-breast radiotherapy groups. Because the same regimen is used, differences in treatment outcome can be attributed more reliably to differences in radiotherapy volume. The Danish Breast Cancer Group phase 2 partial-breast radiotherapy trial is similarly designed to have breast volume as the only variable, but has a primary endpoint of grade 2 or higher breast induration at 3 years (Offersen B, Aarhus University, personal communication; NCT00892814). Other phase 3 partial-breast radiotherapy trials report a variety of different dose-fractionation regimens from a single intraoperative dose to 1–2 weeks of treatment.^18^, ^23^, ^24^ These differences make it challenging to distinguish whether variations in outcome are caused by differences in treated volume or radiation dose-time effects. This difficulty is illustrated by the interim results at 3 years from the RAPID trial^25^ (NCT00282035) that compared three-dimensional (3D) conformal partial-breast radiotherapy using 38·5 Gy in ten fractions over 5 days, with whole-breast radiotherapy using 42·5 Gy in 16 fractions or 50 Gy in 25 fractions with an optional boost. Cosmetic outcome and late normal-tissue toxicity were worse in the partial-breast radiotherapy group in the RAPID trial, which suggests that dose-time effects were the dominant factor over reduced irradiated volume within this study. Other randomised trials (NSABP NCT00103181, SHARE NCT01247233, IRMA NCT01803958) using similar dose-fractionation regimens to RAPID have yet to publish mature outcome data, although early reports suggest minor toxicity.

Another strength of IMPORT LOW is that patients were specifically engaged with the aim of producing the most comprehensive patient-reported outcomes in any published partial-breast radiotherapy trial to date. The patient's viewpoint is clearly important, but previous breast radiotherapy trials have shown that patient-reported outcomes are very sensitive when distinguishing between different dose-fractionation regimens.^26^ The results of IMPORT LOW suggest that patient-reported outcomes are also able to detect a radiotherapy volume effect, which is highly relevant for the design of future breast cancer radiotherapy trials, because patient-reported outcomes could be the most cost-effective yet sensitive and patient-centred method of outcome assessment. We analysed and presented the late normal-tissue toxicity for both patient-reported and clinician-reported outcomes both using discrete 5-year timepoints and cumulative incidence. The purpose of dual analysis is to convey different information, in that the longitudinal results capture the maximum grades of toxicity, whereas the cross-sectional 5-year results take into account that some side-effects were resolved, such as oedema, which might reduce over time. We acknowledge that multiple statistical tests were done for the normal-tissue toxicity analysis, but we accounted for this by using a stringent significance level of 0·005 for clinician-reported and patient-reported outcomes.

The simplicity of IMPORT LOW is also a strength. The partial-breast radiotherapy technique uses standard tangential fields that are simply shortened to encompass the tumour bed and margin of healthy tissue. This technique means that a larger volume of breast is treated than with other 3D conformal or IMRT and brachytherapy techniques, but tangential beams minimise dose to surrounding organs at risk such as the heart and lungs by keeping the exit beams within the breast. This method might be important in minimising second radiation-induced cancers. It might also minimise the mean heart dose without the need for breath-hold in most patients with left-sided breast cancer, given that most patients have tumours in the upper half of the breast and above the level of the heart.^27^, ^28^ The tangential field arrangement is more likely to deliver at least some dose to the lower axilla in comparison with more conformal partial-breast radiotherapy techniques that are likely to deliver none, which might be important in minimising axillary recurrences.^29^ A simple form of forward-planned IMRT was used to optimise dose homogeneity, but this is now standard in most centres,^30^, ^31^ so implementation of this technique does not require additional resources or training in most countries.

The original estimates of local relapse on which the sample size was based were high, given recent general improvements in local tumour control.^11^ Retrospective power calculations, based on year 5 data being available for 1832 (91%) of 2016 patients and an observed local relapse 5-year cumulative incidence in the control group of 1·1% confirm that a clinically relevant absolute 2·0% increase in 5-year local relapse could be excluded for each test group, assuming 80% power and 2·5% α (one-sided). The demonstration of non-inferiority is expected to be stable with longer follow-up, although proportion of patients with local relapse in IMPORT LOW is likely to be in the range of 1–3% by 10 years. This expectation is based on the ELIOT trial^23^ in which the cumulative incidence of local relapse in the intraoperative group rose in an apparently linear fashion between 5 and 9 years. Compliance with photographic assessments was not as high as anticipated in IMPORT LOW. However, given the few reported changes in breast appearance at 5 years in the control group (23%), retrospective power calculations indicate that this photographic substudy has 75% power to detect a difference of 10% (with a 2·5% significance level).

Furthermore, our study might have been limited by biased reporting of late normal-tissue toxicity because treatment allocation could not be masked. However, the panel of assessors doing the photographic assessments were masked to treatment groups, although photographic assessments seem to be less sensitive to subtle changes in normal-tissue toxicity than patient-reported assessments.

A major question raised by this trial is which patients should be selected for partial-breast radiotherapy? IMPORT LOW was originally designed to recruit patients with very low-risk disease; however, eligibility criteria were widened during recruitment to include some slightly higher-risk features after the publication of evidence of low recurrence rates from other breast radiotherapy trials, such as START.^19^ However, looking at the baseline characteristics in IMPORT LOW, most of the women who were recruited had small, low-grade, ER-positive, node-negative tumours. The appendix (p 86) shows that despite the low proportion of patients with high-risk disease, these patients had eight of the 18 local relapses. However, this observation should be taken with caution because the overall number of events was low. The UK has taken a pragmatic approach to patient selection for partial-breast radiotherapy by producing a consensus statement,^32^ which states that partial-breast radiotherapy can be considered for patients who are 50 years or older, with grade 1–2 cancer, a tumour of 30 mm or less, ER positive, HER2 negative, and N0 with minimum 1 mm radial excision margins for invasive disease. Given the small proportion of participants in IMPORT LOW who were node positive, we support the UK Breast Radiotherapy Consensus in not recommending partial-breast radiotherapy for this group. Consistent with the findings of ACOSOG Z0011,^33^ IBSCG 23–01,^34^ NCIC MA20,^35^ and EORTC 22922,^36^ we recommend that patients who are node positive receive whole-breast radiotherapy as standard of care.

A further controversy raised by this and other reported studies,^37^ is the definition of ipsilateral local relapse. For example, the IMPORT LOW definition is recurrence of any preinvasive or invasive carcinoma in the ipsilateral breast regardless of histology or location of the index breast cancer. The GEC-ESTRO trial^8^ definition does not take into account location within the breast, but does exclude tumours with differing histology, and the Cochrane review^37^ only includes relapses within the index quadrant with the same histology. Clearly, inclusion or exclusion of local relapses could make a substantial difference in reported results given the low number of events in this patient group.

Finally, the results of IMPORT LOW are not consistent with the 2016 overview by the Cochrane Collaboration^37^ that was based on the published data of phase 3 trials, six of which contributed to analyses of local relapses and four to analyses of toxicity endpoints. This overview reported inferior results for both local relapse and late normal-tissue toxicity with partial-breast radiotherapy. The small number of contemporary partial-breast radiotherapy trials described in the Cochrane report^37^ might explain the difference between the findings.^31^ Four other phase 2 trials testing partial-breast radiotherapy are yet to report 5-year results (NSABP/RTOG NCT00103181, RAPID NCT00282035, SHARE NCT01247233, and IRMA NCT01803958). The mature results from over 10 000 patients recruited within these important trials will add to the literature in future.

The results from as yet unpublished partial-breast radiotherapy trials are clearly needed, but because of the huge heterogeneity in dose-fractionation regimen, radiotherapy technique, irradiated volume, and inconsistencies in the definition of ipsilateral breast tumour recurrence, these data might prove challenging to interpret. A large individual patient data meta-analysis might resolve this potential dilemma and we strongly support this initiative.

We also recognise the importance of investigating possible effects of partial-breast radiotherapy on the development of radiation-induced second cancer and major cardiac events. However, this research will require thousands of patients followed up for many years before robust conclusions can be made and might be best achieved by future interrogation of routine health data.

Another approach is to investigate the biology of local relapse and its relationship to partial-breast radiotherapy. For example, what constitutes a true ipsilateral recurrence from an ipsilateral new primary at the molecular level is still unclear and requires further investigation.

At 5 years, partial-breast radiotherapy delivered using a simple intensity-modulated technique achieved non-inferiority in incidence of local relapse compared with whole-breast radiotherapy and similar or reduced late adverse effects. This method of partial-breast radiotherapy seems to be safe and effective and could be implemented easily within most radiotherapy centres worldwide.
