# Supplementary material for: Partial-breast radiotherapy after breast conservation surgery for patients with early breast cancer (UK IMPORT LOW trial): 5-year results from a multicentre, randomised, controlled, phase 3, non-inferiority trial
Source: Lancet. 2017 Sep 9;390(10099):1048–60. doi: 10.1016/S0140-6736(17)31145-5 (PMC5594247; doi:10.1016/S0140-6736(17)31145-5)
Supplement: Supplementary appendix [file mmc1.pdf]

THE LANCET

Supplementary appendix

This appendix formed part of the original submission and has been peer reviewed. We post it as supplied by the authors.

Supplement to: Coles CE, Griffin CL, Kirby AM, et al, on behalf of the IMPORT Trialists. Partial-breast radiotherapy after breast conservation surgery for patients with early breast cancer (UK IMPORT LOW trial): 5-year results from a multicentre, randomised, controlled, phase 3, non-inferiority trial. *Lancet* 2017; published online Aug 2. http://dx.doi.org/10.1016/S0140-6736(17)31145-5.

Appendix 1 – IMPORT LOW Radiotherapy Planning Pack

Planning Pack for the IMPORT LOW Trial

A guide to outlining and planning IMPORT LOW patients

Introduction

IMPORT strongly encourages a move towards volume-based CT planning with 3D dose compensation. However it is understood that some centres may opt to use a simulator-based planning method, at least initially, so this document sets out guidelines for each of these approaches. It is recommended that some form of tumour bed localisation is used: surgical clips, gold seeds, ultrasound or MRI. For centres using surgical clips, it is recommended to place a pair of clips at the medial, lateral, superior, inferior, anterior and posterior extent of the tumour bed. Placing the clips in pairs overcomes the potential problem of clips migrating from the tumour site. Most importantly, the same method of localisation, outlining and planning must be adopted for each of the three test arms. If it is initially impossible to adopt one of the recommended procedures, it is permissible to enter patients if the clinician is confident that localisation is accurate in a particular case, for example, if an obvious tissue deficit is palpable in the treatment position.

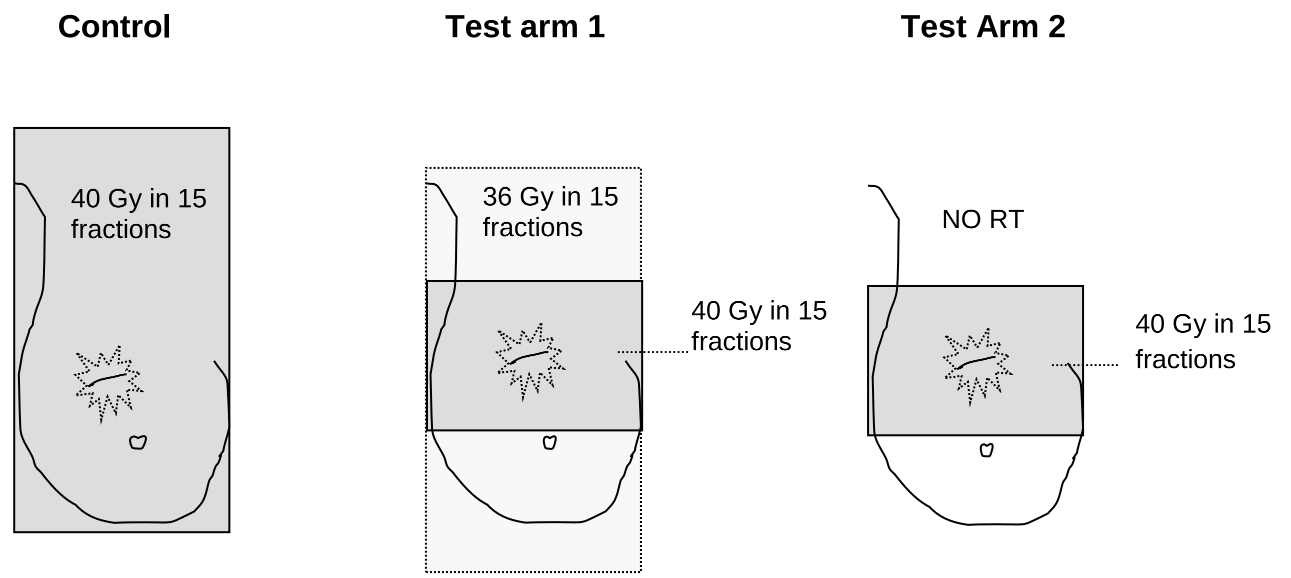

CT-based approach

Whole breast voluming on CT requires outlining a CTV volume on each axial slice, adding an appropriate PTV margin, and then a margin for field penumbra. This is the principle IMPORT is working towards, however it can be very difficult to accurately delineate breast tissue, and this can result in an overestimate of the whole breast volume. Therefore an alternative strategy is described here which still takes into account all of the CT information.

Example 1 – Right breast, tumour bed close to medial border

The planner/doctor selects a provisional field to cover the breast tissue and minimise dose to the normal tissues by scrolling up and down the CT dataset, as shown in the images below.

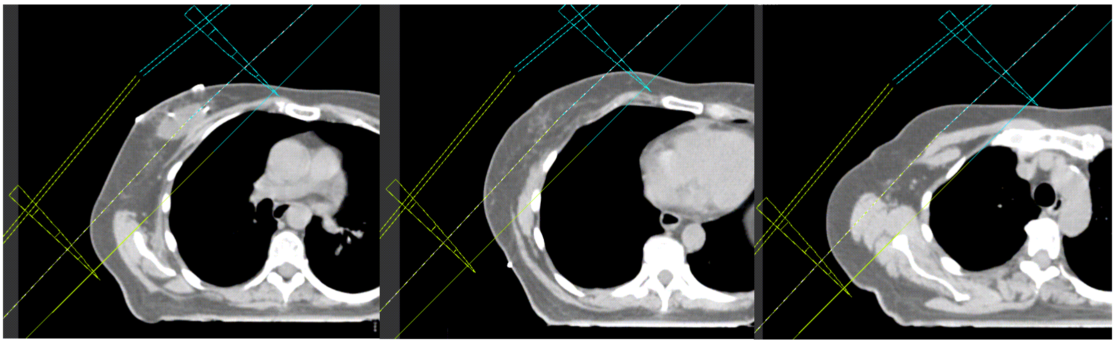

Central axis 10cm superior 10cm inferior

Centres may wish to derive a field-based whole breast PTV at this stage. Although this is not a true PTV, it is helpful for reporting purposes. The volume needs to be 5 mm from the skin surface, 5 mm from the posterior field edge and lung/chest wall interface, and 10 mm from the superior and inferior field edges:

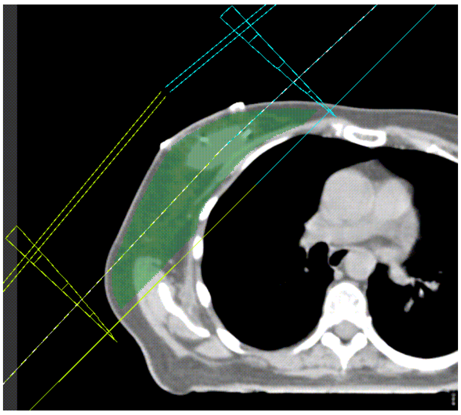

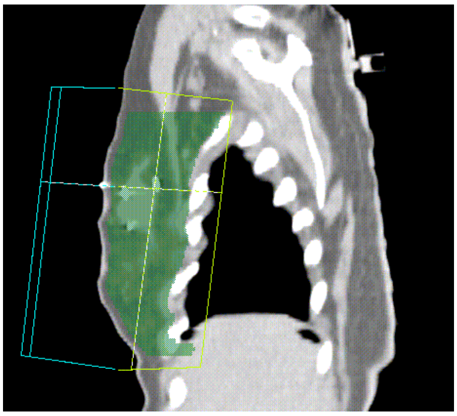

The clinician then outlines the tumour bed. This patient has gold seeds sutured into the tumour bed, and the volume is drawn around the seeds and any change in the surrounding tissue architecture. The same principle applies for patients with surgical clips.

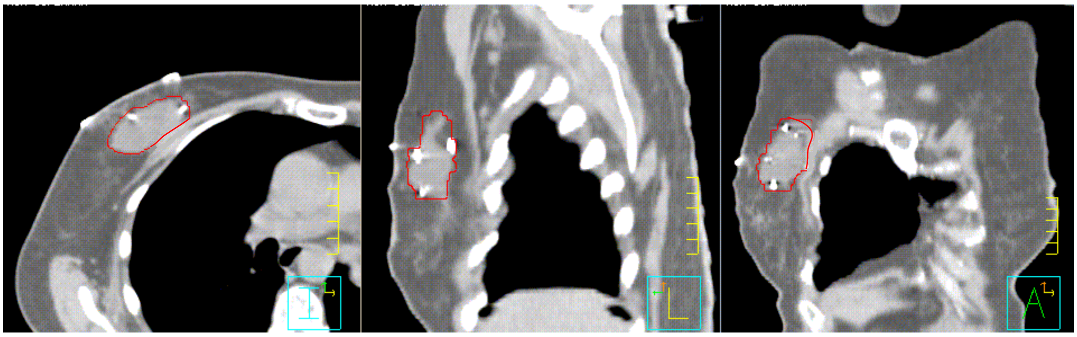

This is grown by 15 mm to give the partial breast CTV, bound by 5mm from the skin surface and should not extend beyond the pectoral fascia posteriorly. If the pectoral fascia is not visible, then it should be no more than 5mm from the lung/chest wall interface.

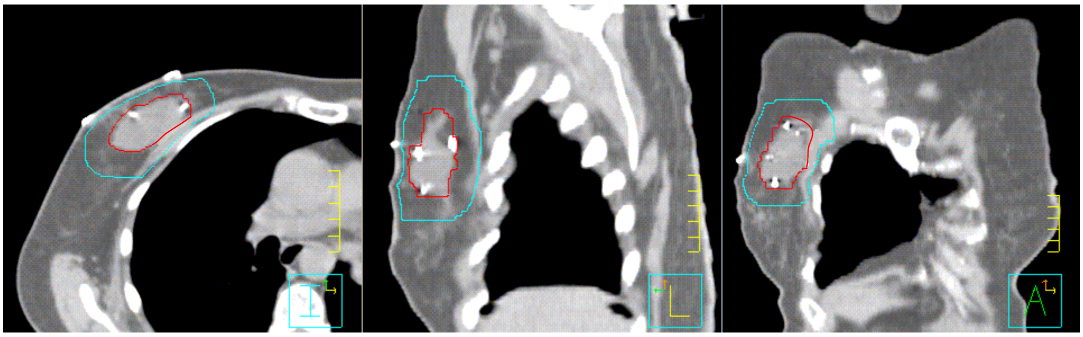

A PTV margin is then added (usually 10mm) to give the partial breast PTV, bound by 5mm from the skin surface but unmodified posteriorly:

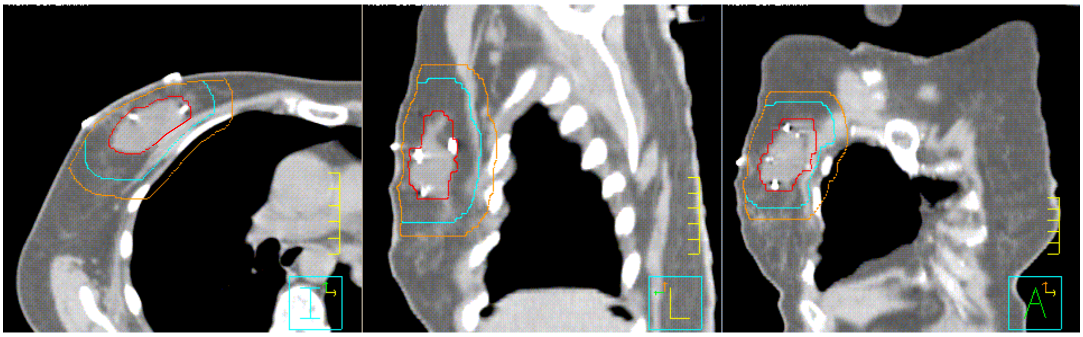

The clinician can then assess whether the field border needs to be changed based on the clinical information. In this case the fields positioned earlier cover the partial breast PTV well and no modifications are necessary.

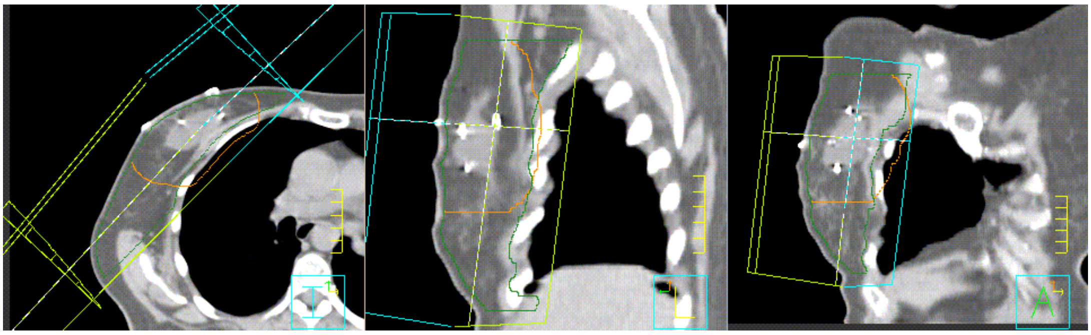

Example 2 – Right breast, tumour bed close to lateral border

The provisional field borders are placed as in the previous example by scrolling up and down the CT dataset to cover the breast tissue. The tumour bed is outlined and grown to generate the partial breast CTV and PTV:

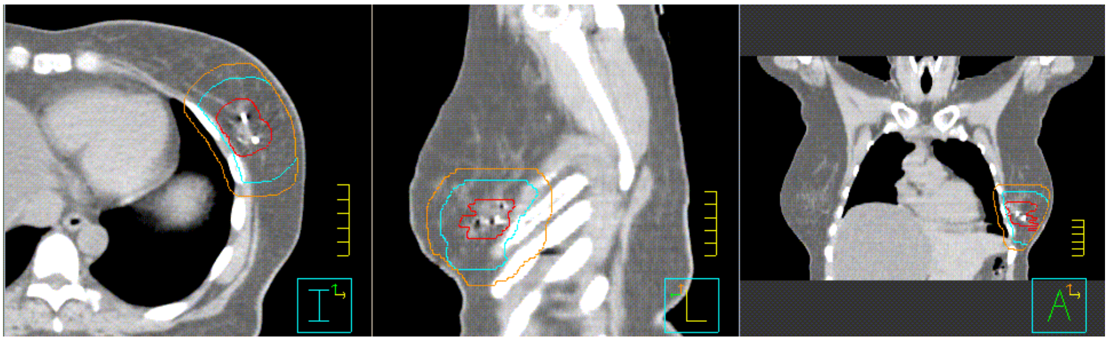

The images below show that the fields positioned earlier do not provide adequate coverage to the partial breast PTV. When using implanted markers there will usually be a marker at the deep fascia as the surgeon takes a core of tissue, which in some cases will cause the partial breast PTV to extend outside of the previously defined whole breast volume at the posterior edge:

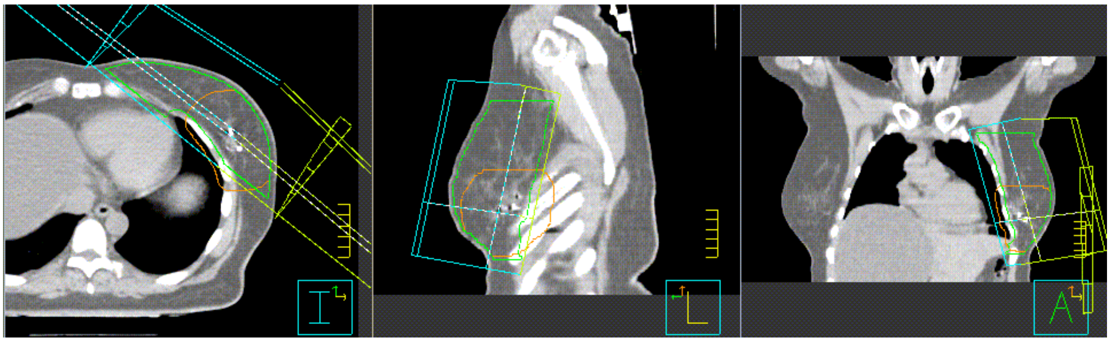

The histology report will give the tumour-free margin which will assist the modification of fields. In this case taking into account all relevant clinical information both the medial and lateral borders are modified to provide better coverage to the partial breast PTV:

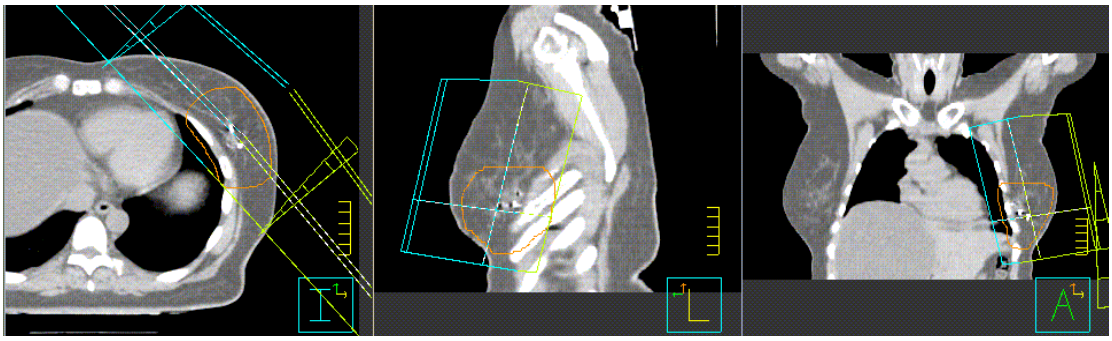

For all patients care should be taken when modifying the field borders that the dose to normal tissues and organs at risk is not significantly increased. The heart should be excluded from the fields if possible and the maximum lung depth should not exceed 2 cm (as per START).

Simulator-based approach

It is possible to satisfy requirements of the trial using a combination of simulator films and a minimum of 5 outlines, provided that either surgical clips or gold seed markers have been implanted. The whole breast fields can be simulated according to local practice for conventional breast patients. The partial breast PTV can then be determined by taking an image orthogonal to the tangent fields. Mark the medial extent of the PTV at 25 mm from the most medial clip or seed visible. Repeat for the lateral, superior and inferior

boundaries. The posterior edge is defined to be the same as that for the whole breast tangents. Using this image it should now be possible to draw a partial breast PTV onto the outlines, and planning can proceed as for the CT-based approach.

Planning & Dose Compensation

Field directions are to be tangential for all arms of the trial, and 3D dose compensation must be used if required. Compensation may take the form of missing tissue compensators based on outlines or portal images, or intensity modulated fields generated by forward or inverse planning. Sufficient uniformity is usually achieved with 3 or 4 segments per beam. The image below shows a beam's eye view of a medial tangent field and segment field. The whole breast PTV is shown in lilac and the partial breast PTV in red.

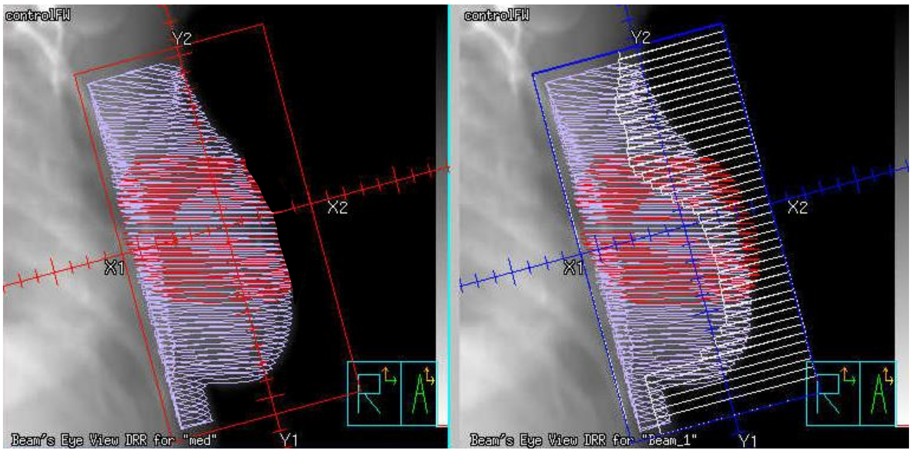

Below left a projection of the dose clouds in the beam's eye view shows that using simple 3D compensation can produce very small hot volumes. The image below right illustrates that using wedged tangents optimised on the central axis with no compensation can produce very large areas of 105% and 107% away from the central axis.

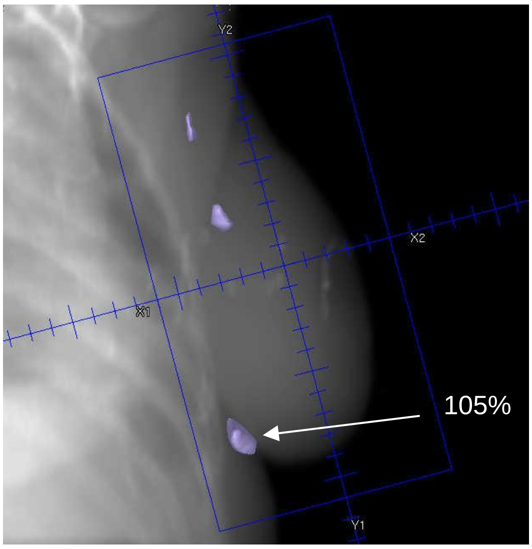

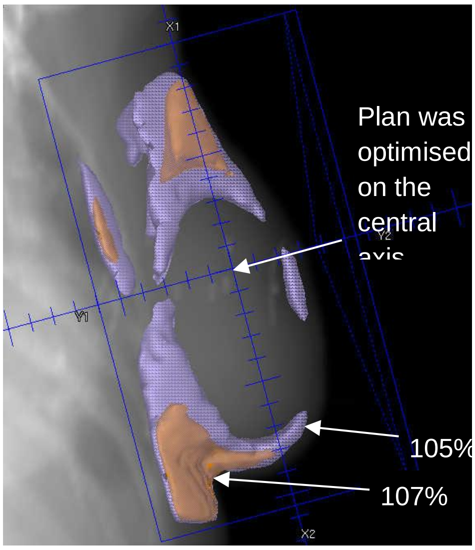

As with all IMRT planning, the dose to a single point such as the isocentre may no longer be representative of the median dose to the target and plans are evaluated with the help of DVHs. Plans are satisfactory when the 95% isodose covers the PTV and the hot 'volumes' are sufficiently small. In the example below the 95% is green and the 100% red.

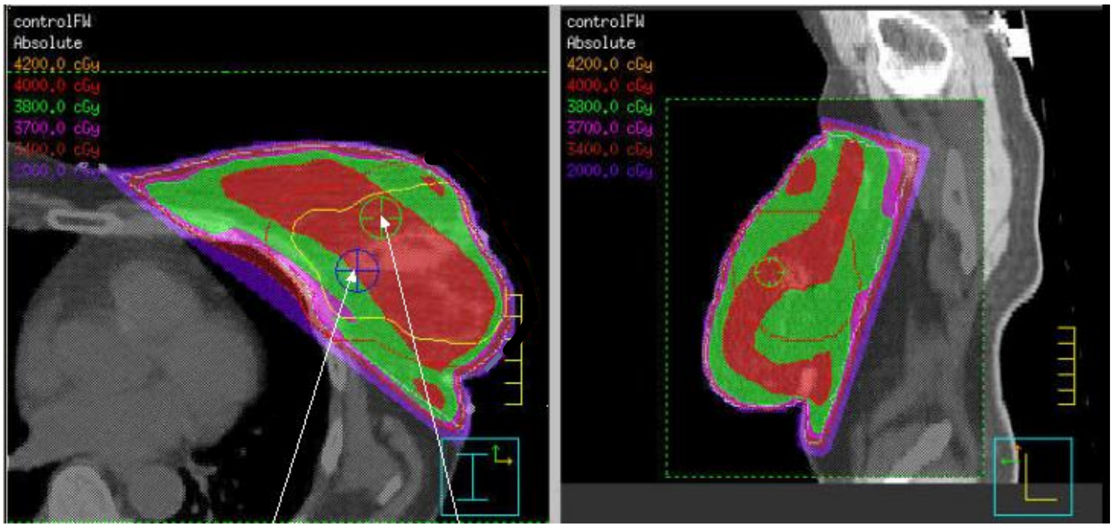

Prescription point Isocentre

6 MV will usually be used, however since we have parallel opposed fields no amount of compensation will reduce the entry doses if the patient separation is more than about 22cm. In these cases a higher energy (10MV) or mixed energies should be considered.

Control arm

The aim of the control arm is to deliver a uniform dose of 40Gy in 15 fractions to the whole breast, ensuring that the whole breast PTV is surrounded by 38 Gy (95%) and that the hot volume (approximately 2cc) is less than 42 Gy (105%) and definitely less than 42.8 Gy (107%).

Test arm 1

The aim of test arm 1 is to deliver a uniform dose 36Gy in 15 fractions to the whole breast, ensuring that the whole breast PTV is surrounded by 34.2Gy, and concurrently deliver a uniform dose of 40Gy to the 'cylinder' containing the partial breast PTV using small tangential fields. Unless inverse planning is used it is recommended to generate the plan in two stages - first obtain a uniform dose to the whole breast PTV. It can help to slightly bias the hot volume to the posterior part of the partial breast volume. Then add a pair of unwedged tangential fields to the partial breast PTV. These mini-tangent fields will have the same anterior

and posterior borders as the whole breast tangents, and in the superior/inferior direction will need no margin for field penumbra to achieve good 95% coverage. MLC shielding is permitted to shape the volume but not required:

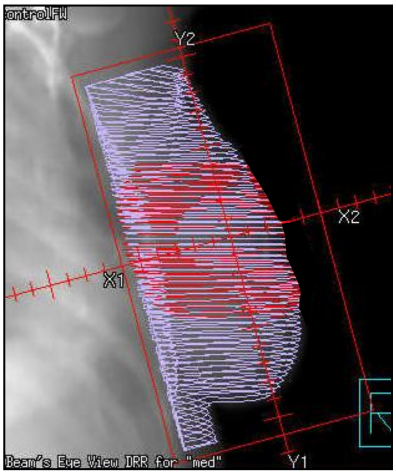

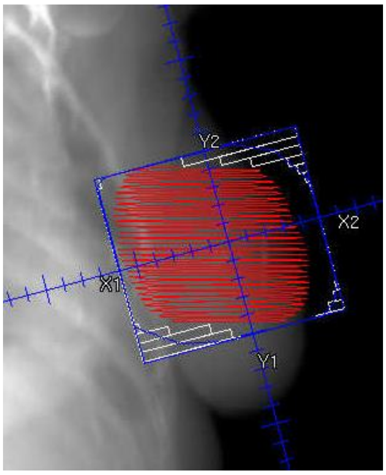

Test arm 2

The aim of test arm 2 is to deliver a uniform dose of 40Gy to the ‘cylinder’ containing the partial breast PTV using small tangential fields only. For test arm 2 it is often possible to achieve sufficient uniformity using wedged tangential fields, but compensation may sometimes be required. It is important that the 95% isodose encompasses the partial PTV, so the field lengths in test arm 2 will be typically 10mm longer than in test arm 1 to achieve the same coverage:

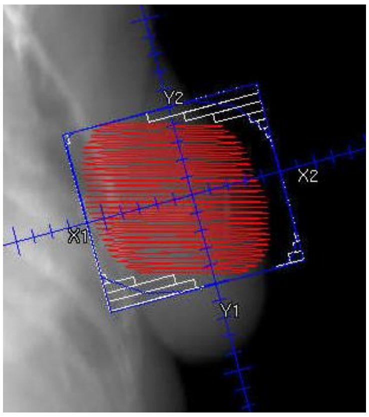

Test arm 1

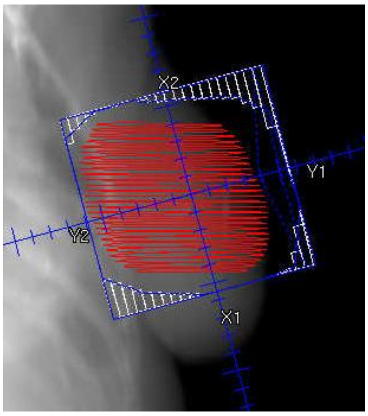

Test arm 2

For each of the test arms planners should aim to achieve the following DVH parameters:

**Whole breast PTV**	≥90% of volume should receive 95% of prescription dose
**Partial breast CTV**	≥95% of volume should receive 95% of prescription dose

These guidelines should enable reasonable coverage of the whole breast and partial breast volumes for most patients. In some cases this may not be possible, particularly for very medial or lateral tumour beds. Careful adjustments can be made to the field borders taking into account all the available clinical information to optimise target coverage. However the maximum lung depth **must not** exceed 2cm.

<u>**Appendix 2 – IMPORT LOW Protocol**</u>

IMPORT LOW

(Intensity Modulated and Partial Organ Radiotherapy)

Randomised Trial Testing Intensity Modulated and Partial Organ Radiotherapy following Breast Conservation Surgery for Early Breast Cancer

On behalf of the IMPORT LOW trial working party

Chief Investigator: Professor John Yarnold (Institute of Cancer Research/The Royal Marsden Hospital NHS Foundation Trust)

Chief Clinical Co-ordinator: Dr Charlotte Coles (Addenbrookes Hospital NHS Trust)

Clinical Trials and Statistics Unit, Institute of Cancer Research

ISRCTN12852634

ICR-CTSU/2006/10001

MREC No.:Oxford Research Committee B 06/Q1605/128

ICR
The Institute of Cancer Research

General correspondence to:	Clinical correspondence to:	or:
Jenny Titley<br>ICR-CTSU*<br>Section of Clinical Trials<br>Sir Richard Doll Building<br>Cotswold Road<br>Sutton, Surrey, SM2 5NG, UK<br>Tel: +44 (0) 20 8722 4104<br>Fax: +44 (0) 20 8770 7876<br>email: import-icrctsu@icr.ac.uk	Professor John Yarnold<br>Institute of Cancer Research<br>Royal Marsden Hospital<br>Downs Road<br>Sutton, Surrey,SM2 5PT, UK<br>Tel: 020 8661 3388<br>Fax: 020 8643 8809<br>Email: john.yarnold@icr.ac.uk	Dr Charlotte Coles<br>Box 193<br>Addenbrooke's NHS Trust<br>Hills Road<br>Cambridge, CB2 2QQ, UK<br>Tel: 01223 596182<br>Fax: 01223 274409<br>Email:charlotte.coles@addenbrookes.nhs.uk

Institute of Cancer Research Clinical Trials & Statistics Unit

This clinical trial protocol is intended to provide guidance and information only for the conduct of the IMPORT Trial in participating centres. It is not for use as a guide for the management of other patients outside of the trial.

If you have an urgent clinical query please contact Professor John Yarnold on 020 8661 3388 or Dr Charlotte Coles on 01223 596182.

The IMPORT trial has been scientifically approved by the Clinical Trials Awards and Advisory Committee (CTAAC) of Cancer Research and is thus part of the NCRN/NCRI portfolio of breast cancer and radiotherapy trials.

The IMPORT trial is sponsored by: The Institute of Cancer Research

Registered Office: 123 Old Brompton Road, London SW7 3RP, UK Tel: +44 (0) 20 7352 8133

IMPORT LOW Trial - FINAL PROTOCOL MREC VERSION 6.0 27th April 2009

Approved by:

Professor John Yarnold
Chief Investigator

Charlotte Coles
Chief Clinical Co-ordinator

TRIAL MANAGEMENT GROUP

Dr Rajiv Agrawal	Consultant Clinical Oncologist, Royal Shrewsbury Hospital
Dr Abdulla Alhasso	Consultant Clinical Oncologist, Western Infirmary
Prof. Judith Bliss	Director, ICR-CTSU*
Dr Murray Brunt	Consultant Clinical Oncologist, University Hospital of North Staffordshire
Mr Charlie Chan	Consultant Surgeon, Cheltenham General Hospital
Dr Charlotte Coles	Consultant Oncologist, Addenbrooke's Hospital
Ms Laura Ciurlionis	IMPORT QA Physicist, Mount Vernon Hospital
Dr John Dewar	Consultant Radiotherapist and Oncologist, Ninewells Hospital
Dr Ellen Donovan	Physicist, The Royal Marsden Hospital NHS Foundation Trust
Mrs Pat Eagle	Patient Representative
Mr Stephen Ebbs	Consultant Surgeon, Mayday University Hospital
Prof. Ian Ellis	Reader in Pathology, Nottingham City Hospital
Dr Phil Evans	Physicist, The Royal Marsden Hospital NHS Foundation Trust
Dr Adrian Harnett	Consultant Clinical Oncologist, Norfolk and Norwich University Hospital
Mrs Jo Haviland	Statistician, ICR-CTSU*
Dr Penny Hopwood	Psycho Oncologist, Christie Hospital
Mrs Helen Mayles	Head of Dosimetry, Clatterbridge Centre for Oncology
Ms Judith Mills	Trial Co-ordinator, ICR-CTSU*
Mr Andrew Poynter	Head of Medical Physics, Ipswich Hospital
Mrs Christine Rawlings	Research Radiographer, Torbay Hospital
Dr Elinor Sawyer	Dept. Health Clinician Scientist and Consultant Clinical Oncologist, Guy's Hospital; Cancer Research UK
Prof. Mark Sculpher	Health Economist, University of York
Mr Georges Sumo	Trial Statistician, ICR-CTSU*
Mr Mark Sydenham	Trial Co-ordinator, ICR-CTSU*
Dr Isabel Syndikus	Consultant Clinical Oncologist, Clatterbridge Centre for Oncology
Mr Alastair Thompson	Consultant Surgeon, Ninewells Hospital
Mrs Jenny Titley	Trial Co-ordinator, ICR-CTSU*
Mr Yat Tsang	IMPORT QA Radiographer, Mount Vernon Hospital
Dr Andrew Tutt	Consultant Oncologist, Clinician Scientist, Guy's Hospital; Breakthrough Breast Cancer Research Centre
Dr Nicola Twyman	Physicist, Addenbrooke's Hospital
Dr Karen Venables	Physicist, Mount Vernon Hospital
Dr Duncan Wheatley	Consultant Clinical Oncologist, Royal Cornwall Hospital
Mrs Maggie Wilcox	Patient Representative
Mr John Winstanley	Consultant Surgeon, Royal Bolton Hospital
Mr Gordon Wishart	Consultant breast and endocrine surgeon, Addenbrooke's Hospital
Prof. John Yarnold	Consultant Clinical Oncologist, The Royal Marsden Hospital NHS Foundation Trust

**** Institute of Cancer Research Clinical Trials and Statistics Unit***

TABLE OF CONTENTS Page No

TRIAL MANAGEMENT GROUP **3**

TRIAL SUMMARY **7**

TRIAL SCHEMA **9**

1. TITLE **10**

2. BACKGROUND **10**
2.1 Breast cancer mortality and iatrogenic morbidity can be reduced further 10
2.2 Radiotherapy target volume and dose intensity are poorly adapted to local recurrence risk 10
2.3 Low and high risk subgroups can be distinguished 10
2.4 Whole breast radiotherapy is unnecessary in women with low risk disease (< 1% annual risk of local recurrence risk after surgery, systemic therapy and whole breast radiotherapy) 13
2.5 A modest dose reduction outside the index quadrant will reduce late morbidity markedly without compromising tumour control 14
2.6 Localisation of the tumour bed needs to be improved in all patients 15
2.7 Full radiotherapy dose compensation should replace traditional techniques in all patients 15
2.8 Intensity modulated radiotherapy to the breast exploits widely available technologies 15

3. AIM **16**

4. ELIGIBILITY **16**
4.1 Inclusion criteria 16
4.2 Exclusion criteria 16

5. RANDOMISATION **16**
5.1 Treatment arms 17
5.2 Entry procedure 17

6. RADIOTHERAPY TARGET VOLUMES, LOCALISATION & OUTLINING **17**
6.1 Target volume definition 17
6.2 Tumour bed localisation 18
6.3 Patient position 19
6.4 Outlining 19

7. RADIOTHERAPY PLANNING **20**
7.1 Control arm 20
7.2 Test arm 1 geometry 20
7.3 Test arm 2 geometry 21
7.4 Suggested technique for field definition using a simulator: IMPORT LOW test arms 21
7.5 Inhomogeneity correction 21
7.6 Dose prescription 21
7.7 Organ at risk sparing: cardiac shielding 21
7.8 Bolus to scar 21
7.9 Beam energy 22

8. RADIOTHERAPY VERIFICATION 22

9. RADIOTHERAPY QUALITY ASSURANCE 22

10. FOLLOW-UP 23

11. ENDPOINTS 23
11.1 Tumour-related endpoints 23
11.2 Treatment-related endpoints 24
11.3 Serious Adverse Events 25

12. ANALYSIS 25
12.1 Choice of principal endpoints 25
12.2 Methods of analysis 25
12.3 Sample size 27
12.4 Interim analyses and data monitoring 27
12.5 Publication and presentation 27

13. RESEARCH GOVERNANCE 27
13.1 Trial administration and logistics 27
13.1.1 Chief Investigator 28
13.1.2 ICR-CTSU responsibilities 28
13.1.3 Participating centres responsibilities 28
13.2 Investigator training 28
13.3 Case Report Forms 28
13.4 Protocol compliance/ on site monitoring 28
13.5 Trial Management 29
13.5.1 Trial Management Group 29
13.5.2 Trial Steering Committee 29
13.5.3 Data Monitoring and Ethics Committee 30
13.6 End of study 30
13.7 Archiving 30
13.8 Publishing policy 30

14. CONFIDENTIALITY AND LIABILITY 30
14.1 Risk Assessment 30
14.2 Liability/Indemnity/Insurance 31
14.3 Patient confidentiality 31

15 ETHICAL CONSIDERATIONS 31

16 WITHDRAWAL OF PATIENTS FROM STUDY TREATMENT 32

17 FINANCIAL MATTERS 32

18. ASSOCIATED STUDIES 32
18.1 Molecular correlates of normal tissue injury 32
18.2. Molecular analysis of primary tumour, ipsilateral and contralateral recurrenece and new primary tumours 33

18.3. Quality of Life and Health Economics Studies 33

19. ETHICS COMMITTEE APPROVAL 34

20. PATIENT INFORMATION 34

APPENDICES 35

APPENDIX 1 Partial Breast Irradiation 35

APPENDIX 2 Equivalent total doses used in the IMPORT LOW trial 37

APPENDIX 3 Radiotherapy target volumes 38

APPENDIX 4 Localisation of the post-operative breast tumour cavity 41

APPENDIX 5 Suggested simple method for breast compensation using 1-2 extra MLC segments 44

APPENDIX 6 Treatment verification 45

APPENDIX 7 Quality assurance programme 47

APPENDIX 8 Serious Adverse Event Reporting 50

APPENDIX 9 Quality of life studies 51

APPENDIX 10 Evaluation of the economic consequences 63

APPENDIX 11 Recommendations for recurrence mapping 68

REFERENCES 70

TRIAL SUMMARY

Title

Randomised Trial Testing Intensity Modulated and Partial Organ Radiotherapy following Breast Conservation Surgery for Early Breast Cancer.

Aim

To test partial breast radiotherapy delivered using intensity modulated techniques following complete local tumour excision in women with low risk early stage breast cancer.

Inclusion criteria

***All* of the following:**

- Age ≥ 50 years.
- Primary breast conservation surgery +/- adjuvant systemic therapy.
- Pathological tumour size ≤ 3.0 cm pT1-2 (< 3.1 cm, maximum microscopic diameter of invasive component).
- Invasive adenocarcinoma (excluding invasive carcinoma of classical lobular type).
- Unifocal disease.
- Grade I, II or III.
- Lymphovascular invasion present or absent
- Axillary lymph nodes negative or 1 to 3 nodes positive (pN0 or pN+(1-3))
- Minimum microscopic margin of non-cancerous tissue ≥ 2 mm (excluding deep margin if this is at deep fascia).
- No blood-borne metastases.

Exclusion criteria

- Previous malignancy (other than non-melanomatous skin cancer).
- Mastectomy.
- Invasive carcinoma of classical lobular type.
- Primary endocrine therapy or chemotherapy (neo-adjuvant endocrine therapy is permissible as long as the tumour is <3.0 cm and all other inclusion criteria are met. Primary endocrine therapy as a replacement for surgery is not permissible).
- Concurrent chemo-radiotherapy.

Study design

Prospective randomised controlled clinical trial for patients at low risk of local recurrence (< 1% annual risk local recurrence after radiotherapy).

Radiotherapy delivery

The control arm delivers 40 Gy in 15 fractions to whole breast (no boost). Two test arms are proposed: Test Arm 1: 2.4 Gy x 15 to low risk areas and 2.67 Gy x 15 to the region of the primary tumour, and Test Arm 2: 2.67 Gy x 15 to the region of the primary tumour; breast tissue outside the region of the primary tumour is excluded from the target volume.

Endpoints

The primary endpoint is local tumour control in the ipsilateral breast. Secondary endpoints include location of tumour relapse, contralateral primary tumours, regional and distant metastases, late adverse effects in normal tissues, quality of life (QL) and economic evaluation.

Sample size

Assuming a 2.5% recurrence rate at 5 years in the control arm, 613 patients per randomised group would provide 80% power to exclude an increase of > 2.5% in either test arm ($\alpha = 0.025$; 1-sided as testing for non-inferiority, and allowing for the 1:1:1 randomisation). Allowing for a 5% rate of loss to follow-up by 5 years, including loss due to disease progression, (based on experience in the START trial) gives 645 required in each arm, and the trial will therefore recruit a total of 1,935 patients. Photographic assessment and quality of life studies will only be required in a prospective sample of 1,200 patients.

IMPORT LOW TRIAL SCHEMA

Female age ≥ 50
Primary breast conservation surgery +/- adjuvant systemic therapy for early breast cancer
tumour ≤ 3.0cm pT1-2 (<3.1 cm, maximum microscopic diameter of invasive component).

⇩

Other Inclusions:
Invasive adeno-carcinoma, unifocal, Grade I, II or III,
minimum margin ≥ 2mm,
lymphovascular invasion present or absent,
axillary lymph nodes negative or 1 to 3 nodes positive (pN0 or pN+ (1-3)),
no blood borne metastases

⇩

Exclusions:
Previous malignancy (other than non-melanomatous skin cancer)
Mastectomy
Invasive carcinoma of classical lobular type
Primary endocrine therapy or chemotherapy (neo-adjuvant endocrine therapy permissible if tumour <3.0 cm and all other inclusion criteria are met. Primary endocrine therapy as a replacement for surgery is not permissible).
Concurrent chemo-radiotherapy

⇩

Patient eligible for IMPORT LOW and consents to participate?

⇩

Centres NOT participating in sub-studies
- **Family history questionnaire and blood sample collection**

Centres participating in Sub-studies
- **Family history questionnaire and blood sample collection**
- **Baseline Quality of Life and Health Economics questionnaires**
- **Photographs of breasts following surgery**

⇩

Randomise to one of three treatments by telephoning: 020 8643 7150 (09.00-17.00 Mon-Fri)

⇩

Standard Treatment
Standard radiotherapy to whole breast
Monday-Friday
3 weeks,15 treatments

Test Treatment 1
Reduced radiotherapy to whole breast
Standard radiotherapy to partial breast
Monday-Friday
3 weeks,15 treatments

Test Treatment 2
Standard radiotherapy to partial breast
Monday-Friday
3 weeks,15 treatments

⇩

Patient Follow Up

All Centres
- **Follow up all patients annually for 10 years**
- **Paraffin embedded tissue collection from primary tumours and any recurrence**

Centres participating in Sub-studies
- **Follow up all patients annually for 10 years**
- **Paraffin embedded tissue collection from primary tumours and any recurrence**
- **Follow-up Quality of Life and Health Economics questionnaires at: 6 months, 1 year, 2 years and 5 years**
- **Photographs of breasts at 2 years and 5 years**

1. TITLE

Randomised Trial Testing Intensity Modulated and Partial Organ Radiotherapy following Breast Conservation Surgery for Early Breast Cancer.

2. BACKGROUND

2.1 Breast cancer mortality and iatrogenic morbidity can be reduced further

The Early Breast Cancer Trialists Collaborative Group (EBCTCG) systematic overview confirms a 75% reduction in local tumour recurrence risk after radiotherapy in patients treated by breast conservation surgery for early breast cancer (1). The overview shows that the prevention of 4 local tumour recurrences prevents 1 breast cancer death at 10 years, corresponding to 1 – 5 fewer deaths per 100 node negative women and 5 – 10 fewer deaths per 100 node positive patients treated. These observations are strengthened by the 2000 systematic overview of radiotherapy effects by the EBCTCG (2).

For the majority of women with small screen-detected cancers, late complications of radiotherapy rather than local cancer recurrence are now the dominant hazard. Exposure of non-target tissues to high doses of radiotherapy predisposes to a wide range of morbidities, including lung fibrosis, heart injury (left-sided cases), rib fracture, shoulder stiffness, breast shrinkage, tissue hardness, pain and tenderness with consequent effects on physical functioning and quality of life (3, 4). Complication risk is increased by the widespread use of radiotherapy planning techniques based on standard wedge compensators that fail to meet international dosimetric guidelines in a majority of patients (5, 6) (7). The morbidity of radiotherapy would be significantly reduced by eliminating unplanned dose variation across the breast and reducing the dose to parts of the breast remote from the tumour bed at very low risk of recurrence.

2.2 Radiotherapy target volume and dose intensity are poorly adapted to local recurrence risk

Standard guidelines for radiotherapy in early breast cancer fail to discriminate between different risk groups. Women with low-grade screen-detected cancers a few millimetres in diameter receive the same radiotherapy regimen as women with higher risk tumours. They share the same risk of complications as women with high risk tumours, but have less to gain in terms of local tumour control. For women with low risk disease, the challenge is to reduce the morbidity of therapy without compromising cure. Applications of recent technical advances in radiotherapy should achieve more effective exploitation of radiotherapy dose-response, fractionation sensitivity and partial volume effects without loss of departmental working efficiency. The first step in testing risk-adapted radiotherapy in low risk patients is to confirm that women with low and high risk tumours can be reliably identified.

2.3 Low and high risk subgroups can be distinguished

Data from four prospective randomised trials testing radiotherapy after breast conservation surgery have reported on factors associated with local recurrence risk (8). The total number of patients randomised in these trials is 2,578, but the total number of local recurrence events is relatively small. The fifth (Scottish) trial of 585 patients under 70 years of age with clinical tumours < 4 cm did not attempt to identify risk factors for local-regional recurrence (9). More selected sources of information include published retrospective analyses of patients treated at single institutions.

Pathological tumour size > 2 cm was associated with a risk ratio for recurrence of 2.3 (95% CI 1.3 - 2.9, $p = 0.008$) in a Cox multivariate regression analysis of the Canadian trial, which randomised 837 histologically node negative patients with pathological tumour size < 4 cm (10). The Milan II trial reported a local recurrence rate after quadrantectomy alone of 12.6% for tumours > 1.6 cm compared with 6.7% for tumours ≤ 1.6 cm in 273 patients with tumours < 2.5 cm pathological diameter randomised to the surgery only arm (8). Note that in this trial, local recurrence was defined as malignancy appearing within 3 – 5 cm of the surgical scar line, more distant tumours being classified as new primaries. In a Cox regression analysis of the NSABP B-06 trial of 1,262 patients treated by tumorectomy +/- radiotherapy, maximum tumour size was associated with a higher risk of recurrence in node negative patients, but not in node positive patients (11). The Swedish trial of 381 women failed to confirm tumour size as a factor associated with increased risk of recurrence, but there were no tumours > 2 cm mammographic diameter in this study (12).

Lymph node status was not mentioned as a predictor of risk in a Cox regression analysis of the Canadian trial (10). Positive axillary lymph nodes were associated with a local recurrence risk of 12.1% compared to 7.2% for node negative patients in 273 patients randomised to quadrantectomy only in the Milan II trial, but no multivariate analyses was performed (8). In the NSABP B-06 trial, node positive patients had a significantly lower ipsilateral breast recurrence rate than node negative patients (8.8% versus 17% at 20 years), a difference attributed to the increased use of systemic therapies in the former subgroup (13, 14). Node status was not a significant negative predictor of local recurrence risk in an earlier multivariate analysis of this trial (11). The Swedish trial does not contribute, since it included only node negative patients (12).

Lymphovascular invasion is not reported in the Canadian, Milan II or NSABP B-06 trials. Lymphovascular invasion was not a risk factor for local recurrence in a multivariate analysis of the Swedish trial (12). In a retrospective analysis of 263 patients treated by breast conservation surgery and radiotherapy in Nottingham, UK, lymphovascular invasion (plus young age, positive node status and large tumour size) were reported as a significant risk factors for local recurrence in a multivariate analysis of 56 ipsilateral recurrences (15).

A multivariate Cox regression analysis of the Canadian trial reported a risk ratio for local recurrence of 1.5 (95% CI 1.0-2.3, $p = 0.04$) for poor nuclear grade (broadly equivalent to histological grade III) (10). Analysis of *tumour grade* is not reported in the Milan II trial. A risk ratio of 1.49 (95% CI 1.20 – 1.85) for high nuclear grade was reported in a multivariate analysis of the NSABP B-06 trial (11). In the Swedish trial, comedo ductal carcinoma (almost exclusively grade III) was associated with a 2.5-fold increase in local recurrence rate in a multivariate analysis (12).

The importance of *resection margins* is impossible to judge in any of the four randomised studies. The clearest demonstration of an effect is gained from the Milan III trial, which randomised 705 women to quadrantectomy (> 2 cm margin) or tumourectomy (≤ 1 cm margin) prior to radiotherapy (16) (17). There were 63 local recurrences in the group randomised to tumourectomy compared to 25 randomised to quadrantectomy, local recurrence being defined as tumours appearing within 3 – 5 cm of the resection scar (17). A selected retrospective analysis from Boston reported 5 year local recurrence rates (with 95% CI) among 885 patients with negative, close (< 1 mm margin), focally positive, more than focally positive margins as 0% (0 – 4), 4% (0 – 20), 6% (1 – 17) and 21% (10 – 37) (18).

The Canadian trial applied Cox regression analysis to identify patient age < 50 years as a risk for local recurrence, with a hazard ratio of 1.8 (95% CI 1.34-2.47, p = 0.001) after almost 8 years of follow up (10). The Milan II trial also reported young age as a risk factor for local recurrence, but did not examine this by multivariate analysis (8). The recurrence rates were 17.5%, 8.7% and 3.8% for women aged < 45, 45 - 55 and > 55 years, respectively, at a median follow-up of 39 months (range 28 to 54 months). When the three Milan trials were analysed together (n = 1,973), the effect of young age was still seen (19, 20). The NSABP B-06 trial reported age < 50 years as a significant predictor of local recurrence, using Cox multivariate regression, with a risk ratio of 1.29 (95% CI 1.05 – 1.60) (11). Multivariate analysis also identified age < 50 years as a significant risk factor for local recurrence in the Swedish trial, with 3% reduction in recurrence risk per year of increasing age (12). A further demonstration of the age effect is seen among 5,569 women randomised to boost therapy after breast conservation surgery and radiotherapy for early breast cancer, where young age (especially < 40 years) was a highly significant predictor of recurrence risk in a multivariate analysis (21).

Extensive intraductal carcinoma (EIC) was associated with a higher risk of local recurrence in the Milan II trial (8). However, subsequent analysis of 1,973 patients in all three Milan conservation trials reported EIC to be a significant risk factor only if the surgical resection margins were ‘narrow’ (not defined) or incomplete (19). EIC was not analysed in the Canadian or NSABP B-06 trials. EIC was found not to be a significant risk factor for local recurrence in a multivariate analysis of the Swedish trial that included age, tumour size, lymphovascular invasion and histopathological type (12). Among the most reliable retrospective data reported were those from Boston, where the adverse effect of EIC on local recurrence risk was reportedly neutralised by confirmation of complete microscopic margins (≥ 1 mm) at excision/re-excision (18). The use of EIC as a decision-making tool for breast conservation therapy is no longer recommended for routine practice. This is due to the lack of an internationally accepted definition of EIC, better pre-operative imaging to detect multifocal disease, and improved pathological assessment of surgical margins (personal communication: J Connolly and SJ Schnitt, Boston).

The significance of *histological tumour subtype* was not commented upon by the Canadian or Milan II trials. The NSABP B-06 trial reported papillary, tubular and mucinous subtypes to be associated with a significantly lower risk of local recurrence than more common histological subtypes in multivariate analysis (11). Invasive lobular carcinoma was associated with a 2.5-fold increased risk (95% CI 0.95-6.4, p = 0.06) of local recurrence in a multivariate analysis of the Swedish trial (12).

Recently, it has become apparent that a further subgroup of patients can be identified, with an extremely low risk of recurrence. This is largely due to better surgical management and increasing use of systemic therapies (chemotherapy and anti-oestrogen therapy). This became apparent following the first analysis of the NCRI START trial in 2007. This trial testing alternative radiotherapy regimens in 4451 UK women after breast conservation surgery reported local tumour relapse rates of 3.5% at 5 years. In a subgroup of node negative patients with grade 1-2 tumours with a size of 2 cm or less, the local relapse rate was 1.5% at 5 years. The absolute reduction in local recurrence from radiotherapy in this extremely good prognostic group, is clearly very small. It is important that modern partial breast radiotherapy trials do not limit eligibility to just the extremely low risk group, as there is a risk of an inadequate event rate (local recurrence) and the results will not be applicable

to patients at slightly higher risk of recurrence, but who still form part of a good prognostic group.

Taking the above factors into account, it is possible to identify a subpopulation with relatively low risk of ipsilateral breast recurrence, who are still likely to derive benefit form radiotherapy. This population includes patients with *all* of the following clinical-pathological features: age ≥ 50 years, pathological tumour size ≤ 3.0 cm pT1-2 (< 3.1 cm maximum microscopic diameter of invasive component), invasive adenocarcinoma (excluding invasive carcinoma of classical lobular type), unifocal disease, grade I - III, minimum microscopic margin of non-cancerous tissue ≥ 2 mm (excluding deep margin if this is at deep fascia) and negative or 1 - 3 positive axillary lymph nodes. At least 40% of all patients fall into this subgroup.

2.4 Whole breast radiotherapy is unnecessary in women with low risk disease (< 1% annual risk of local recurrence risk after surgery, systemic therapy and whole breast radiotherapy)

The case for partial breast radiotherapy after breast conservation surgery in low risk patients is based on the following: i) local recurrence risk is highest in the index quadrant, ii) new foci of breast disease outside the index quadrant are often new primary tumours (not influenced by radiotherapy) and iii) exposure of whole breast, underlying ribcage, heart and lung determines most of the iatrogenic morbidity. Before reviewing clinical data, the pathological data relating to the above points will be examined.

Serial sub-gross examination of 264 mastectomy specimens from patients with tumours ≤ 4 cm diameter revealed 40% cases with intraduct and/or invasive foci ≥ 2 cm from the microscopic edge of the index lesion (23). This rate fell to 11% at 4 cm distance from the tumour edge (7% invasive, 4% intraduct). Tumour foci ≥ 4 cm from the index lesion are likely to be located in different breast duct systems, since the 3D anatomy of the normal breast based on serial sub-gross sectioning shows the duct systems to be arranged in a regular radial array around the nipple (24). Although spread of neoplastic cells via anastamoses between duct systems cannot be excluded, it is reasonable to postulate that tumour foci > 4 cm from the primary tumour edge represent independent neoplastic or pre-neoplastic pathological entities, although this does not exclude clonality in relation to one or more genetic changes. This hypothesis is greatly strengthened by data from the results of autopsy on women not known to have breast cancer at the time of death. In a meta-analysis of seven series identified in a Medline search 1966-96, a total of 852 autopsies were submitted to breast examination ranging sub-gross examination, radiographic examination or sampling (25). With a mean number of breast sections in different series ranging from 9 to 275, the median prevalence of duct carcinoma in situ (DCIS) was 8.9% (0 – 14.7) and of invasive carcinoma was 1.3% (0 – 1.8). This prevalence is very similar to the rate of lesions ≥ 4 cm away from the primary lesion in patients with proven carcinoma quoted above (23).

Clinical observation of the spatial and temporal pattern of ipsilateral ‘relapse’ outside the index quadrant is entirely consistent with the majority of such lesions being independent tumours. The majority of relapses occur close to the primary site, and are assumed to be true recurrences. For example, in 2,544 patients treated by breast conservation surgery +/- radiotherapy at the National Cancer Institute, Milan between 1970-89, 142/191 (74%) ipsilateral relapses presented ≤ 2 cm from the surgical scar (26). Interestingly, the rate of ‘new primaries’ outside the index quadrant in the irradiated breast was comparable with the

rate of contralateral primary disease. In a separate study of 1,152 women treated by tumour excision and radiotherapy and followed up for a minimum of nine years (mean 14 years), ipsilateral relapses were classified as new primaries if they were of a different histological subtype, had a lower histological grade than the primary lesion or were diploid when the primary tumour had been aneuploid (27). On this basis, 60/136 were classified as true local recurrences, all of which had appeared by nine years of follow up. These patients had a subsequent 10-year survival of just over 50%. By contrast, 70/136 were classified as new primaries that continued to present over the whole 15-year period of follow up. This subgroup enjoyed a subsequent 10-year survival rate > 90%. In conclusion, the distinction between local recurrence (occurring predominantly in the vicinity of the index lesion and within 5 – 10 years of primary treatment) and new primary (occurring anywhere in the breast and at a constant annual rate throughout life) appears valid.

The implications of these data are that the tumour bed should be placed at the centre of the target volume as far as local anatomy allows, and that breast tissue beyond the index breast quadrant can be safely excluded. A breast quadrant is not a precise clinical or pathological entity. Experience of partial breast radiotherapy to date is reviewed in Appendix 1, page 36. In the context of the current proposal, partial breast radiotherapy refers to a clinical target volume centred on the tumour bed and extending to the proximal edge of the nipple, encompassing, on average, one-quarter to one-third of the whole breast. By reducing the target volume, lung and heart are totally excluded from the high dose zone, thereby eliminating the morbidity and mortality risks associated with exposure of these organs. Partial exclusion of rib-cage, pectoral and intercostals muscles and breast is expected to greatly reduce late adverse effects arising in the excluded tissues, since all tissues are expected to demonstrate some volume sparing effect (28).

2.5 A modest dose reduction outside the index quadrant will reduce late morbidity markedly without compromising tumour control

Traditional radiation dose-response relationships for tumour control are assumed to be sigmoid in shape, with a quasi-threshold dose below which treatment is ineffective. This is the basis on which current schedules are delivered, and it is consistent with the response of clinical disease. However, the dose-response relationships of subclinical disease are expected to be quite different. After breast conservation surgery, for example, < 50% patients will suffer a local recurrence if no radiotherapy is given. In these patients, the number of residual tumour clonogens could range from a theoretical minimum of 1 cell up to a maximum of 10^8 cells (the upper boundary of what constitutes subclinical disease in a patient with a microscopic complete excision). Assuming the distribution of residual tumour at the start of radiotherapy in a population of patients is log-linear, the radiotherapy dose-response is also expected to be log-linear, without a threshold (29). Clinical data derived from elective treatment of potential sites of spread from carcinomas of the head and neck, breast, ovary, cervix and lung, and from testis, soft tissue sarcoma and melanoma are consistent with a linear dose response relationship for the eradication of subclinical disease (30). On the basis of these empirical data and the patterns of tumour relapse data described above, it is predicted that a 10% reduction in dose intensity to tissues outside the index quadrant will have no measurable effect on local tumour control. The expected large impact on the risk of late normal tissue injuries will now be considered.

In contrast to the response of subclinical cancer, dose-response relationships for late normal tissue injuries are almost maximally steep as determined by Poisson statistics over the clinical range of curative dose. Whereas a 10% reduction in dose intensity is expected to

have no observable impact on eradication of subclinical tumour (see above), the incidence and severity of late normal tissue injuries are expected to fall significantly. In a recent randomised trial comparing 39.0 Gy and 42.9 Gy in 13 fractions over 5 weeks (equivalent to 46.7 Gy and 53.9 Gy in 2.0 Gy fractions assuming an α/β value of 3.0 Gy) to whole breast after complete local resection of primary tumour, there was more than a two-fold difference between regimens in the probability of changes in photographic breast appearance and of palpable breast induration at 5 years (31). These and other clinical data on the dose response of late effects are consistent with an expected halving of the incidence and severity of late adverse effects in response to a 10% reduction in dose intensity (32). If up to two-thirds of the breast volume and a higher proportion of non-target tissues inside the treatment volume are exposed to 10% less dose, the benefits in terms of reduced iatrogenic morbidity are expected to be highly clinically significant and readily detectable in a randomised trial. In those non-target (ribs and muscle) tissues exposed to an equivalent total dose less than 40 Gy in 20 fractions, very few late radiation sequelae are expected. In conclusion, a reduction in dose intensity outside the index quadrant is expected to have a major beneficial effect on iatrogenic morbidity without measurable detriment in terms of local tumour control.

2.6 Localisation of the tumour bed needs to be improved in all patients

Improved tumour localisation is long overdue for patients treated by breast conservation surgery and radiotherapy. Accurate localisation is essential for the delivery of partial breast radiotherapy for low risk tumours and of dose intensification for high risk tumours. This should involve the routine insertion of titanium clips or gold seeds to mark the excision cavity at the time of primary surgery for localisation using CT scans or simulator. These issues are reviewed in Appendix 4.

2.7 Full radiotherapy dose compensation should replace traditional techniques in all patients

Unplanned dose variations in excess of ICRU guidelines (+7%; -5%) are no longer necessary or acceptable in the breast (6, 7). Interim analysis of one prospective randomised trial (n = 306) suggests substantially less change in breast appearance two years after full dose compensation compared to standard wedge techniques (5, 33). Full dose (3D) compensation in the breast is now possible in most UK departments, and is recommended in the latest COG guidelines (34). These issues are discussed in detail in Appendix 5.

2.8 Intensity modulated radiotherapy to the breast exploits widely available technologies

Significant advances in radiotherapy technology have been made during the last ten years. Linear accelerators have the capability to deliver multiple segment ‘step and shoot’ fields, and dynamic beam deliveries, whilst treatment planning systems have sophisticated algorithms for both forward and inverse planning (35). Verification technology is similarly developing with amorphous silicon systems allowing high quality imaging at very low dose levels (36, 37). These have enabled the clinical application of IMRT in a small number of UK centres. In the interests of safety, The Royal College of Radiologists (UK) has recently recommended that intensity modulated radiotherapy should only be introduced in the context of well-designed prospective randomised trials (38). The proposed approach in IMPORT LOW is to use a small number of shaped segments and a set of asymmetric fields for the control and test arms, respectively. IMPORT LOW will encourage the use of dose modulation in the simplified setting of the tangential field irradiation of the breast and the full use of the technology now available to radiotherapy centres. Once developed and tested, the advanced technologies are likely to be more efficient and cost-effective than those in current use.

3. AIM

To test partial breast radiotherapy delivered using intensity modulated techniques following complete local tumour excision of low risk early stage breast cancer.

4. ELIGIBILITY

4.1 Inclusion criteria

***All* of the following:**

- Age ≥ 50 years.
- Primary breast conservation surgery +/- adjuvant systemic therapy
- Pathological tumour size ≤ 3.0 cm pT1-2 (< 3.1 cm maximum microscopic diameter of invasive component).
- Invasive adenocarcinoma (excluding invasive carcinoma of classical lobular type).
- Unifocal disease.
- Grade I, II or III
- Minimum microscopic margin of non-cancerous tissue ≥ 2 mm (excluding deep margin if this is at deep fascia).
- Lymphovascular invasion present or absent
- Axillary lymph nodes negative or 1 to 3 nodes positive (pN0 or pN+(1-3))
- No blood-borne metastases.

4.2 Exclusion criteria

- Previous malignancy (other than non-melanomatous skin cancer).
- Mastectomy.
- Invasive carcinoma of classical lobular type.
- Primary endocrine therapy or chemo-therapy (neo-adjuvant endocrine therapy is permissible as long as the tumour is <3.0 cm and all other inclusion criteria are met. Primary endocrine therapy as a replacement for surgery is not permissible)
- Concurrent chemo-radiotherapy

5. RANDOMISATION

Whole breast radiotherapy (without a boost) will be compared to two dose levels of partial breast radiotherapy after breast conservation surgery for early stage invasive breast cancer. Centres will give treatment based on 40 Gy in 15 fractions to the whole breast (control arm).

5.1 Treatment arms

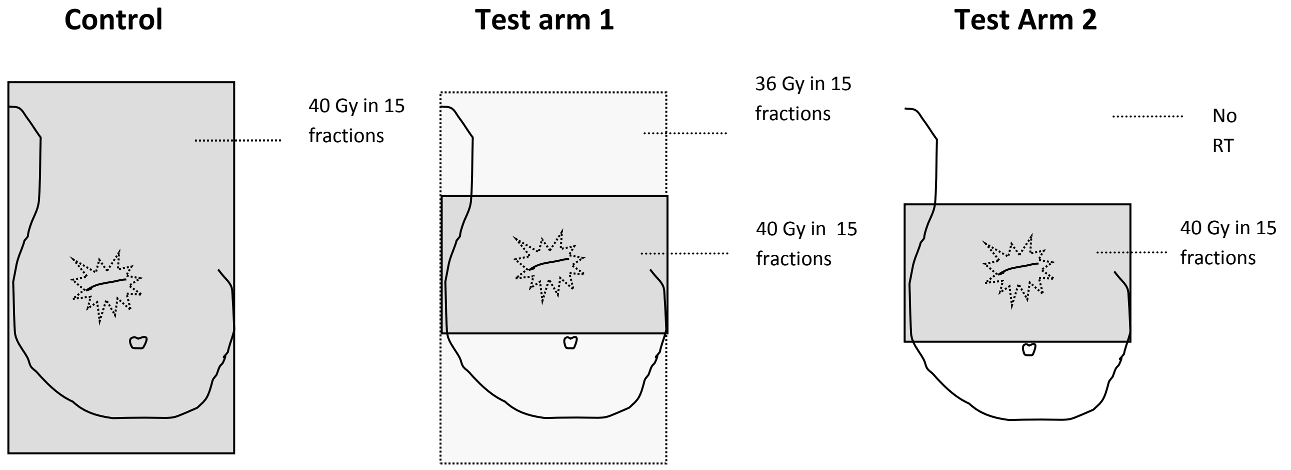

Equivalent total doses are presented in Appendix 2.

Full dose (3D) compensation is recommended for the control arm, even though many departments do not currently deliver this as part of standard treatment.

5.2 Entry procedure

Randomisation will be performed by telephone or facsimile (web-based randomisation may become available during the life of the trial). Treatment allocation will be 1:1:1, and will use computer-generated random permuted blocks. Randomisation will be stratified by centre only.

Patients will be allocated a unique randomisation number by telephoning the ICR-CTSU

020 8643 7150 (09.00 – 17.00 Monday – Friday)

6. RADIOTHERAPY TARGET VOLUMES, LOCALISATION AND OUTLINING

6.1 Target volume definition (see Appendix 3)

Partial breast CTV

The partial breast CTV is not a precise anatomical entity, but approximates to a quadrant of the breast. It is based on the pattern of residual disease reported in whole organ sections of mastectomy specimens. In practice, the tumour bed is firstly identified using the recommended imaging modalities. Using surgical clips, this would consist of the clips and change in surrounding tissue architecture. Using ultrasound or MRI, this would consist of the tumour cavity. A minimum CTV margin of 15 mm should be added and modified according to the individual breast anatomy. Radial limits must take account of surgical excision margins (particularly superior and inferior margins, since these determine field length). The posterior margin should not extend beyond the deep fascia (unless clearly breached by the tumour) and therefore, exclude the underlying muscle and ribs. If the anatomy of this region cannot be easily visualised, the posterior margin should not extend beyond 5 mm anterior to the lung/chest wall interface. The CTV should not extend radially beyond the edges of the visible/palpable breast. The anterior extent of the CTV should be

limited to 5 mm below the skin surface to reduce late normal tissue skin changes. The actual CTV around the tumour bed should approximate to the volume of a breast quadrant and therefore reflect the conceptual CTV margin around the tumour.

Whole Breast Clinical Target Volume (CTV)
This is based on the recommendations from the START trial (39). The CTV includes the soft tissues of the whole breast down to the deep fascia, excluding muscle and underlying rib cage.

Planning Target Volumes (PTV)
A margin should be added to each CTV, taking into account set-up error, breast swelling and breathing. A typical PTV margin is 10 mm. The PTV should stop 5 mm beneath the skin surface in order to preserve skin sparing.

Radiotherapy field boundaries
The field boundaries should cover the PTV with a sufficient margin for beam penumbra. This would typically be of the region of 5 mm, though in the anterior direction the field border should be 15 – 20 mm beyond the skin surface to allow for breast swelling.

The field arrangement for the partial breast radiotherapy will consist of ‘mini-tangents’. Therefore, the anterior and posterior dimensions of partial breast fields are likely to be the same as the whole breast fields. The field length in the superior-inferior direction, however, will be shortened. For example, field lengths will be determined by localising the tumour bed, adding 15 mm CTV margin (or less if the superior or inferior limits of breast tissue are reached), 10 mm PTV margin, and 5 mm for beam penumbra, to above and below the tumour bed. The fields will often be 80 –100 mm in length.

Organs At Risk (OAR)
Organs at risk (OAR) include the heart, lung and chest wall. Localisation of OAR is limited by the quality of the imaging modality. Ideally, dose-volume histograms should be created for the heart and lung, but this would require knowledge of the entire volume of these organs. For those centres using a limited number of CT slices for planning, or using a non-CT method e.g. optical outlining, a record of heart and lung depth from the simulator films or beams eye views of standard tangential fields could be used to give an estimation of the amount of internal organ irradiated.

6.2 Tumour bed localisation (see Appendix 4)

General points
The patient must be scanned in the radiotherapy treatment position, whatever the imaging modalities used. Each centre must develop its own localisation protocol, and have it approved by the QA team. If it is initially impossible to adopt one of the recommended procedures (surgical clips/gold seeds, CT scanning, ultrasound or MRI, see below), it is permissible to enter patients if the clinician is confident that localisation is accurate in a particular case. For example, if an obvious tissue deficit is palpable in the treatment position, or a combination of mammographic localisation, pre-operative assessment/photographs and surgical diagrams offer consistent information on the primary site, patients may be entered into the trial. The method of tumour localisation will be recorded for each patient.

Titanium surgical clips or gold seeds
These are the recommended techniques for localising the partial breast volume. The technique is simple, inexpensive and quick, and offers benefits for patients requiring tumour localisation regardless of trial entry. It should be discussed and agreed, if possible, with all referring surgeons before activating the trial. Medial, lateral, superior, inferior, and posterior (deep) cavity edges are marked using pairs of titanium clips/gold seeds at the time of surgery. A sixth anterior pair of clips may also be placed under the skin and subcutaneous tissue. Surgical clips/gold seeds can be localised using a conventional simulator (orthogonal films), a CT-simulator or CT scanner.

Ultrasound
A combination of 2D ultrasound and CT scanning is a reliable alternative to visualise the tumour cavity without the use of surgical clips.

Magnetic Resonance (MR) imaging
MR imaging can be used to obtain anatomical information of the tumour cavity and surrounding tissues without the use of surgical clips, CT scanning or ultrasound. Ideally, this should be available electronically in the radiotherapy planning system.

6.3 Patient position

The patient must lie supine, and the position must remain unchanged during outlining, planning and treatment. An immobilisation device, such as a breast board with arm and wrist supports, an arm pole and/or vac-fix bag should be used. Ideally, the immobilisation should allow daily reproducibility of +/- 5 mm.

6.4 Outlining

A facility for taking multiple outlines is OBLIGATORY. If possible, a full 3D set of outlines for the whole breast should be collected. The imaging technology to be used includes x-ray CT, optical (40-42) simulator with CT attachment, or MR. Where optical systems possess a lung fitting algorithm, this should be used (43). As a number of centres are unlikely to have easy access to equipment enabling a full 3D outline set to be acquired, Table 1 suggests the minimum data necessary for each arm of the trial.

Trial Arm	Minimum outline data required
Control arm	Outline through centre of breast volume plus either (a) a representative slice superior and one inferior to central plane for standard wedged pair (Minimum of 3 outlines) or (b) at least 4 - 6 further slices spaced through the breast volume (and outside the field borders at the superior and inferior edges) for a compensated/IMRT treatment. Minimum of 5 - 7 outlines (11, 44, 45).
Test arm 1	One outline through centre of the partial volume, 1 at the level of the nipple and 1 through the centre of the whole breast volume plus a minimum of 1 further representative outline superior and 1 inferior to central plane. Minimum 5 outlines.
Test arm 2	One outline through centre of the partial volume and one at the level of the nipple. Minimum of five outlines.

Table 1: Minimum data requirements for patient contour acquisition.

7. RADIOTHERAPY PLANNING

7.1 Control arm

The control arm whole breast geometry is a tangential pair incorporating a non-divergent posterior field border on the tangential fields. The treatment plan should aim to fulfil the criteria of ICRU 50 and 62 (6, 7) i.e. the breast PTV should receive no less than 95% or more than 107% of the prescribed dose. This is the ideal situation. In practice the volume of breast PTV which receives dose outside of these limits should be reduced as much as possible (this volume to be less than 10% and close to 5% of the total volume should be achievable for the majority of patients (5, 13, 14). A standard wedged pair will be sufficient for some patients but others will require compensation. A list of simple compensation methods is given in Table 2 with references and comments. Appendix 5 gives a suggested general, manual method for deriving one or two segments for an IMRT plan based on a standard wedged pair.

Method	References	Comments
Physical compensators based on equivalent path length	(45)	Osiris system (QADOS) commercially available - would require the isodose planning module.
Simple manual segments	(46, 47)	Straightforward and can be achieved with CT or other outlining methods as long as the minimum slice set of Table 1 is obtained.
Commercial planning systems plane compensation algorithms	Varian Cadplan<br>MDS Nordion Helax<br>Adac Pinnacle<br>Nucleotron Plato/OPT	Available on many planning systems (combining with a wedge on one field may improve dosimetry further)
Inverse planning algorithms on commercial planning systems	Varian Cadplan/Helios<br>Nucleotron OPT<br>Adac Pinnacle	This is an alternative if the plans meet the ICRU 50 and 62 criteria
Electronic portal imaging	(48)	Expertise exists and could be made available to implement this method with any portal imager with good dosimetric stability and accuracy.
Other techniques	As applicable	An alternative if the plans meet the ICRU 50 and 62 criteria

Table 2: Treatment planning methods for compensation

7.2 Test arm 1 Geometry

The geometry consists of a basic tangential pair as described for the control arm. It is suggested that such plans are generated using a single isocentre placed in the centre of the partial breast quadrant. Asymmetric jaws may then be used to deliver whole breast fields. The whole breast fields should be planned first and the criteria of ICRU 50 and 62 applied to assess whether compensation will be required to keep the dose sufficiently homogeneous. The quadrant fields are then added. Their weights can easily be calculated knowing the contributions from the whole breast fields to the isocentre. Dose homogeneity within the quadrant should be within the ICRU 50 and 62 criteria.

7.3 **Test arm 2 Geometry**

As for the whole breast control arm, but with the field sizes considerably reduced so as to encompass the quadrant containing the tumour bed only.

7.4 **Suggested technique for field definition using a simulator: IMPORT LOW test arms**

With the patient in the treatment position, decide on the superior and inferior limits of the partial volume. Derive the central plane of this volume and mark it. This plane will contain the treatment isocentre, and asymmetric jaws may be required to cover the whole breast volume. Delimit the four margins of the whole breast according to local approved practice. Mark the superior and inferior limits of the quadrant with wire when taking the planning radiograph for the whole breast treatment.

7.5 **Inhomogeneity correction**

Lung density correction should be used. For centres not using CT scanning of patients, methods for approximating lung density corrections should be discussed with the QA team.

7.6 **Dose Prescription**

Doses are prescribed as described in the table below. The reference point is halfway between the lung and skin surfaces in the transverse plane, and centrally in the medial-lateral direction for the relevant volume

Trial arm	Prescription Dose (Gy)	Dose /Fraction (Gy)	Reference point	Prescription isodose (%)	Coverage isodose (%)
Control (Whole breast)	40.0	2.67	Centre of whole breast	100.0	95.0
Test arm 1 Partial breast	40.0	2.67	Centre of partial breast	100.0	95.0
Test arm 1 Whole breast	36.0	2.4		90.0	86.0
Test arm 2 Partial breast	40.0	2.67	Centre of partial breast	100.0	95.0

7.7 **Organ at risk sparing: cardiac shielding**

This may be carried out according to local practice, using MLC or shielding blocks. The same policy would be used for all arms of the trial.

7.8 **Bolus to scar**

No bolus should be applied to the skin, including excision scar.

7.9 **Beam energy**

Beam energies for treatment as for local practice, 4 - 6 MV. If a higher energy is required for some patients, this may be discussed with the IMPORT LOW QA team.

8. RADIOTHERAPY VERIFICATION (see Appendix 6)

A simulated verification field should be used for patient set-up (i.e. use of a non-treatment image). The verification field will cover the whole breast and extend to stable midline and lateral points. This will allow daily light field verification (as occurs at present) irrespective of trial arm or tumour location. Practically, the isocentre would be established and the pre-determined verification field used to check alignment with medial and lateral tattoos (see Figure 1 below). The field border positions for the treatment fields would be related to the stable tattoos.

The same verification field would be used for portal verification. This would include reference anatomy, and would provide the same verification irrespective of trial arm or tumour location. The treated area could be highlighted by double exposure if desired.

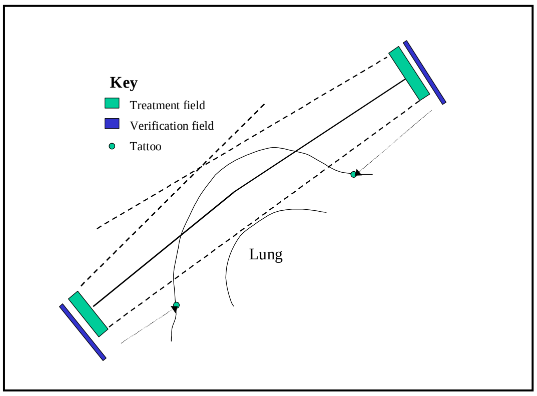

Figure 1: A schematic view of the verification field showing the alignment with tattoos.

Portal verification should be weekly to give confidence that the tumour bed/quadrant area has not fallen outside the treated area. In addition we recommend taking of at least three images in the first week to determine and allow correction for any systemic error.

Dosimetric and planning data will be collected centrally and analysed using clinical trials analysis software. Data to be collected includes CT slices, outlines, beam parameters and DVH data, if available.

9. RADIOTHERAPY QUALITY ASSURANCE

A comprehensive quality assurance programme is planned for all centres involved with IMPORT LOW (see Appendix 7). In line with current UK guidelines, all patients should have in-vivo dosimetry within the first week of treatment. This may be performed using diodes or thermo-luminescent dosimetry (TLD). Other methods may be appropriate for individual centres and should be discussed with the QA team. The verification method must be independent of the planning system. Verification of patient positioning should be performed in line with protocol recommendations.

If a patient's treatment is extended by more than 5 days due to lack of machine availability (breakdowns or scheduled service days) or bank holidays they should be classified as category 1 for the remainder of their treatment. No differentiation between patients in alternative treatment arms should arise when considering rescheduling patients following breakdown or bank holiday.

10. FOLLOW-UP

All patients will receive daily radiotherapy for three weeks and will be followed up annually for 10 years.

11. ENDPOINTS

The primary endpoint is local tumour control in the ipsilateral breast. Secondary endpoints include location of tumour relapse, contralateral primary tumours, regional and distant metastases, late adverse effects in normal tissues, quality of life (QL) and cost-effectiveness.

11.1 Tumour-related endpoints

Ipsilateral tumour relapse and contralateral primary tumour must be confirmed by cytological/histological assessment. Metastases will be determined by an appropriate combination of clinical, haematological, imaging and pathological assessment, recognising that pathological confirmation is not always possible.

It is vital to establish the location of any ipsilateral tumour relapses and compare these with the original radiotherapy volumes for each individual patient. The management of recurrences/new primaries varies from centre to centre. Also, it is important that the mapping techniques are practical to enable the maximum value from the information gathered. For this reason some suggested recurrence mapping recommendations have been devised with a variety of complexity. This takes into account that technology would be rapidly improving over the lifetime of the trial. The patient information sheets and case report forms will be used to alert the patient and health care professionals of the IMPORT trial if a recurrence/new primary occurs. Recommendations for recurrence/new primary mapping are given in Appendix 11.

Ipsilateral tumour relapse (true recurrence plus new primary tumour)
Defined as:
Control arm: Breast parenchyma/skin within volume receiving 40 Gy in 15 fractions.

Test arm 1: Breast parenchyma/skin within volume receiving 40 Gy in 15 fractions
Breast parenchyma/skin outside volume receiving 40 Gy in 15 fractions but within volume receiving 36 Gy in 15 fractions.

Test arm 2: Breast parenchyma/skin within volume receiving 40 Gy in 15 fractions
Breast parenchyma/skin outside volume receiving 40 Gy in 15 fractions, but within the whole breast volume included in the Control arm.

Please refer to schema in section 5.1

Other second primary cancers
Contralateral primary breast cancer.
Other primary cancer.

Overall survival & disease-free survival
Regional metastases (axilla, supraclavicular fossa, internal mammary chain).
Haematogenous metastases (only details of the first relapse are required).
Death.

These events will be recorded, but do not constitute primary endpoints.

11.2 Treatment-related endpoints

Late adverse effects will be measured using a combination of clinical and photographic assessments and patient self-assessments. Digital photographic assessments will be performed at baseline, 2 & 5 years as in the NCRI START Trial, since change in photographic breast appearance is sensitive to 10% difference in randomised dose. Photographic assessments will be supplemented by physician assessments of the breasts at years 1, 2, 5 and 10 and outcome will be correlated at years 2 and 5. Patient self-assessments for quality of life will include the EORTC QLQ C-30 core questionnaire, the breast cancer module BR-23, Body Image Scale, Hospital Anxiety and Depression Scale and the EuroQol (EQ)-5D at baseline, 6 months, 1, 2 and 5 years.

Physician assessments of late adverse effects (in all centres)
At years 1, 2, 5 and 10, physicians will record the development of breast shrinkage/distortion, breast induration, telangiectasia and breast oedema. Any other severe late side effects will also be recorded, such as ischaemic heart disease, rib fracture, symptomatic lung fibrosis, persistent cough, and including any specialist referral for investigation or management of late toxicity. Physician assessments will also be correlated with the results of the blood sample analysis investigation into molecular correlates of normal tissue injury (section 18.1)

Photographic assessments of late adverse effects (in a subset of centres with local facilities)
Photographic assessments after breast conserving surgery will be taken at baseline, and at years 2 and 5. Timing of assessments is based on experience from the START trial, with the aim of maximising the information collected whilst minimising the assessment burden. Two frontal views of the chest will be taken, one with hands on the hips and the other with hands raised as far as possible above the head. Both photographs will exclude the head.

Change of breast appearance compared with the post-surgical baseline will be scored on a three-point graded scale (no change, some change, marked change), together with an assessment of breast size and surgical deficit. All photographs will be taken and stored locally in the first instance. Periodically, all photographs will be collected and assessed blind by a select group of observers. Reliability and repeatability of the assessments will be verified. The feasibility of and procedures for this scoring mechanism have been established in the START trial and assessment will continue using existing criteria.

Patient self-assessments and quality of life (in a subset of centres)
Patient self-assessment questionnaires will be used to compare the radiotherapy schedules in terms of changes in body image, breast pain, arm swelling, shoulder movement, satisfaction with treatment outcome, sexual and psychological functioning and impact on daily living (see Appendix 9). The baseline questionnaires will be explained and administered to the patient in the clinic prior to randomisation. Follow-up questionnaires will be organised and sent out from the IMPORT LOW Trials Office at 6 months, 1 year, 2 years and 5 years. At the same time points, the EQ-5D preference-based measure of health status will be used as a basis for estimating quality-adjusted life-years for the purpose of the economic evaluation. All patients from a subset of centres will participate in this assessment (see Appendix 10).

11.3 Serious Adverse Events

(See Appendix 8)

All Serious Adverse Events (SAEs) must be reported within 24 hours using the specified SAE form. The form must be sent by FAX to the Institute of Cancer Research Clinical Trials and Statistics Unit on 020 8722 4368. These must be completed, signed and dated by the Principal Investigator or nominated person identified on the site delegation log. A hard copy must also be sent by post to the trials office using the address on the SAE form. The Chief Investigator or delegate will review all SAEs to assess the 'expectedness' of the event.

SAEs will be collected during the patient's radiotherapy treatment only. Patients showing unexpectedly severe early or late normal tissue responses will be identified on the Follow-up Forms. Reactions include unexpectedly severe late subcutaneous fibrosis, ischaemic heart disease (after both right- and left-sided radiotherapy), rib fracture, symptomatic lung fibrosis and other (specified) instances. Principal Investigators are asked to inform the IMPORT Trials Office within 8 weeks of any patient presenting with sensori-motor symptoms in the ipsilateral upper limb, regardless of aetiology.

12. ANALYSIS

12.1 Choice of principal endpoints

Several endpoints are being investigated, including local tumour recurrence, normal tissue effects, quality of life (QL). It is intended that each will be analysed separately. If there is discordance between the endpoints in terms of treatment outcome this will allow discussion of clinical trade-offs.

12.2 Methods of analysis

Survival analysis methods (i.e. Kaplan-Meier analysis and Cox proportional hazards regression) will be used to compare rates of local recurrence between allocated treatments for all randomised patients (i.e. intention to treat). Normal tissue effects will be analysed using methodology developed for START. The photographic assessments of breast appearance are expected to clearly discriminate between test and control arms of the trial, with significantly less breast shrinkage and distortion in test arms compared to control. The impact on QL of expected differences in the rates of change in breast appearance between treatment arms will also be investigated. It is likely that clinically relevant differences in QL parameters that cannot be inferred from photographic changes will be detected between treatment arms. Improvement in symptom status is expected in the test arms due to

protection of the pectoralis muscle and underlying rib-cage from high doses delivered to patients in the control arm. Analysis of the QL data will follow algorithms developed for the QL forms (i.e. calculation of standardised sub-scale scores), and will compare treatment groups at individual time points, as well as longitudinal changes from baseline. Repeated measures analysis of variance will be used to describe the longitudinal data. The QL analysis will take into account important prognostic factors such as age, stage of disease, treatment received and other socio-demographic and clinical characteristics using generalised linear models. Appropriate adjustments will be made for multiple comparisons in the analysis, by adopting a more stringent cut-off for statistical significance, e.g. $\alpha = 0.01$.

The sample size calculations have been based on survival analysis methods. The 5-year figure has been used as the clinically relevant time point and assumes that recurrences before and after five years will be included in the analysis accordingly (i.e. patients will be followed from randomisation until it becomes impractical to do so further, and patients will only be censored in the analysis upon death or if lost to follow-up). Analyses will incorporate the time to an event as well as the occurrence of that event.

The incidence of uncommon serious complications will be monitored.

Analyses of local tumour recurrence and of normal tissue effects will be performed, adjusting for adjuvant therapy (chemotherapy, hormonal therapy). Analyses of normal tissue effects will also be adjusted for breast size and surgical deficit.

Analyses will estimate the size of treatment effect with a confidence interval for the estimated difference between schedules. Information will be provided on both the absolute and relative treatment effect. Each test arm will be compared with the control arm and treatment effects estimated separately.

A prospective evaluation of the health economic consequences is planned in collaboration with Mark Sculpher, Centre for Health Economics, University of York (see Appendix 10). The use of less intensive radiotherapy is expected to reduce costs (in terms of the treatment and the management of side effects). If a higher recurrence rate were observed for the test arms this would have implications for both costs and health outcomes.

Depending on the results, a trade-off may need to be considered by patients and clinicians, taking into account health economic considerations. If tumour control is equivalent in all treatment arms, and radiotherapy adverse effects are milder in both test arms, interpretation is easy – test arm 2 becomes the treatment of choice. If there is an excess of ipsilateral tumour relapses in test arm 2 that are not detected in test arm 1, the latter will become the treatment of choice if the radiotherapy adverse effects are lower, as expected. A difficulty may arise if there is an excess of ipsilateral tumour recurrences in both test arms associated with fewer radiotherapy adverse effects. Quality of life and health economic considerations will inform policies that take into account differences in cost between managing excess local cancer recurrence in a tiny minority of women and late radiotherapy morbidity in a significant minority. Assuming, for the sake of argument, that the comparison is cost-neutral, patients and doctors will make informed choices based on the tumour control and QL data collected in the trial.

12.3 **Sample size**

Assuming a 2.5% recurrence rate at 5 years in the control arm, 613 patients per randomised arm would provide 80% power to exclude a difference of > 2.5% in either test arm (α = 0.025; 1-sided as testing for non-inferiority, and allowing for the 1:1:1 randomisation). Allowing for a 5% rate of loss to follow-up by 5 years, including loss due to disease progression, (based on experience in the START trial) gives 645 required in each arm, and the trial will therefore recruit a total of 1,935 patients.

Photographic assessments will only be required in a subset of the patients. Based on the results of the RMH IMRT trial, 400 patients per arm will have > 90% power to detect differences of ≥ 10% for change in breast appearance (2-sided α = 0.025, allowing for the 1:1:1 randomisation). For the quality of life study, 400 patients per arm will provide > 80% power to detect differences of ≥ 5 between the means of QL subscales (assuming a standard deviation of 20), or to detect differences of ≥ 15% in the prevalence of specific normal tissue effects (e.g. lymphoedema, shoulder stiffness, breast pain) and anxiety and depression. Sample size estimate for the quality of life study assumes a 2-sided significance level of α = 0.005 (to allow for multiple testing and the 1:1:1 randomisation) and allowing for 10% attrition due to illness or death (based on experience in the START trial). Therefore 1,200 patients will be entered into each of the photographic assessment and quality of life studies in total.

12.4 **Interim analyses and data monitoring**

Interim analyses of local tumour control, normal tissue responses, radiotherapy side effects and other endpoints will be conducted at yearly intervals and presented to an independent Data Monitoring and Ethics Committee (DMEC) for confidential review.

In the light of the interim analyses, the DMEC will advise the Steering Committee if, in their view, the trial has indicated "proof beyond reasonable doubt" that one of the schedules is clearly indicated or contraindicated in terms of local tumour control and late radiation effects. In reviewing the evidence, the DMEC will also consider any available data from other randomised trials involving similar comparisons. The Steering Committee may then consider modification or termination of the study. Unless such a situation arises, the Steering Committee, the Trial Management Group, the collaborators and the central administrative staff (except the statistician who prepares the analyses) will remain unaware of the interim results. The DMEC may recommend continuation beyond the planned number of patients in the main trial, the Quality of Life study or in the number of patients having photographic assessments, if it is felt that further information is required to address reliably the hypothesis in question.

12.5 **Publication & Presentation**

The success of the trial depends entirely on the participation by a large group of clinical oncologists. The main results of the trial will therefore be published in the name of the trial on behalf of all collaborators. All participants will be listed under the name of their hospital, together with the total number of patients entered.

13 Research Governance

13.1 Trial Administration and Logistics

The Institute of Cancer Research (ICR) is the agreed Sponsor of this study in accordance with the Research Governance Framework for Health and Social Care and GCP.

13.1.1 Chief Investigator

- The Chief Investigator is Professor John Yarnold.

13.1.2 ICR-CTSU responsibilities

ICR-CTSU has overall responsibility for facilitating and coordinating the conduct of the trial and is also responsible for collating data obtained, and undertaking and reporting interim and final analyses.

13.1.3 Participating centres responsibilities

Centres wishing to recruit to this study will be asked to provide evidence that they can deliver protocol treatment. This will include the successful completion of the IMPORT QA programme.

Responsibilities are defined in an agreement between an individual participating centre and The Institute of Cancer Research.

13.2 Investigator training

Prior to commencing trial recruitment, training will be provided to identified key individuals in each participating network by the Chief Investigator. Training will include discussion on the background to the study, evidence for partial breast radiotherapy and discussion on the issues of clinical equipoise. Experience developed from successfully recruiting centres and information from associated qualitative studies will be provided to participants at their initial training and subsequently on a regular basis. Participating centres will be asked to maintain a screening log. Randomisation acceptance rates will be monitored and additional support/training offered when lower than anticipated rates are encountered.

13.3 Case Report Forms

Case Report Forms (CRFs) which are in the form of a booklet should be completed for all patients and should not be made available to third parties.

CRFs should be completed as indicated in the Trial Guidance notes. CRFs are in duplicate. The completed top copy must be sent by the hospital to ICR-CTSU as soon as they are due. The bottom copy must be retained in the booklet and held by the investigator. If information is not known it must be clearly stated.

The Trial Management Group reserves the right to amend or add to the CRFs as appropriate. Such changes do not constitute a protocol amendment, and revised or additional forms should be used by centres with immediate effect. Where appropriate data may need to be collected retrospectively if an addition has been made to the CRF.

13.4 Protocol compliance/on site Monitoring

The IMPORT LOW trial is being conducted in accordance with the professional and regulatory standards required for non-commercial research in the NHS under the Research Governance Framework for Health and Social Care and GCP.

Participating centres may be monitored by ICR-CTSU and possibly by Health Authorities to carry out source data verification, and confirm compliance with the protocol. By participating in the IMPORT LOW trial the Principal Investigators at each centre are confirming agreement with his/her local NHS Trust to ensure that:

- Sufficient data is recorded for all participating patients to enable accurate linkage between hospital records and CRFs;
- Source data and all trial related documentation are accurate, complete, maintained and accessible for monitoring and audit visits;
- All staff at their centre who are involved with the trial are trained appropriately
- All original Consent Forms should be dated and signed by the patient, the person taking consent (if different to the researcher) and the researcher (the principal investigator at that site), and kept in a central log together with a copy of the specific patient information sheet(s) they were given at the time of consent.
- Copies of CRFs are retained for 20 years to comply with international regulatory requirements;
- Staff will comply with the trial guidance notes for the IMPORT trial.

ICR-CTSU will monitor receipt of CRFs. They will also check incoming CRFs for compliance with the protocol, inconsistent and missing data.

ICR-CTSU will contact centres to discuss dates of any proposed monitoring visits. Once a date has been confirmed a list of names of patients whose notes will be monitored during the visit will be sent to the centre. This list will be sent out in advance to give sufficient time for the notes to be made available. Site monitoring will usually be conducted at participating centres at least once during the first year following entry of the first patient. It is likely that a random sample of notes will be selected for limited source document verification.

13.5 Trial Management

13.5.1 Trial Management Group

A Trial Management Group (TMG) will be set up and will include the Chief Investigator (Professor John Yarnold) and the Chief Clinical Co-ordinator (Dr Charlotte Coles), co-investigators and identified collaborators, the trial statistician and the trial co-ordinators. Principal investigators and key study personnel will be invited to join the TMG as appropriate to ensure representation from a range of centres and professional groups.

Notwithstanding the legal obligations of the Sponsor and Chief Investigator, the TMG has operational responsibility for the conduct of the trial.

13.5.2 Trial Steering Committee

A Trial Steering Committee (TSC) will monitor and supervise the progress of the trial. The role of the TSC is to provide overall supervision of the trial on behalf of the funding body. In particular, the TSC will concentrate on the progress of the trial, adherence to the protocol, patient safety and the consideration of new information. Day-to day management of the trial is the responsibility of the Chief Investigator and TMG.

Membership will be limited and include an independent Chairman (not involved directly in the trial other than as a member of the TSC), not less than two other independent members, the Chief Investigator and the trial statistician.

Where possible membership will include a lay/consumer representative. Trial co-ordinators and other key members of the TMG will attend meetings (as observers) as appropriate. Observers from the funding body and, if applicable, host Institutions or sponsors will be invited to all meetings. The TSC will meet at least annually.

13.5.3 Data Monitoring and Ethics Committee

An independent Data Monitoring and Ethics Committee (DMEC) will be established to oversee the safety and interim efficacy of the trial. This committee will be constituted with guidance from MRC Good Clinical Practice (MRC GCP). The DMEC will meet on a regular basis as they see fit, but no less than annually. Following each meeting, the DMEC will report their findings and recommendations to the TSC and to the TMG.

13.6 End of Study

For the purposes of ethics approval, the study end date is deemed to be the date of the last data capture.

13.7 Archiving

Essential documents are documents that individually and collectively permit evaluation of the conduct of the trial and the quality of the data produced, for example CRFs, patient consent forms. These will be maintained at ICR-CTSU and at the Investigator Sites in a way that will facilitate the management of the trial, audit and inspection. They will be retained for a sufficient period (at least 20 years) for possible audit and inspection by the regulatory authority. The sponsor or trial organisers will notify the investigator sites of their responsibility for archiving essential documents. Documents will be securely stored and access will be restricted to authorised personnel. An archive log will be maintained to track archived documents

13.8 Publishing policy

All publications and presentations relating to the trial will be authorised by the TMG. A Writing Committee may be appointed. Authorship will be determined by the TMG and will include the Chief Investigator, co-investigators, trial co-ordinators and trial statisticians. Further authorship will be determined by centre accrual. All participating centres will be acknowledged in the final manuscript according to patient accrual.

14 Confidentiality and Liability

14.1 Risk assessment

This study has been formally assessed for clinical risk using a generic risk assessment matrix.

14.2 Liability/Indemnity/Insurance

This study is an investigator-led trial endorsed by the Clinical Trials Awards and Advisory Committee (CTAAC) of Cancer Research UK. Indemnity for participating hospitals is provided by the usual NHS indemnity arrangements.

14.3 Patient Confidentiality

Patients will provide their full name, date of birth, hospital number and NHS number at randomisation to allow tracing through national records. The personal data recorded on all documents will be regarded as confidential, and to preserve each subject's anonymity, only their initials and date of birth and Trial number will be recorded on subsequent Case Report Forms. Patients consenting to the Quality of Life study will provide their name, address and telephone number and also address and phone number of their GP. These details will only be used for the purposes of the Quality of Life study. The investigator must keep a separate log of patients' trial numbers, names, and hospital numbers. The investigator must maintain in strict confidence trial documents, which are to be held in the local hospital (e.g. patients' written consent forms). The investigator must ensure the patient's confidentiality is maintained.

ICR-CTSU will maintain the confidentiality of all subject data and will not reproduce or disclose any information by which subjects could be identified, other than reporting of serious adverse events. Representatives of the trial team will be required to have access to patient notes for quality assurance purposes but patients should be reassured that their confidentiality will be respected at all times. In the case of special problems and/or competent authority queries, it is also necessary to have access to the complete study records, provided that patient confidentiality is protected.

15 Ethical Considerations

It is the responsibility of the Chief Investigator to obtain a favourable ethical opinion (main REC approval).

It is the responsibility of the Principal Investigator at each participating Trust to obtain site-specific approval of the trial protocol and any subsequent amendments. All correspondence with the local REC should be filed by the Investigator.

It is the responsibility of the investigator to give each patient, prior to inclusion in the trial, full and adequate verbal and written information regarding the objective and procedures of the trial and the possible risks involved. Patients must be informed about their right to withdraw from the trial at any time. Written patient information must be given to each patient before enrolment. The written patient information is an approved patient information sheet according to national guidelines. This outlines the Quality of Life study, and the collection of biological specimens. Patients will be encouraged to participate in these associated studies but if they decline, this will not exclude them from the main trial.

It is the responsibility of the Principal Investigator to obtain signed informed consent from all patients prior to inclusion in the trial.

16 Withdrawal of patients from study treatment

Patients who do not receive their allocated treatment for any reason should be treated at the discretion of their clinician. However, analyses of all outcome data will be on the basis of intention to treat. Unless the patient requests otherwise, all CRFs, including long term follow up, should be completed, regardless of treatment actually received. A trial deviation form should be completed to record details of deviation from treatment allocation, and also for any patient who withdraws consent for further follow up. Patients are asked prior to randomisation to consent to follow up should they withdraw from the treatment allocation (see patient information sheet and consent form), and any patient unwilling to give that assurance prior to trial entry should not be randomised. Patients are however free to reverse that decision at any time without giving a reason.

17 Financial Matters

The trial is investigator designed and led, and has been approved by the Clinical Trials Awards and Advisory Committee (CTAAC). It is endorsed by Cancer Research UK and meets the criteria for R&D support as outlined in the Statement of Partnership on Non-Commercial R&D in the NHS in England.

Research costs (to ICR-CTSU) are being funded by Cancer Research UK. If additional financial support is received from any other source, this will be made apparent to the approving Main REC and CTAAC, but will not require a protocol amendment.

No individual per patient payment will be made to trusts or investigators, but NCRN (or regional equivalent) network resources should be made available as the trial is part of the NCRI portfolio by virtue of its approval by CTAAC.

18. ASSOCIATED STUDIES

18.1 Molecular correlates of normal tissue injury

It is thought that part of the inter-patient variation in the incidence and severity of late normal tissue responses to radiotherapy reflect inter-patient differences in the expression of specific proteins (growth factors, extracellular matrix components etc). Common DNA sequence variations (single nucleotide polymorphisms) within the controlling regions or coding sequences of genes account for differences in protein expression between individuals that may explain an important component of the variation between individuals in late normal tissue responses to radiotherapy. Genome-wide approaches offer scope to identify patterns of single nucleotide polymorphisms that distinguish patients at lower and higher than average annual risk of late adverse effects.

Up to twenty ml of whole blood will be collected by venesection into blood tubes and sent to the Cancer Research UK/MRC Tissue Bank at Ninewells Hospital, Dundee, where it will be stored for future research. The research may be carried out at another centre. An aliquot of this blood may also be requested for comparison of genomic DNA with tumour DNA extracted from donated tissue samples (see 18.2).

Blood will be collected at the treating hospital or if this is not possible, at the patient’s health centre. Patients will be asked to complete the family history questionnaire.

18.2 Molecular analysis of primary tumours, ipsilateral and contralateral recurrence and new primary tumours

Local tumour relapse remains a clinical problem in a minority of women. The likelihood of local relapse may be influenced by genetically regulated factors, including the extent of intraductal spread and factors influencing radiation resistance. Genome-wide approaches offer scope to identify DNA sequence differences (mutations and polymorphisms) that discriminate between patients who suffer a local relapse and those who remain disease-free. Relapses that occur close to the site of the primary tumour are assumed to be true local recurrences (sharing the same gene mutations), whereas those occurring elsewhere in the breast and often at a later point in time are assumed to be new primaries (with differences in mutations compared to the primary tumour). Genome-wide approaches offer scope for investigating the genetic relationships between ipsilateral and contralateral tumour relapse and primary tumour in a systematic way that may guide future local therapies.

It is also be possible to investigate loss of heterozygosity (LOH) in breast cancer by comparing DNA extracted from the tumour samples with genomic DNA extracted from the blood samples (see 18.1).

It is proposed to establish tissue arrays and also extract DNA and RNA from paraffin blocks of primary tumours and both ipsilateral and contralateral relapses/new primaries in as many patients as possible for future comparitive studies of the cancer genome of original tumour and recurrence. Paraffin blocks containing the primary tumour and any subsequent recurrence/new primary from either breast will be sent to KCL/Guy’s and St. Thomas’ Hospital Breast Tissue Bank, London, where they will be stored for future creation of tissue microarrays and DNA and RNA extraction. In certain centres the same samples described above will be fresh frozen and sent to KCL/Guy’s and St. Thomas’ Hospital Breast Tissue Bank for the same analyses. The KCL/Guy’s and St. Thomas’ Breast Tissue Bank is an Human Tissue Authority licensed facility. After tissue cores and sections have been taken, the tumour paraffin blocks will be returned to the relevant pathology laboratory.

For LOH studies a sample of the donated blood stored at the Cancer Research UK/MRC Tissue bank at Ninewell’s Hospital, Dundee will be requested.

18.3 Quality of Life and Health Economics Studies

There is evidence that radiotherapy causes long-term effects on quality of life in terms of altered breast appearance, breast pain and other physical symptoms, notably fatigue. In this trial comparing different radiotherapy approaches, women’s subjective views of their body image and other QL parameters together with their experience of relapse if it occurs, need to be ascertained in order to compare the trade off between local tumour control and adverse effects of treatment. The key effects of treatment and relapse on QL are hypothesised to be on breast and arm symptoms, body image and psychological distress as well as general symptoms such as fatigue, and physical functioning.

A cost effectiveness analysis will be undertaken to compare the alternative interventions in IMPORT LOW. Costs will be estimated from the perspective of the NHS and Health effects and quantified in terms of quality adjusted life-years.

19. ETHICS COMMITTEE APPROVAL

This trial has been approved by Oxfordshire Research Ethics Committee B on 12/10/2006. Participants will also need the approval of their Local Research Ethics Committee.

20. PATIENT INFORMATION

The importance of providing a high level of information to patients is recognised. Patients will be informed of the services offered by Cancerbackup in the trial information leaflets. The publications we aim to provide include Clinical Trials (booklet prepared by The Royal Marsden NHS Trust) and Breast Cancer (booklet prepared by Cancerbackup). Local leaflets on radiotherapy should also be provided, but these must be approved by the appropriate committees before distribution. Each patient invited into the trial will receive an information sheet, which will include mention of the Quality of Life Study and Economic Evaluation and collection of biological specimens. They will be encouraged to participate in these studies but if they decline, this will not exclude them from the main trial. In addition, the long-term side effects of radiotherapy to the breast area and the likelihood of these developing post treatment will be explained.

APPENDIX 1

PARTIAL BREAST IRRADIATION

1. Rationale for partial breast irradiation

Breast cancer multifocality has been studied in a group of mastectomy patients who would have been eligible for breast conservation surgery (23). This pathological study illustrated that the density of tumour foci decreased with distance from the reference tumour. For invasive breast tumours ≤ 2 cm, 28% had non-invasive foci at a distance of greater than 2 cm from the reference tumour, and 14% had invasive tumour foci at the same distance. If the 264 invasive cancers in this series had been removed with a margin of 4 cm, only 7% of the patients would have had invasive cancer left in the remaining breast tissue. Randomised trials of breast conservation with or without radiotherapy have also shown that tumour recurrences usually occur close to the site of the original tumour. The NSABP B-06 trial reported that 86% of local recurrences were within or close to the reference quadrant (49). The Milan trial had similar findings, with 79% of recurrences occurring at or close to the original tumour site (50). This pattern of local recurrence occurring most frequently in the vicinity of the original tumour has led to a number of studies investigating the effect of partial breast irradiation. These studies can be divided into brachytherapy, intra-operative radiotherapy, and external beam techniques.

2. Brachytherapy techniques

Initially, low-dose rate (LDR) brachytherapy implants to the tumour bed were used as sole radiation treatments (51, 52). However, in recent years, high dose rate (HDR) techniques have been reported in the literature (53-58). The cosmetic result has generally been good, but some studies have reported higher than expected recurrence rates (51, 59). One explanation for these poor results may be due to inadequate treatment margins around the tumour bed. Secondly, the trials with higher recurrence rates included more women with extensive intraductal component (EIC), infiltrating lobular features, young age, node positivity and involved/close excision margins.

Another method of delivering partial breast brachytherapy is the MammoSite balloon breast brachytherapy catheter, which was approved by the US FDA in May 2002. This can be placed in the tumour cavity either at the time of surgery, or afterwards under local anaesthetic. The balloon is inflated with saline and contrast agent and connected to a HDR brachytherapy source. The main factors limiting use of the device were inadequate skin to balloon distance and poor balloon–cavity conformance (60). This technique will form part of a randomised controlled trial comparing partial and whole breast irradiation (see External beam radiotherapy techniques).

3. Intra-operative techniques

The technique of intra-operative radiotherapy using a portable electron beam-driven device has the advantage of delivering partial breast irradiation at the time of surgery and avoiding outpatient visits for external beam or HDR brachytherapy. It has the disadvantage, however, that the definitive histological resection margins are unknown at the time of irradiation (61). Veronesi *et al* have considerable experience of using ELIOT (electron intra-operative therapy) which consists of a mobile linear accelerator with a robotic arm (62). There is now an on-going trial in Milan which randomises to either whole breast radiotherapy or ELIOT following quadrantectomy. Another intra-operative radiotherapy device is the Intra-beam, a portable device which delivers 50 KV photons (63). A multicentre randomised trial is currently

underway which randomises breast conservation patients to whole breast radiotherapy or Target (targeted intra-operative therapy)(61).

4. External beam radiotherapy techniques

An early study of external beam radiotherapy (EBRT) partial breast irradiation had disappointing results. The Christie trial randomised 708 breast conservation patients to limited field (LF) radiotherapy to the tumour bed or to wide field radiotherapy to the whole breast and regional nodes (WF) (64). The actuarial breast recurrence rate (first event) was 15% (LF) versus 11% (WF) for infiltrating ductal carcinoma, whereas, for infiltrating lobular carcinoma, the recurrence rate was 34% (LF) versus 8% (WF). A high actual recurrence rate of 21% (LF) and 14% (WF) was also found for extensive intraduct component (EIC). Even when the lobular carcinoma and EIC were excluded from the analysis, there was still a worse recurrence rate in the LF group. This may have been due to a geographical tumour miss in the LF treatment arm, as radiotherapy planning was based on clinical assessment rather than using specific imaging techniques. In addition, other patient characteristics such as node positivity (nodal status was unknown in all) and positive margins (present in 56%) probably contributed to the higher recurrence rate in the LF group.

Using the same stringent patient selection criteria as for their brachytherapy studies, the William Beaumont Hospital, USA have used a CT planned 3-dimensional conformal (3D-CRT) technique for partial breast irradiation (65). Potential advantages of this approach over brachytherapy are: elimination of a second surgical procedure and improved dose homogeneity within the target, which may improve cosmesis and decrease the risk of fat necrosis. Possible disadvantages of 3D-CRT are that additional margins must be added to the target to account for patient movement and organ motion. This may result in a larger breast volume irradiated than with brachytherapy, which could impact on the cosmetic result. The prescribed dose was 34 Gy in 5 patients and 38.5 Gy in 4 patients, delivered in 10 fractions twice daily over 6 consecutive days. No skin changes occurred during treatment and only mild hyperpigmentation and/or erythema was observed at the 4-8 week follow-up visit but clearly, longer follow-up is required.

The Radiotherapy Oncology Group of the American College of Radiology is currently testing the feasibility and efficacy of 3D conformal radiotherapy confined to the lumpectomy cavity in women with low risk early breast cancer, within a non-randomised phase II trial. The study consists of 10 fractions of 3.85 Gy to the tumour bed, and there are plans to proceed to a NSABP/RTOG randomised phase III trial using 25 fractions of whole breast radiotherapy as the control arm, with the same phase II dose and fractionation schedule. Participating centres will be able to choose between interstitial brachytherapy, MammoSite brachytherapy and 3D conformal external beam radiotherapy for partial breast irradiation.

There appears to be a sound basis for partial breast irradiation, but mature data from randomised trials is needed to give information regarding local recurrence and late normal tissue morbidity. There are 2 key questions pertaining to these techniques. Firstly, which patients should be selected for this treatment and secondly, what margin should be used around the tumour bed? It is likely that many institutions will opt for an external beam technique for delivering partial breast irradiation because of availability and familiarity of the equipment. Accurate localisation of the tumour cavity and assessment of radiotherapy margins for external beam irradiation techniques, are therefore essential for this approach.

APPENDIX 2

EQUIVALENT TOTAL DOSES USED IN THE IMPORT LOW TRIAL

Equivalent doses using 15 fractions:

Trial arm	Target volume	Fx	Prescribed dose per fraction	Late effects [a]EQD_2	Tumour [b]EQD_2 no repop	Tumour [c]EQD_2 with repop
Control arm 15 fractions	Whole breast (no boost)	15	2.67 Gy	45.2 Gy	44.5 Gy	52.9 Gy
Test arm 1 15 fractions	Low dose volume	15	2.4 Gy	38.7 Gy	38.4 Gy	45.6 Gy
	Standard dose volume	15	2.67 Gy	45.2 Gy	44.5 Gy	52.9 Gy
Test arm 2 15 fractions	Standard dose volume	15	2.67 Gy	45.2 Gy	44.5 Gy	52.9 Gy

EQD_2: Equivalent total dose delivered in 2.0 Gy fractions
[a] Assuming α/β = 3.2 Gy for late effects.
[b] Assuming α/β = 4.0 Gy for local tumour control and no time factor (D_{prolif} =0 Gy/day).
[c] Assuming α/β = 4.0 Gy for local tumour control and time factor (D_{prolif}=0.6 Gy/day).
All schedules normalized to an overall treatment time of 5 weeks.

NOTE: the assumed fractionation sensitivities (α/β values) for late normal tissue effects and for tumour control are based on data from the Royal Marsden Hospital/Gloucestershire Oncology Centre breast fractionation trial that are now incorporated into the START Trial dataset. They are incorporated here with the permission of the START Trial Steering Committee and with the approval of the START Trial Data Monitoring and Ethics Committee.

APPENDIX 3

RADIOTHERAPY TARGET VOLUMES

1. Clinical Target Volumes (CTV)

Partial Breast CTV

The partial breast CTV is not a precise anatomical entity. It relies heavily on whole organ sectioning of 130 mastectomy specimens with unifocal tumours up to 2 cm diameter, in which a 5% frequency of intraduct and a 5% frequency of invasive foci of disease > 4 cm were found beyond the pathologically estimated edge of primary tumour (rates were 7% and 4% respectively in 264 patients with tumours up to 4 cm diameter) (23). The implication of this work is that a CTV of 40 mm around the edge of the surgical cavity would achieve greater clearance of residual malignant foci, depending on the radial margins of surgical excision. These pathological correlates are supported by the NSABP B-06 and Milan randomised trials of conservation breast surgery with or without post-operative radiotherapy; 86% and 79% of tumour recurrences occurred in or close to the reference quadrant (20, 66).

In the IMPORT LOW Trial, the tumour bed should be identified using a recommended imaging modality. Using surgical clips/gold seeds, this would consist of the clips/seeds and any change in surrounding tissue architecture, as defined by the William Beaumont group (67). Using ultrasound or MRI, this would consist of the tumour cavity. A minimum CTV margin of 15 mm should be added around the surgical cavity: this is used by the William Beaumont group, who have a large partial breast radiotherapy cohort and low recurrence rates (67).

CTV should be modified according to the individual breast anatomy. This will limit the dose to the surrounding organs at risks (OARs). For example, the posterior margin should not extend beyond the deep fascia (unless clearly breached by the tumour) and therefore, exclude the underlying muscle and ribs. Accurate visualisation of the position of the deep fascia is dependent on the quality of the imaging modality, e.g. multi-slice CT or MRI compared with limited slice simulator-CT images. If the anatomy of this region cannot be easily visualised, the posterior margin should not extend beyond 5 mm anterior to the lung/chest wall interface (this has been adopted as standard practice in other institutions) (65). The CTV should not extend radially beyond the edges of the visible/palpable breast. The anterior extent of the CTV should be limited to 5 mm below the skin surface to reduce late normal tissue skin changes. The CTV margin may be increased depending on the surgical procedure and localisation technique e.g. if surgical margins are less than 5 mm or ultrasound localisation is used (this imaging modality tends to produce smaller volumes when directly compared to clip localisation – see Appendix 4, page 39). The actual CTV around the tumour bed should approximate to the volume of a breast quadrant and therefore reflect the conceptual CTV margin around the tumour.

Whole Breast CTV

This should include the soft tissues of the whole breast down to the deep fascia. This is based on the recommendations from the START (Standardisation of breast radiotherapy) Trial (39).

2. Planning Target Volumes (PTV)

A margin should be added to each CTV, which takes into account set-up error and patient movement (including breast swelling and breathing). Several studies have used electronic

portal imaging devices to quantify the extent of positional errors and patient movement for breast radiotherapy (68-71). Three studies calculated a weighted standard deviation of the central breast distance (reflecting movement in the anterior-posterior direction) of 4.5 mm, 4.6 mm and 2.2 mm respectively for the systematic component of set-up error (68) (69-71). Four studies calculated a weighted average standard deviation of 3.9 mm, 6.1 mm, 2.0 mm and 4.7 mm respectively for systematic variation in set-up error for the cranio-caudal distance (reflection movement in the superior-inferior direction) (71) (68-70, 72). Due to the variation in reported measurements, Hector *et al* adopted a value of +/- 3 mm for displacement in both directions, to model the effect of set-up error and breast volume change on conventional and intensity modulated radiotherapy (IMRT) breast radiotherapy (73). They found that whilst IMRT was slightly more susceptible to movement than conventional radiotherapy, the final dose distribution was always superior, hence justifying the use of IMRT in the presence of set-up errors. Another study found that a vac-fix immobilisation device was superior to a breast board as it improved transfer of the planned set-up from the simulator to the treatment unit (74). It was felt that implementation of the vac-fix device was not justified for standard tangential breast radiotherapy, but may be important for more complex techniques such as IMRT.

It is difficult to determine from the portal imaging studies exactly which part of the displacement was due to set-up error and which was due to patient movement. Hector *et al* showed that the average increase in breast volume during treatment was 5%, and this peaked between fractions 5 and 8 and then decreased back below the initial volume (71). It has been stated that the effects of breathing motion are in general about half the size of the effects of set-up error (73). Breathing motion may be particularly important in dynamic-MLC IMRT techniques, and a study has shown that dosimetric errors are dependent on the speed of the travelling leaves relative to the speed of the target motion (75). However, later studies have shown that these average out over a full treatment course and should not be a source of problems. Certain centres may wish to implement methods to limit breathing motion such as gated radiotherapy and breath-holding techniques (76).

One institution developing 3D-CRT for partial breast irradiation, measured the impact of patient set-up error and breathing motion to establish CTV to PTV margins (65). This was then tested clinically for adequate coverage of treatment. The CTV-PTV margin for ‘breathing only’ was calculated by measuring the displacement of surgical clips during 3 types of CT scan: free breathing, and breath holding at the end of normal inhalation and at the end of normal expiration using an active breathing control device. A margin of 5 mm was subsequently selected to completely account for breast motion during quiet breathing. The combined uncertainty of random patient set-up error and respiratory motion, and the distribution of systematic error across all fields and all patients, were measured. This was achieved by measuring the movement of the chest wall/ribs with portal imaging, as a surrogate for the tumour bed. A margin for set-up uncertainties of 5 mm was proposed from this data, producing a total CTV-PTV margin of 10 mm, which was tested in 9 patients. 98 - 100% of the CTV was covered by the 95% isodose surface at the extremes of normal inhalation and exhalation using the ‘breathing only’ margin of 5 mm. The total CTV-PTV margin of 10 mm also seemed to provide coverage for most patients. The authors state that there is still uncertainty regarding the stability of the tumour cavity relative to the chest wall and that this may vary more in patients with larger breasts. Therefore, slightly larger CTV-PTV margins may be needed in this group of patients.

Given the reports from the literature, a typical PTV margin may be about 10 mm. The margins for PTVs should ideally be determined by each centre to reflect accuracy of set-up and estimation of patient movement for that institution (there may be existing information available from the START QA team). 3D growing algorithms should be used where possible and centres may wish to develop asymmetric volume growth if it is felt that one direction is more prone to inaccuracies than others. The PTV should stop 5 mm beneath the skin surface in order to preserve skin sparing.

3. Radiotherapy field boundaries

The field boundaries should cover the PTVs with a sufficient margin for beam penumbra. This would typically be of the region of 5 mm, though in the anterior direction the field border should be 1.5 – 2.0 cm beyond the skin surface to allow for breast swelling.

The field arrangement for the partial breast radiotherapy will consist of 'mini-tangents'. Therefore, the anterior and posterior dimensions of partial breast fields are likely to be the same as the whole breast fields. The field length in the superior-inferior direction, however, will be shortened. For example, field lengths will be determined by localising the tumour bed, adding 15 mm CTV margin (or less if the superior or inferior limits of breast tissue are reached), 10 mm PTV margin, and 5 mm for beam penumbra, to above and below the tumour bed. Overall field length will vary between patients, but will often be 80 – 100 mm in length.

4. Organs At Risk (OAR)

Organs at risk (OAR) include the heart, lung and chest wall. Definition of the OAR will be limited by the quality of the imaging modality. A minimum requirement would be to report the volume of heart and lung actually within the radiotherapy field. Ideally dose-volume histograms should be created for the heart and lung and this would require knowledge of the entire volume of these organs. For those centres using a limited number of CT slices for planning, algorithms could be developed to interpolate the lung/heart outline between slices. In addition, the simulator films of standard tangential fields could be used to give an estimation of internal organ position.

APPENDIX 4

LOCALISATION OF THE POST-OPERATIVE BREAST TUMOUR CAVITY

Planning of the radiotherapy treatment to the tumour bed requires an assessment of the location of the post-operative tumour cavity. In general, this is done using a combination of information: pre-operative radiological imaging, surgical annotation, clinical palpation of the surgical defect and position of the breast scar, and patients' recollection of the site of the mass. In the past, the position of the scar has been heavily relied on to assist with locating the tumour bed. However, breast surgical technique has subsequently changed, with the scar frequently being placed some distance from the site of the tumour in order to achieve a better cosmetic result. This has prompted some institutes to compare traditional 'clinical' methods of boost planning with various imaging techniques.

1. Surgical clips for localisation of the tumour cavity

Several studies have reported the superiority of using surgical clips to locate the tumour bed compared with clinical methods (77-83). All studies showed that the tumour cavity would have been under-dosed using traditional planning techniques. The clinical method could also result in a substantial volume of normal tissue being irradiated unnecessarily (83). In addition, it was reported that medially and laterally located tumour cavity could also be missed by the tangential fields (78, 82).

Fein *et al* hypothesised that radiotherapy planning using surgical clips should define the tumour bed more accurately and therefore less local recurrences should be seen (70). They devised a study to compare the incidence of breast recurrence in women with and without surgical clips, controlling for recognised prognostic factors. Overall, there was a significant difference in the 10-year breast recurrence favouring women without clips despite more adverse prognostic factors. However, a single surgeon had a 10-year breast recurrence rate of 21% compared with 6% and unknown margins in 48% compared with 10% overall. Excluding this surgeon from the analysis resulted in no significant difference between the 2 groups. It was concluded that unknown margins, due to failure to ink the specimen, could not be compensated by better visualisation of the tumour cavity using surgical clips.

Detailed descriptions of the planning techniques using surgical clips have been reported using both computed tomography (CT) scanning and simulator films (84, 85). A consistent policy of clip placement at the time of surgery is necessary. An example of this is to place a pair of clips at the medial, lateral, superior and inferior extent of the tumour bed, and a fifth pair at the deepest extent of the tumour bed in the direction of the surgical excision (84). A sixth anterior pair of clips may also be placed under the skin and subcutaneous tissue. There have been reports of surgical clips becoming dislodged and tracking away from the tumour site, but this appears to be a rare occurrence. The potential problem is overcome by the placement of pairs of clips. More commonly, the orientation of the clips can change during surgical closure (Personal communication, Mr A Thompson, consultant breast surgeon).

2. Ultrasound for localisation of the tumour cavity

Breast ultrasonography has also been exploited as a method of localising the tumour bed for radiotherapy planning. A study compared clinical methods with ultrasound localisation and found that the full extent of the tumour cavity was underestimated in 87% of women, and the chest wall depth was incorrectly estimated in 90% using traditional methods (86). Another study reached similar conclusions: conventional electron boost planning resulted in 55% of patients having areas of under-treatment and 20% of patients receiving significant over-treatment (87).

The location and appearance of the tumour cavity has been found to be highly reproducible on repeated scans, with a mean depth difference between scans of 2 mm (88). There is some discrepancy as to whether the ability to localise the tumour cavity is more difficult with increasing time from surgery. This is important to consider with many women receiving up to six months of adjuvant chemotherapy prior to irradiation and the current UK problem of long waiting times for radiotherapy treatment. One study reported that it was difficult to visualise the cavity after 8 weeks from surgery (86). This view was reflected by another study, which found that the optimal time for radiotherapy planning was within 60 days post-operation (89). Other reports contradict this view, stating that the tumour cavity can be seen many months following surgery (88) (personal communication, Dr R Sinnatamby, consultant radiologist). Interestingly, one study commented that the tumour cavity decreased in size during radiotherapy treatment (89). Therefore, it may be important whether the boost is planned before or towards the end of radiotherapy.

All reports in the literature have used 2-dimensional (2D) ultrasound scanning techniques. This is perfectly adequate for placement of a direct anterior electron boost field, as the dimensions of the cavity with a suitable margin can be marked on the patients' skin and the electron energy can be selected from measurement of the cavity depth. However, other radiotherapy techniques such as a brachytherapy interstitial implant or a concomitant boost to the tumour bed using intensity modulated radiotherapy (IMRT), require more detailed 3D information. This can be achieved by using a combination of ultrasound examination and placement of radio-opaque skin markers and measurement of cavity depth, followed by CT scanning in the same position (90). Another novel method is to use a 3D ultrasound scanning technique (currently under investigation at Addenbrooke's Hospital, Cambridge). This requires spatial registration of the ultrasound scan with fixed points around the breast using a camera system. A 3D volume of the tumour cavity can be produced, which is then imported into the radiotherapy planning system.

3. Magnetic Resonance (MR) imaging for localisation of the tumour cavity

Magnetic resonance (MR) provides excellent definition of the breast and surrounding tissues. Its use in breast radiotherapy planning, however, has been very limited. This has largely been due to a combination of limited MR resources and the difficulty of scanning the patient in the treatment position. The Bristol Haematology and Oncology Centre have experience in the use of a low-field open MR scanner for breast radiotherapy planning, which allows imaging in the treatment position (91). This group has demonstrated with MR imaging, that conventional breast radiotherapy planning of the boost and sometimes the tangential fields, can result in under-treatment of the target. In addition, greater sparing of surrounding organs at risk can be achieved with MR-assisted planning. Potential problems with MR radiotherapy planning include image distortion and co-registration with radiotherapy planning systems.

4. Tumour bed localisation methods for IMPORT LOW

4.1 Surgical clips

Each centre must adhere to a protocol for the placement of titanium clips or gold grains at the time of surgery. This will enable consistent interpretation of clip position for radiotherapy planning. CT scanning or a robust simulator film method should be used to visualise the clips (84, 85).

4.2 Ultrasound

A combination of ultrasound and CT scanning can be used to visualise the tumour cavity in 3-D (90). A 3D ultrasound method is currently under investigation as an alternative to using surgical clips, and may be used if the IMPORT Trials Office is informed.

4.3 MR imaging

MR imaging can be used to obtain anatomical information of the tumour cavity and surrounding tissues. Ideally, this should be available electronically in the radiotherapy planning system.

4.4 General recommendations

The patient must be scanned in the treatment position for all imaging modalities. Each centre must develop their own localisation protocol according to which of the 3 methods is available. Centres with established methods could assist with this development process. A central Quality Assurance team must assess and approve all localisation techniques.

APPENDIX 5

SUGGESTED SIMPLE METHOD FOR BREAST COMPENSATION USING 1-2 EXTRA MLC SEGMENTS

1. A simple manual design for one or two extra segments added to a good, tangential pair plan requires:
   - An outlining system where multislices may be efficiently gathered.
   - A means of transferring these to a planning system with a beam eye view facility and a means of viewing a sagittal slice through the breast.

   (Although, a planning system with a facility to create multi-segments inside a beam is useful, the segments, as they are few, may be separate beams).

2. A sufficient number of transverse sections of the patient are required, either from CT or another valid outlining system e.g. Osiris (QADOS). It is recommended that there are no less than seven slices and preferably more, spaced appropriately throughout the volume.

3. The slices are transferred to the planning system, and the geometry set up as for a standard wedged tangential pair. A PTV may be defined, using the 50% isodose for the patient contour as an indication of irradiated volume should be sufficient for plan analysis.

4. A good wedge only plan is produced.

5. A sagittal view through the breast is created with the appropriate isodose lines displayed. For some planning systems this may be overlaid in the BEV window; for others it may be necessary to transfer it to a transparency to use as a template for designing the fields. Where CT data exists then careful windowing of DRRs may allow segment design in the BEV.

6. Create an additional segment for each beam either within the field or as a separate beam. This will be treatment planning system dependent. The new segment/beam may be orientated to optimise the fitting of the MLC leaves. The sagittal view/template/windowed DRR is used to set the MLC leaves to cover e.g. the 107% isodose lines. If higher isodose lines e.g. 110% are seen, a second segment/beam may be used in the same way to cover these.

7. The segments are weighted at 5 - 8% of the isocentric weight of the wedged fields.

8. The design is repeated for the other tangential field.

9. The plan is calculated and assessed. A small amount of iteration to the field shape and/or beam weight may be required to reduce the high dose volume to the level required.

APPENDIX 6

TREATMENT VERIFICATION

Verification of standard breast radiotherapy relies on:

- Daily checking the light field border against tattoos or reference marks at relatively stable anatomical positions;
- Checking that the whole breast is covered adequately by the light field;
- Checking central axis focus skin distance;
- Portal verification using chest wall coverage and lung depth as guidance for acceptable set-up.

Under the current IMPORT LOW proposals one aim is to seek to exclude lung and ribcage by judicious placing of field borders. This will have the effect of moving the light field border(s) onto less stable breast tissue, and removing reference anatomy from the portal images. Partial breast irradiation introduces other potential problems for treatment verification (e.g. verification of tumour bed position). These proposals put forward a simple rationale that will facilitate consistent verification.

The following factors have been taken into account in deciding on these proposed verification methods: the light field border verification should continue to be done at stable points; the same verification method should be used irrespective of trial arm or tumour location; the portal verification should have reference anatomy included. Partial breast irradiation means that the tumour bed/index quadrant does not fall outside the high dose zone at any time during treatment.

The proposals for verification are as follows:

- A simulated verification field should be used for patient set-up (i.e. use of a non-treatment image). The verification field will cover the whole breast and extend to stable midline and lateral points. This will allow daily light field verification (as occurs at present) irrespective of trial arm or tumour location. Practically, the isocentre would be established and the pre-determined verification field used to check alignment with medial and lateral tattoos (see Figure 1). The field border positions for the treatment fields would be related to the stable tattoos.
- The same verification field would be used for portal verification. This would include reference anatomy, and would provide the same verification irrespective of trial arm or tumour location. The treated area could be highlighted by double exposure if desired.

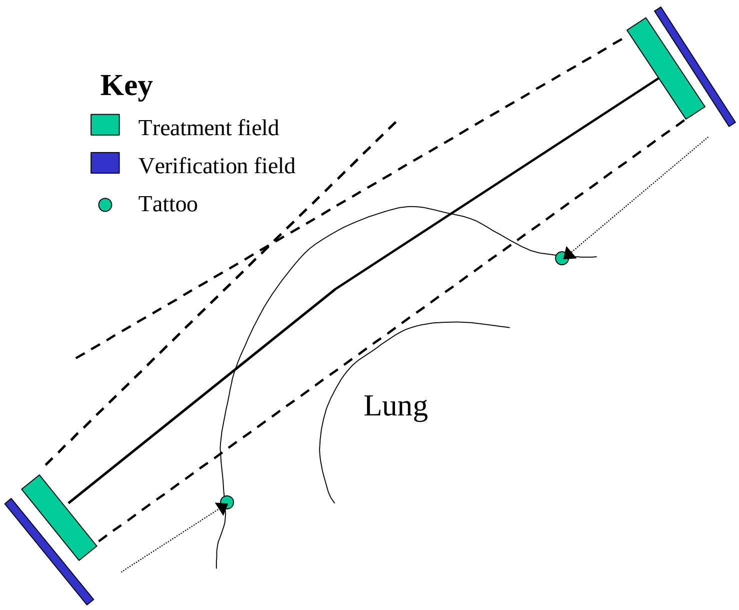

Figure 1. A schematic view of the verification field showing the alignment with

Portal verification should be at least weekly to give confidence that the tumour bed/quadrant area has not fallen outside the treated area. A typical protocol to correct for systematic error would be to obtain three verification images in the first week in order to correct the error and thereafter weekly. This may be a change from current practice, but is deemed necessary because margins are tighter. The impact on workload this will have will be small if the change is applied to trial patients only.

APPENDIX 7

QUALITY ASSURANCE PROGRAMME

1. Background

The complex nature of modern radiotherapy carries inherent problems both in ensuring reproducibility and accuracy within a radiotherapy unit and, more particularly, when carried out on a multi-centre basis. Specific issues in the treatment of the breast and lymph node pathways arise from the geometry of the treatment volume which varies in contour in all three planes with important radiation sensitive structures underlying the breast and chest wall including the lung and myocardium. Careful localisation, computerised planning, accurate verification of beam position and meticulous attention to alignment and matching during treatment are essential.

A quality assurance programme is “a mandatory prerequisite when aiming at high dose, high precision radiotherapy” and is an integral component of any radiotherapy trial as defined by the EORTC guidelines for trial protocols in radiotherapy (92, 93).

In this multi-centre randomised trial the quality assurance programme will enable confirmation that technical guidelines within the protocol have been understood and implemented correctly by participants and that the dose prescription is delivered according to protocol together with appropriate documentation of technique and patient related data. This will ensure that clinical observations in terms of tumour control and normal tissue damage reflect differences in the randomised schedules rather than departures from trial protocol. Techniques used will be documented, this data will be available should differences in observed end points emerge.

In this way the definition of quality assurance as “all those planned and systematic actions necessary to provide adequate confidence that a produce will satisfy given requirements of quality” (94) can be satisfied and the scientific worth of the parent trial be validated.

The QA programme will build on that developed for the START trial. This has provided an element of consensus in radiotherapy technique amongst radiotherapy centres. IMPORT LOW will necessitate the implementation of new technology in many centres where the use of compensation or intensity modulated radiotherapy has not been used previously. Around 25 centres in the UK currently use the technology that is proposed for IMPORT LOW in selected groups of patients.

2. Plan of investigation

The quality assurance programme will follow the guidelines set out by the EORTC (93) and will be co-ordinated by an experienced QA team based at Mount Vernon Hospital (95, 96). It is based on anticipated accrual to around 35 centres over a three and a half to four year period. The programme will proceed as follows:

2.1 An initial questionnaire establishing precise details of technique to be used within the centre, together with specimen patient outlines to be used for ideal plans to be produced by each centre.

2.2 A visit by the quality assurance team prior to a centre entering the study to validate independently the technique in use against the information given in the questionnaire. In particular, the following parameters will be assessed:

i) Confirmation of IMRT/compensator implementation (small field monitor etc).

ii) Planning of radiation distributions across the treatment volume for homogeneity and prescription points.

iii) Routine QC performed by the centre will be assessed and compared with current IPEM guidelines (97).

vi) Measurements across the treatment volume within a purpose-made breast phantom, with particular reference to dose homogeneity.

2.3 All plans together with corresponding CT data sets will be collected electronically. Data should be anonymised with the patient's trial number and initials prior to sending to the QA team. Verification images will also be collected for the first 3 patients. In exceptional circumstances if electronic transfer of data is not possible, hardcopy plans for the first 3 patients in each treatment arm, and subsequently 1 in 10 plans together with verification images, will be collected by the QA team, to ensure continued protocol adherence.

2.4 It is anticipated that a subset of approximately 1 in 10 patients within the trial will have thermo-luminescence dosimetry (TLD) sent from the QA team. These patients will be identified at randomisation.

3. Quality control by department for Compensated Plans

Where an established breast compensation programme is not set up, some additional checking may be required depending on the planning approach. If MLC segments are to be used then small segment, low monitor units checks for symmetry, flatness and dosimetry are required (98). Compensated plans should have additional quality control performed. For at least the first 5 patients this may include verification of each field fluence map and absolute dose measurements. Subsequent to this the quality control may be reduced in line with departmental practice but the QA team should be notified.

4. Analysis of QA programme

The data from the quality assurance programme will be analysed separately from the main trial. Major discrepancies from trial protocol will be notified to participating centres. These will include:

i) Discrepancies in documentation, dose prescription and dose recording.

ii) Dose inhomogeneity of more than 12% across breast treatment volume (-5% to +7%).

iii) Inclusion of > 2 cm of lung in treatment volume.

iv) Systematic errors of technique in any stage of treatment from planning through to implementation.

The detailed analysis of the quality assurance data will produce quality information covering the following areas:

i) Variations in breast radiotherapy practice in participating centres.

ii) A comparison of methods used for compensation (physical compensators, multiple static fields, dynamic fields).

iii) An assessment of the emerging technologies and their quality control.

iv) Quantification of dose uniformity during the treatment period.

v) Correlation of physical parameters of radiation with trial end points:

- The association between dose variation across the treatment volume and tumour control.
- Dose variation, machine energy and skin surface doses in relation to moderate/severe fibrosis and breast shrinkage.
- Variations in dose homogeneity with rib pain, fracture and necrosis.

APPENDIX 8

SERIOUS ADVERSE EVENT REPORTING

Definitions:

Adverse Event (AE): Any untoward medical occurrence in a patient or clinical trial subject administered a research procedure; events do not necessarily have a causal relationship with the procedure.

Related Adverse Event: An adverse event assessed by the Principal Investigator or Chief Investigator as reasonably likely to be related to the administration of a research procedure.

Serious Adverse Event (SAE): an untoward occurrence that:

1. results in death
2. is life-threatening
3. requires hospitalisation or prolongation of existing hospitalisation
4. results in persistent or significant disability or incapacity
5. consists of a congenital anomaly or birth defect
6. is otherwise considered medically significant by the principal investigator

Reporting procedure:

1. All SAEs must be reported within 24hrs of the event being identified using the specific SAE forms. These must be completed, signed and dated by the Principal Investigator or delegate named on the delegation form.

2. The SAE form must be faxed to the IMPORT trials office at the ICR-CTSU on:

   020 8722 4368

3. The hard copy must be sent by post to the IMPORT trials office using the address on the SAE form.

4. The IMPORT Trials office will send a fax to acknowledge receipt of the SAE.

5. Follow-up information should be completed on the relevant part of the original SAE form within 15 days of the initial report and faxed to the trials office.

6. The Chief Investigator or delegate will review all SAEs to assess the “expectedness” of the event.

7. The Site SAE log should be completed and the SAE form filed in the Site Investigator File.

APPENDIX 9

QUALITY OF LIFE STUDIES

Quality of life (QL) study protocol

Rationale for QL study

There is evidence that radiotherapy causes long-term effects on quality of life in terms of altered breast appearance, breast pain and other physical symptoms, notably fatigue. In this trial comparing different radiotherapy approaches, women's subjective views of their body image and other QL parameters together with their experience of relapse if it occurs, need to be ascertained in order to compare the trade off between local tumour control and adverse effects of treatment. The key effects of treatment and relapse on QL are hypothesised to be on breast and arm symptoms, body image and psychological distress as well as general symptoms such as fatigue, and physical functioning.

The approach to QL in this trial will mirror the successful QL protocol in the START Trial and other national trials of breast cancer treatment. Results will therefore be comparable across a wide range of patients and treatment experiences.

Rationale for QL measurement

The main priority guiding the evaluation strategy is to select standardised QL scales and subscales that will answer the research questions of importance in this study and allow comparison with other relevant trials. The scales selected include a general cancer QL scale, plus specific measures for breast cancer, body image and psychological distress.

Measures

The EORTC QLQ-C30 (99) is a purpose-developed self report scale for use with cancer patients, which has been well tested psychometrically and is being widely used in clinical trials. The EORTC BR23 breast cancer module is a 23-item scale designed for use with the core instrument in breast cancer treatment (100). A 10-item Body Image Scale (BIS) designed for use with cancer patients will also be included (101), which has been used in other national breast cancer trials, and for which extensive reference data are available. Psychological distress will be measured by the Hospital Anxiety and Depression Scale (HADS) (102) which has been widely used in clinical trials to date and provides clinically interpretable outcomes.

The QL endpoints are designed to complement the external assessments of breast appearance and other late normal tissue effects, and to capture the medium- and long-term sequelae of breast radiation therapy on health-related quality of life. The QL study is both descriptive and comparative: sample size considerations are addressed where appropriate.

Feedback from compliance data and interim analysis in the START trial will guide the optimal timing and mode of administration of QL questionnaires, especially at the time of relapse. A suitable policy will then be adopted for IMPORT LOW QL data. This protocol will be available to a limited number of centres wishing to participate in the QL assessments. In those centres, QL endpoints will be offered to patients randomised into the main study. This approach has been very

successful in the START Trial with excellent representation of centres geographically and high levels of compliance.

The QL evaluation is described below for 2 outcomes: normal tissue effects and tumour-related effects.

The QL outcomes will be summarised in a form that can be used by clinicians to inform patients and other stakeholders e.g. providers and commissioners of health care. No weighting will be given to prioritise any particular QL domain: the aim is to provide information from all QL domains as appropriate.

Normal tissue effects

1) Breast appearance and body image
   The impact of different radiotherapy fractionation regimens will be assessed using 10 items relating to body image. Patients with local recurrence will be censored for this analysis. Associations between altered body image and psychological distress will be explored using all available data.

2) Other radiotherapy-induced adverse effects
   The proportion of patients suffering lymphoedema, shoulder stiffness, breast pain and brachial plexopathy will be assessed. Relevant symptoms from the EORTC QLQ-BR23 scored as ‘quite a bit’ or ‘very much’ will be used as an indicator of adverse effects. Limitations on functional status will be assessed using the following subscales of the EORTC QLQ-C30: physical functioning (items 1-5), role functioning (items 6,7), social functioning (items 26, 27). Again, limitations scored ‘quite a bit’ or ‘very much’ will be used as a basis for comparison between regimens.

3) Sexual functioning, psychological distress and global quality of life
   Whilst we would not assume that these parameters are influenced primarily by treatment, these domains may reflect the impact of tissue damage on altered body image - we will therefore explore these domains within regimen and describe levels of dysfunction, distress and global quality of life. Formal statistical comparisons will be considered if differences emerge which warrant testing, but these are not expected. Global QL will be measured using items 29 and 30 from the EORTC QLQ-C30. Sexual functioning will be assessed from relevant questions from the EORTC Breast Cancer Module BR23. Anxiety and depression will be assessed using the accepted threshold scores on the Hospital Anxiety and Depression Scale (HADS).

Tumour-related effects

We hypothesise that local recurrence will be associated with increased psychological distress. Women with recurrence will be compared using the HADS with those who remain disease-free. The proportion of patients with depression or anxiety will be assessed using the assessment point that occurs after confirmation of first recurrence. Subsequent assessment points will be used to explore the data and ascertain whether any increase in psychological morbidity is maintained over time. However, other ‘events’ and progressive disease or further surgery may confound accurate measurement and this will be for descriptive purposes only.

Summary of results to reflect favourable and unfavourable effects of the treatments under comparison.

In order to aid clinicians in an appraisal of the results we shall summarise the major findings, positive and negative, of the above outcomes. We will not attempt to produce a summary score representing a QL outcome for each regimen, but will report results for each domain under consideration. Results for medium and long-term effects will be presented in tabular form with accompanying explanatory paragraphs.

This will be a particularly important way of trying to provide a resume of a large study, which will help clinicians and others consider and discuss factors that influence a 'trade-off' of (psychosocial) cost and benefit, should this arise, the main one being considered to be enhanced cosmesis at a greater risk of local relapse.

Plan of study

This is a multi-centre study. The QL evaluation will not involve all participating centres, but in those centres electing to evaluate QL, it will form an integral part of the protocol for all patients entered from that centre until the required number of patients have been entered. It will be carried out together with photographic assessments. Due attention to geographic representation in the QL study will be given and the IMPORT LOW Trials Office will monitor participation by centres to ensure this.

Eligibility

All patients from selected centres who:

- are entered into the IMPORT LOW trial;
- consent to be part of the QL study and are available for follow up;
- are willing and able to complete the self-report QL questionnaires.

Sample Size

Quality of Life evaluations will be carried out in a prospective sample of 1,200 patients.

Four hundred patients per arm will provide > 80% power to detect differences of $\geq$ 5 between the means of QL subscales (assuming a standard deviation of 20), or to detect differences of ≥ 15% in the prevalence of specific normal tissue effects (e.g. lymphoedema, shoulder stiffness, breast pain) and anxiety and depression. Sample size estimate assumes a significance level of = 0.005 (to allow for multiple testing and the 1:1:1 randomisation) and allowing for 10% attrition due to illness or death (based on experience from the START trial).

The significance level chosen allows, to some degree, for the multiple testing involved in analysing individual sub-scales of the QL questionnaires. The numbers identified above also allow for some degree of attrition due to illness or death (10% non-completion). Experience from the START trial has shown compliance to be high. Particular care will be taken when approaching patients in the trial known to have relapsed, as although it is vital to collect these data, it may be requested at a sensitive time.

Timing of Assessments

The emphasis is on the long-term assessment of different treatment policies so that the number of questionnaire administrations is limited in the first year.

Baseline

Questionnaires will be handed out in the clinical centre by a designated member of staff, trained in QL administration. Patients will be asked to complete the forms after a full explanation of the study and after giving informed consent but **before** the randomisation is known, to avoid the possibility of bias. Patients will be asked to complete a demographic form with name, address and GP details.

Subsequent assessments will be mailed directly to the patient from the IMPORT LOW Trials Office at the following times after randomisation: 6 months, 1 year, 2 years, 5 years.

Due care will be taken to check the physical status of all patients prior to questionnaire mailing. This will be done through telephone contact with the hospital department and/or GP as appropriate. The follow-up questionnaires will be sent out by the IMPORT LOW Trials Office requesting completion within the week. If the forms have not been returned 2 weeks after having been sent out, there will be a telephone call to advise the patient that the forms have been sent, to check that they have been received and to prompt their completion and return. Such a mechanism also provides the opportunity to clarify any missing data with the patient on the forms which have been returned with incomplete responses. The annual follow-up assessments will be sent out shortly after the patient attends the hospital for routine annual follow-up, thereby ensuring that information on the patient's health status is up to date.

Missing data

All reasonable efforts will be made to ensure correct completion of the QL assessments. Full explanation of the QL study will be given by the responsible research nurse/member of breast care team prior to administration of the baseline questionnaires. On collection, the questionnaires will be briefly checked for completeness. The follow-up questionnaires will include instructions for completion. When individual items are missing, procedures, which have been used in similar studies, will be adopted:

- where the missing item is a single item measure this is simply recorded as a missing value;
- where the missing item forms part of a sub-scale a prorating procedure will be used depending on the total number of items on the scale and the number appropriately completed:
    - where fewer than 50% of the items of the sub-scale have been completed correctly then this constitutes a missing case for that sub-scale;
    - where 50% of the items of the sub-scale have been completed then the mean score obtained for the completed items can be inserted.

Trial management

Trials Office

The Study Co-ordinator, based in the IMPORT LOW Trials Office, will be responsible for overall co-ordination of the study. The Study Co-ordinator will liaise closely with those responsible for the QL study in each participating hospital and with the expert psycho-oncologist and clinicians involved in the project. He or she will verify the status of the patient and send out the follow-up questionnaires. Any queries regarding the patient or the patient's management will be referred to the responsible person in the hospital.

It is reasonable that individual hospitals who have the resources may wish to co-ordinate the administration of the follow-up questionnaires and forward them to the study co-ordinator within an agreed time frame.

Hospital
It is necessary for each participating hospital to identify a person responsible for the conduct of the QL protocol. This person will explain the study to the patient, ensure that the patient understands how to complete the QL questionnaire, and forward the first set of completed forms to the Study Co-ordinator. He or she will maintain close liaison with the Study Co-ordinator in the IMPORT LOW Trials Office and be responsible for organising cover in times of holiday or other planned absence.

Data Management
The Study Co-ordinator will be responsible for checking the data for consistency and completeness, for providing reminders for overdue forms to the responsible persons in the hospitals and for entering the data onto the central database for the trial.

Statistical Analysis Plan
The algorithms developed for use with the QL forms will be used to measure the parameters of interest. Groups of patients will be compared at agreed time points and overall for differences in these parameters (103). The treatment groups will be compared at the individual time points with appropriate adjustments being made for multiple comparisons. Because of the longitudinal nature of the data, an analysis which takes into account the repeated measures is also needed. A generalised linear modelling approach will be adopted (104-106). This will allow the appropriate error distribution to be used and will enable the analysis to take account of important factors such as age, stage of disease, treatment received and other sociodemographic and clinical characteristics.

Informed Consent and Ethical Issues
Details for the main trial are outlined in the Clinical Protocol. Investigators participating in the QL Study will obtain local Ethics Committee approval for the study. The investigator is responsible for obtaining each patient's signed informed consent prior to the administration of the baseline QL assessment.

Patients obtaining clinically significant scores on the HADS should be further assessed clinically. This will be explained in the Patient Information Sheet and patients will be specifically asked to consent to information about high HADS anxiety/depression scores being passed on to their doctor. The cut-off HADS score for the subscales combined used for identifying probable cases is 19, 75% of people with a score of this magnitude are found on interview to have clinically significant anxiety and/or depression which could be relieved for the majority of them by psychotherapeutic and/or pharmacological intervention. If a patient scores 19 or above on the HADS scale the IMPORT LOW QL Co-ordinator will contact her clinical oncologist.

Endpoints and measures

Measures to be used
EORTC QLQ-C30 (Academic Users Agreement obtained.)
EORTC BR-23 Breast Cancer Module (Academic Users Agreement obtained).
Body Image Scale (BIS) (Published).
The Hospital Anxiety & Depression Scale (HADS) (Published).

Quality of Life, The EORTC QLQ-C30 and Breast Cancer Module (BR23)
The QLQ-C30 is a 30-item questionnaire comprising 5 functional scales (physical, role, cognitive, emotional and social), a global QL scale, and 3 symptom scales (fatigue, pain, nausea & vomiting) and a number of single item measures.

The breast cancer specific module consists of 23 items of specific relevance to patients with breast cancer, namely side effects of breast surgery, chemotherapy, endocrine therapy and radiotherapy, body image, sexuality and future perspective.

Body Image
External (photographic) and subjective (patient self-report) assessments will be carried out prospectively.

1) ***Photographic Assessments***
   Digital photographic assessments will be undertaken post-surgery and at 2 and 5 years post-treatment, using two views, with hands on hips and hands raised as far as possible over the head. Change of breast appearance compared with the post-surgical baseline will be scored on a three-point scale by 'blinded' investigators, to define clinically relevant groups, namely no/minimal change; marked/gross change and an in-between group.

2) ***Patient Self-Assessment***
   The 10-item Body Image Scale is sensitive to change over time and discriminates between patients treated with mastectomy and conservative surgery. Four items are already incorporated in the BR23 and will be summed with the 6 additional items to form the full scale. Three protocol specific items not covered by the EORTC breast cancer module or Body Image Scale will also be included to complete the evaluation of cosmesis and radiation effects.

The Hospital Anxiety & Depression Scale - HADS
This is a 14-item scale (7 items anxiety, 7 items depression) designed to measure psychological distress in cancer patients. Threshold scores have been derived that enable the prevalence of clinically significant levels of anxiety and depression to be evaluated. A comparison of 3 screening measures suggested that the HADS was the best scale when compared against a diagnostic psychiatric interview, in patients who were disease-free or stable, and hence is the preferred measure for this trial (107).

EORTC QLQ-C30 **(version 3)**

We are interested in some things about you and your health. Please answer all of the questions yourself by circling the number that best applies to you. There are no "right" or "wrong" answers. The information that you provide will remain strictly confidential.

Please fill in your initials:
Your birthdate (Day, Month, Year):
Today's date (Day, Month, Year):

		Not at All	A Little	Quite a Bit	Very Much
1.	Do you have any trouble doing strenuous activities, like carrying a heavy shopping bag or a suitcase?	1	2	3	4
2.	Do you have any trouble taking a <u>long</u> walk?	1	2	3	4
3.	Do you have any trouble taking a <u>short</u> walk outside of the house?	1	2	3	4
4.	Do you need to stay in bed or a chair during the day?	1	2	3	4
5.	Do you need help with eating, dressing, washing yourself or using the toilet?	1	2	3	4

	**During the past week:**	Not at All	A Little	Quite a Bit	Very Much
6.	Were you limited in doing either your work or other daily activities?	1	2	3	4
7.	Were you limited in pursuing your hobbies or other leisure time activities?	1	2	3	4
8.	Were you short of breath?	1	2	3	4
9.	Have you had pain?	1	2	3	4
10.	Did you need to rest?	1	2	3	4
11.	Have you had trouble sleeping?	1	2	3	4
12.	Have you felt weak?	1	2	3	4
13.	Have you lacked appetite?	1	2	3	4
14.	Have you felt nauseated?	1	2	3	4
15.	Have you vomited?	1	2	3	4

<u>Please go on to the next page</u>

**During the past week:**	**Not at All**	**A Little**	**Quite a Bit**	**Very Much**
16. Have you been constipated?	1	2	3	4
17. Have you had diarrhea?	1	2	3	4
18. Were you tired?	1	2	3	4
19. Did pain interfere with your daily activities?	1	2	3	4
20. Have you had difficulty in concentrating on things, like reading a newspaper or watching television?	1	2	3	4
21. Did you feel tense?	1	2	3	4
22. Did you worry?	1	2	3	4
23. Did you feel irritable?	1	2	3	4
24. Did you feel depressed?	1	2	3	4
25. Have you had difficulty remembering things?	1	2	3	4
26. Has your physical condition or medical treatment interfered with your family life?	1	2	3	4
27. Has your physical condition or medical treatment interfered with your social activities?	1	2	3	4
28. Has your physical condition or medical treatment caused you financial difficulties?	1	2	3	4

For the following questions please circle the number between 1 and 7 that best applies to you

29. How would you rate your overall health during the past week?

1 2 3 4 5 6 7

Very poor Excellent

30. How would you rate your overall quality of life during the past week?

1 2 3 4 5 6 7

Very poor Excellent

© Copyright 1995 EORTC Study Group on Quality of Life. All rights reserved. Version 3.0

EORTC QLQ-BR23

Patients sometimes report that they have the following symptoms or problems. Please indicate the extent to which you have experienced these symptoms or problems during the past week.

**During the past week:**	**Not at All**	**A Little**	**Quite a Bit**	**Very Much**
31. Did you have a dry mouth?	1	2	3	4
32. Did food and drink taste differnet than usual	1	2	3	4
33. Were your eyes painful, irritated or watery?	1	2	3	4
34. Have you lost any hair?	1	2	3	4
35. Answer this question only if you had any hair loss: Were you upset by the loss of your hair?	1	2	3	4
36. Did you feel ill or unwell?	1	2	3	4
37. Did you have hot flushes?	1	2	3	4
38. Did you have headaches?	1	2	3	4
39. Have you felt physically less attractive as a result of your disease or treatment?	1	2	3	4
40. Have you been feeling less feminine as a a result of your disease or treatment?	1	2	3	4
41. Did you find it difficult to look at yourself naked?	1	2	3	4
42. Have you been dissatisfied with your body?	1	2	3	4
43. Were you worried about your health in the future?	1	2	3	4

**During the past <u>four</u> weeks:**	**Not at All**	**A Little**	**Quite a Bit**	**Very Much**
44. To what extend were you interested in sex?	1	2	3	4
45. To what extend were you sexually active? (with our without intercourse)	1	2	3	4
46. Answer this only if you have been sexually active: To what extent was sex enjoyable for you?	1	2	3	4

<u>Please go on to the next page</u>

**During the past week:**		Not at All	A Little	Quite a Bit	Very Much
47.	Did you have any pain in your arm or shoulder?	1	2	3	4
48.	Did you have a swollen arm or hand?	1	2	3	4
49.	Was it difficult to raise your arm or to move it sideways?	1	2	3	4
50.	Have you had any pain in the area of your affected breast?	1	2	3	4
51.	Was the area of your affected breast swollen?	1	2	3	4
52.	Was the area of your affected breast oversensitive?	1	2	3	4
53.	Have you had skin problems on or in the area of your affected breast (e.g., itchy, dry, flaky)?	1	2	3	4
54.	Did you have any stiffness in your shoulder?	1	2	3	4

© Copyright 1994 EORTC Study Group on Quality of Life. All rights reserved. Version 1.0

Additional Body Image Scale (BIS)

**During the past week:**	**Not at All**	**A Little**	**Quite a Bit**	**Very Much**
55. Have you been self conscious about your appearance?	1	2	3	4
56. Have you been <u>dissatisfied</u> with your appearance when dressed?	1	2	3	4
57. Have you been feeling less sexually attractive as a result of your disease or treatment?	1	2	3	4
58. Did you avoid people because of the way you felt about your appearance?	1	2	3	4
59. Have you been feeling the disease or treatment has left you body less whole?	1	2	3	4
60. Have you been <u>dissatisfied</u> with the appearance of your scar	1	2	3	4

We are interested in any changes to your breast that may have resulted from any of your breast cancer treatments

61. Has the appearance of the skin in the area of your affected breast changed?	1	2	3	4
62. Has the overall appearance of your breast changed, compared with the other side?	1	2	3	4
63. Has your affected breast become smaller?	1	2	3	4
64. Has your affected breast become harder/firmer to the touch?	1	2	3	4
65. Is the position of the nipple of your affected breast different from the other side?	1	2	3	4
66. Have you had a problem getting a bra to fit?	1	2	3	4

Hospital Anxiety and Depression Scale (HADS)

H A D Scale

Doctors are aware that emotions play an important part in most illnesses. If your doctor know about these feelings he will be able to help you more.
This questionnaire is designed to help your doctor to know how you feel. Read each item and place a firm tick in the box opposite the reply which comes closest to how you have been feeling in the past week.
Don't take too long over your replies: your immediate reaction to each item will probably be more accurate than a long thought-out response.

Tick only one box in each section

I feel tense or 'wound up'
- Most of the time
- A lot of the time
- Time to time, Occasionally
- Not at all

I still enjoy the things I used to enjoy:
- Definitely as much
- Not quite so much
- Only a little
- Hardly at all

I get a sort of frightened feeling as if something awful is about to happen:
- Very definitely and quite badly
- Yes, but not too badly
- A little, but it doesn't worry me
- Not at all

I can laugh and see the funny side of things:
- As much as I always could
- Not quite so much now
- Definitely not so much now
- Not at all

Worrying thoughts go through my mind:
- A great deal of the time
- A lot of the time
- From time to time but not too often
- Only occasionally

I feel cheerful:
- Not at all
- Not often
- Sometimes
- Most of the time

I can sit at ease and feel relaxed:
- Definitely
- Usually
- Not often
- Not at all

I feel as if I am slowed down:
- Nearly all the time
- Very often
- Sometimes
- Not at all

I get a sort of frightened feeling like 'butterflies' in the stomach:
- Not at all
- Occasionally
- Quite Often
- Very often

I have lost interest in my appearance:
- Definitely
- I don't take so much care as I should
- I may not take quite as much care
- I take just as much care as ever

I feel restless as if I have to be on the move:
- Very much indeed
- Quite a lot
- Not very much
- Not at all

I look forward with enjoyment to things:
- As much as ever I did
- Rather less than I used to
- Definitely less than I used to
- Hardly at all

I get sudden feelings of panic:
- Very often indeed
- Quite Often
- Not very often
- Not at all

I can enjoy a good book or radio or TV programme:
- Often
- Sometimes
- Not often
- Very seldom

APPENDIX 10

EVALUATION OF THE ECONOMIC CONSEQUENCES

1. Overview

A cost-effectiveness analysis will be undertaken to compare the alternative interventions in IMPORT LOW. Costs will be estimated from the perspective of the NHS, and health effects quantified in terms of quality adjusted life-years (QALY). A decision analytic model will be developed to provide a framework within which to incorporate data from IMPORT LOW and, as appropriate, other sources. The model will extrapolate from the trial to quantify the implications for long-term quality-adjusted survival and costs of the differential breast cancer recurrence rates.

2. Resource costs

The primary perspective for the cost analysis will be that of the NHS, although selected additional costs will also be estimated. Within the trial, the following resource use data will be collected prospectively in all patients: hospital resource use (days in hospital by unit, therapeutic interventions post-randomisation including full details of radiotherapy provided, management of adverse events); primary care resources (visits to and from a GP or nurse); drugs prescribed. These data will be collected using case record forms completed at patients' clinic visits and using case-note interrogation, and on the basis of patients' responses to questionnaires. The resource use will be valued using prices and unit costs available at the time of analysis. These are likely to include the use of routine data on drugs (BNF), NHS Reference costs (108) to value particular hospital procedures and in-patient care and specific costing studies to estimate the cost of radiotherapy. As far as possible, costs will be estimated separately for each centre involved in the trial.

3. Effectiveness

The measure of effectiveness will focus on health effects, which will be quantified in terms of QALYs. Health related quality of life will be expressed in terms of the EuroQol (EQ)-5D which will be administered at baseline, 6 months, 1 year, 2 years and 5 years. The EQ-5D is a generic measure of health status, where health is characterised on five dimensions (mobility, self care, ability to undertake usual activities, pain, anxiety/depression) (109). At each point of follow-up, women will be asked to indicate their health on each dimension using one of three levels: no problems, moderate problems and severe problems. Each response locates a woman into one of 245 mutually exclusive health states, each of which has previously been valued on the 0 (equivalent to dead) to one (equivalent to good health) 'utility' scale based on interviews with a sample of 3,395 members of the UK public (110).

4. Analysis

Mean costs and QALYs will be calculated in each arm of the trial. These will account for administrative censoring using appropriate methods (111). Standard errors around these mean values using methods which can overcome the likely skewness in the data, for example non-parametric bootstrapping. To relate mean costs to mean QALYs, standard

methods of incremental cost-effectiveness analysis will be used, including reporting incremental cost-effectiveness ratios as appropriate. To express the uncertainty in cost-effectiveness resulting from sampling uncertainty, cost-effectiveness acceptability curves will be presented (112). These show the probability that each intervention is the more/most cost-effective given a range of threshold values that the NHS is willing to pay for an additional QALY. Multiple regression will be used, using cost-effectiveness as the dependent variable (113) to provide more precise estimates of the cost-effectiveness of each intervention, and assess cost-effectiveness according to pre-specified sub-group characteristics.

5. Extrapolation modelling

For decision-making about resource allocation in the management of breast cancer, mean costs and QALYs over patients' life expectancy should be estimated. Despite the relatively long-term follow-up in the trial, modelling methods are needed to extrapolate these data over a lifetime time horizon. For this purpose, a probabilistic decision model will be developed to relate within-trial local recurrence rates with long-term quality-adjusted survival and costs. These relationships will be based on available data at the point of trial analysis.

EQ - 5D

Health Questionnaire

By placing a tick (thus ☑) in one box in each group below, please indicate which statements best describe your own health state today.

Mobility
I have no problems in walking about ☐
I have some problems in walking about ☐
I am confined to bed ☐

Self-Care
I have no problems with self-care ☐
I have some problems washing or dressing myself ☐
I am unable to wash or dress myself ☐

Usual Activities *(e.g. work, study, housework, family or leisure activities)*
I have no problems with performing my usual activities ☐
I have some problems with performing my usual activities ☐
I am unable to perform my usual activities ☐

Pain/Discomfort
I have no pain or discomfort ☐
I have moderate pain or discomfort ☐
I have extreme pain or discomfort ☐

Anxiety/Depression
I am not anxious or depressed ☐
I am moderately anxious or depressed ☐
I am extremely anxious or depressed ☐

To help people say how good or bad a health state is, we have drawn a scale (rather like a thermometer) on which the best state you can imagine is marked 100 and the worst state you can imagine is marked 0.

We would like you to indicate on this scale how good or bad your own health is today, in your opinion. Please do this by drawing a line from the box below to whichever point on the scale indicates how good or bad your health state is today.

Your own health state today

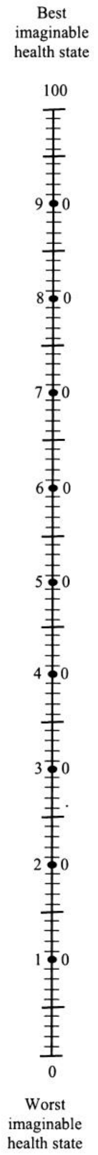

Additional Health Economics Questions

Over the past <u>six</u> weeks:

65. How many times have you been visited by your GP for any reason? (even if not related to your breast cancer).

66. How many times have you visited your GP for any reason? (even if not related to your breast cancer).

67. How many times have you been visited by a district nurse?

68. How many times have you been visited by a MacMillan nurse?

APPENDIX 11

RECOMMENDATIONS FOR RECURRENCE MAPPING

The primary endpoint for the IMPORT LOW trial is local tumour control in the ipsilateral breast. The highest quality data will be obtained by relating the 3-dimensional (3D) position of tumour recurrences/new primaries to the original radiotherapy plan.

The management of recurrences/new primaries varies from centre to centre. Also, it is important that the mapping techniques are practical to enable the maximum value from the information gathered. For this reason some suggested recurrence mapping recommendations have been devised with a variety of complexity. This takes into account that technology would be rapidly improving over the lifetime of the trial.

The patient information sheets and case report forms will be used to alert the patient and health care professionals of the IMPORT trial if a recurrence/new primary occurs.

Patients who proceed to Surgery without the involvement of the local Radiotherapy Centre

The surgeon indicates on a form with a simple schema the quadrant of the breast in which the recurrence/new primary occurs. The data are used to estimate where the recurrence/new primary is located with respect to the radiotherapy fields.

Patients who proceed to Surgery with the involvement of the local Radiotherapy Centre

It is expected that all patients would have a mammogram and ultrasound as part of standard diagnosis of a recurrence/new primary.

The recommendations are for optical, ultrasound and CT systems. Magnetic Resonance (MR) imaging is an alternative, with the advantage of being an accurate method of demonstrating tumour recurrence without additional radiation. The use of MRI, or other methods, for the recurrence mapping should be discussed with the imaging working party and QA team prior to use.

Simple mapping method using ultrasound and optical systems

- Patient is set-up in the radiotherapy simulator/treatment room in the original radiotherapy position.
- The light fields will be set-up to show position of the whole breast and partial breast radiotherapy fields.
- 2D ultrasound will record the centre, diameter and depth of the tumour and the position will be marked on the skin.
- If possible, the centre of gravity of the original tumour bed will be recorded from imaging the clips/seeds.

- It will be recorded whether the recurrence/new primary is inside the original partial breast volume, outside the partial breast volume, or in a borderline region (within 1 cm of the partial volume field edge).
- An estimate of the distance of the recurrence/new primary from the original tumour bed will be made if possible.

N.B. pre- and post-radiotherapy photographs of the patient's breast (including a linear scale) will give a score for the degree of breast shrinkage, and thus the level of accuracy of the above method.

Mapping using Computed Tomography

- Recurrences should be marked by means of a CT-visible coil or clip marker either at biopsy or once diagnosis has been confirmed. This should be discussed and arranged in co-operation with the breast imaging team.
- Patient will be set-up in the CT-simulator in the original radiotherapy position and CT-scanned.
- This CT scan will be co-registered with the original CT planning scan.
- The centre of gravity co-ordinates of the tumour recurrence (coil) and original tumour bed (clips/seeds) will be recorded.
- It will be recorded whether the tumour is inside the original partial breast volume, outside the partial breast volume, or in a borderline region (within 1 cm of the partial breast field edge).
- The distance of the tumour recurrence from the original tumour bed will be recorded.

N.B. pre- and post-radiotherapy photographs of the patient's breast, and change in CT breast contour, will indicate the degree of breast shrinkage, and thus the level of accuracy of the above method.

REFERENCES

1. EBCTCG. Favourable and unfavourable effects on long-term survival of radiotherapy for early breast cancer: an overview of the randomised trials. Early Breast Cancer Trialists Collaborative Group. Lancet 2000;355(9217):1757-70.
2. Clarke M., Collins R., Darby S., Davies C., Elphinstone P., Evans E., Godwin J., Gray R., Hicks C., James S., MacKinnon E., McGale P., McHugh T., Peto R., Taylor C., Wang Y. Effects of radiotherapy and of differences in the extent of surgery for early breast cancer on local recurrence and 15-year survival: an overview of the randomised trials. Lancet 2005;366(9503):2087-106.
3. Pierce S. M., Recht A., Lingos T. I., Abner A., Vicini F., Silver B., Herzog A., Harris J. R. Long-term radiation complications following conservative surgery (CS) and radiation therapy (RT) in patients with early stage breast cancer. Int J Radiat Oncol Biol Phys 1992;23(5):915-23.
4. Vrieling C., Collette L., Fourquet A., Hoogenraad W. J., Horiot J. C., Jager J. J., Pierart M., Poortmans P. M., Struikmans H., Van der Hulst M., Van der Schueren E., Bartelink H. The influence of the boost in breast-conserving therapy on cosmetic outcome in the EORTC "boost versus no boost" trial. EORTC Radiotherapy and Breast Cancer Cooperative Groups. European Organization for Research and Treatment of Cancer. Int J Radiat Oncol Biol Phys 1999;45(3):677-85.
5. Donovan E. M., Bleackley N. J., Evans P. M., Reise S. F., Yarnold J. R. Dose-position and dose-volume histogram analysis of standard wedged and intensity modulated treatments in breast radiotherapy. Br J Radiol 2002;75(900):967-73.
6. ICRU. Prescribing, recording and reporting photon beam therapy. Bethsada: Interantional Commission on Radiation Units and Measurements (ICRU); 1999.
7. ICRU. Prescribing, Recording and Reporting Photon Beam Therapy. Bethsada: International Commission on Radiation Units and Measurements (ICRU); 1993.
8. Veronesi U., Luini A., Del Vecchio M., Greco M., Galimberti V., Merson M., Rilke F., Sacchini V., Saccozzi R., Savio T., et al. Radiotherapy after breast-preserving surgery in women with localized cancer of the breast. N Engl J Med 1993;328(22):1587-91.
9. Forrest A. P., Stewart H. J., Everington D., Prescott R. J., McArdle C. S., Harnett A. N., Smith D. C., George W. D. Randomised controlled trial of conservation therapy for breast cancer: 6-year analysis of the Scottish trial. Scottish Cancer Trials Breast Group. Lancet 1996;348(9029):708-13.
10. Clark R. M., Whelan T., Levine M., Roberts R., Willan A., McCulloch P., Lipa M., Wilkinson R. H., Mahoney L. J. Randomized clinical trial of breast irradiation following lumpectomy and axillary dissection for node-negative breast cancer: an update. Ontario Clinical Oncology Group. J Natl Cancer Inst 1996;88(22):1659-64.
11. Fisher B., Anderson S., Fisher E. R., Redmond C., Wickerham D. L., Wolmark N., Mamounas E. P., Deutsch M., Margolese R. Significance of ipsilateral breast tumour recurrence after lumpectomy. Lancet 1991;338(8763):327-31.
12. Liljegren G., Holmberg L., Bergh J., Lindgren A., Tabar L., Nordgren H., Adami H. O. 10-Year results after sector resection with or without postoperative radiotherapy for stage I breast cancer: a randomized trial. J Clin Oncol 1999;17(8):2326-33.
13. Fisher B., Anderson S., Bryant J., Margolese R. G., Deutsch M., Fisher E. R., Jeong J. H., Wolmark N. Twenty-year follow-up of a randomized trial comparing total mastectomy, lumpectomy, and lumpectomy plus irradiation for the treatment of invasive breast cancer. N Engl J Med 2002;347(16):1233-41.
14. Fisher B., Bryant J., Dignam J. J., Wickerham D. L., Mamounas E. P., Fisher E. R., Margolese R. G., Nesbitt L., Paik S., Pisansky T. M., Wolmark N. Tamoxifen, radiation therapy, or both for prevention of ipsilateral breast tumor recurrence after lumpectomy in women with invasive breast cancers of one centimeter or less. J Clin Oncol 2002;20(20):4141-9.
15. Locker A. P., Ellis I. O., Morgan D. A., Elston C. W., Mitchell A., Blamey R. W. Factors influencing local recurrence after excision and radiotherapy for primary breast cancer. Br J Surg 1989;76(9):890-4.
16. Veronesi U., Maisonneuve P., Costa A., Sacchini V., Maltoni C., Robertson C., Rotmensz N., Boyle P. Prevention of breast cancer with tamoxifen: preliminary findings from the Italian randomised trial among hysterectomised women. Italian Tamoxifen Prevention Study. Lancet 1998;352(9122):93-7.
17. Veronesi U., Volterrani F., Luini A., Saccozzi R., Del Vecchio M., Zucali R., Galimberti V., Rasponi A., Di Re E., Squicciarini P., et al. Quadrantectomy versus lumpectomy for small size breast cancer. Eur J Cancer 1990;26(6):671-3.
18. Schnitt S. J., Abner A., Gelman R., Connolly J. L., Recht A., Duda R. B., Eberlein T. J., Mayzel K., Silver B., Harris J. R. The relationship between microscopic margins of resection and the risk of local

recurrence in patients with breast cancer treated with breast-conserving surgery and radiation therapy. Cancer 1994;74(6):1746-51.
19. Veronesi U., Salvadori B., Luini A., Greco M., Saccozzi R., del Vecchio M., Mariani L., Zurrida S., Rilke F. Breast conservation is a safe method in patients with small cancer of the breast. Long-term results of three randomised trials on 1,973 patients. Eur J Cancer 1995;31A(10):1574-9.
20. Veronesi U., Marubini E., Del Vecchio M., Manzari A., Andreola S., Greco M., Luini A., Merson M., Saccozzi R., Rilke F., et al. Local recurrences and distant metastases after conservative breast cancer treatments: partly independent events. J Natl Cancer Inst 1995;87(1):19-27.
21. Bartelink H., Horiot J. C., Poortmans P., Struikmans H., Van den Bogaert W., Barillot I., Fourquet A., Borger J., Jager J., Hoogenraad W., Collette L., Pierart M. Recurrence rates after treatment of breast cancer with standard radiotherapy with or without additional radiation. N Engl J Med 2001;345(19):1378-87.
22. Fisher B., Dignam J., Tan-Chiu E., Anderson S., Fisher E. R., Wittliff J. L., Wolmark N. Prognosis and treatment of patients with breast tumors of one centimeter or less and negative axillary lymph nodes. J Natl Cancer Inst 2001;93(2):112-20.
23. Holland R., Veling S. H., Mravunac M., Hendriks J. H. Histologic multifocality of Tis, T1-2 breast carcinomas. Implications for clinical trials of breast-conserving surgery. Cancer 1985;56(5):979-90.
24. Moffat D. F., Going J. J. Three dimensional anatomy of complete duct systems in human breast: pathological and developmental implications. J Clin Pathol 1996;49(1):48-52.
25. Welch H. G., Black W. C. Using autopsy series to estimate the disease "reservoir" for ductal carcinoma in situ of the breast: how much more breast cancer can we find? Ann Intern Med 1997;127(11):1023-8.
26. Salvadori B., Marubini E., Miceli R., Conti A. R., Cusumano F., Andreola S., Zucali R., Veronesi U. Reoperation for locally recurrent breast cancer in patients previously treated with conservative surgery. Br J Surg 1999;86(1):84-7.
27. Smith T. E., Lee D., Turner B. C., Carter D., Haffty B. G. True recurrence vs. new primary ipsilateral breast tumor relapse: an analysis of clinical and pathologic differences and their implications in natural history, prognoses, and therapeutic management. Int J Radiat Oncol Biol Phys 2000;48(5):1281-9.
28. Withers H. R., Taylor J. M., Maciejewski B. Treatment volume and tissue tolerance. Int J Radiat Oncol Biol Phys 1988;14(4):751-9.
29. Suwinski R., Withers H. R. Time factor and treatment strategies in subclinical disease. Int J Radiat Biol 2003;79(7):495-502.
30. Withers H. R., Peters L. J., Taylor J. M. Dose-response relationship for radiation therapy of subclinical disease. Int J Radiat Oncol Biol Phys 1995;31(2):353-9.
31. Yarnold J., Ashton A., Bliss J., Homewood J., Harper C., Hanson J., Haviland J., Bentzen S., Owen R. Fractionation sensitivity and dose response of late adverse effects in the breast after radiotherapy for early breast cancer: long-term results of a randomised trial. Radiother Oncol 2005;75(1):9-17.
32. Turesson I., Thames H. D. Repair capacity and kinetics of human skin during fractionated radiotherapy: erythema, desquamation, and telangiectasia after 3 and 5 year's follow-up. Radiother Oncol 1989;15(2):169-88.
33. Yarnold J., Donovan E. M., Bleackley N. J., Reise S., Regan J., Denholm E., Patel S., Ross G., Tait D., Evans P. Randomised trial of standard 2D radiotherapy versus 3D intensity modulated radiotherapy in patients prescribed breast radiotherapy (Oral). In. Radiotherapy and Oncology; 2002. p. 64, S15.
34. NICE. Improving Outcomes in Breast Cancer: National Institute for Clinical Excellence; 2002.
35. Webb S. Intensity Modulated Radiation Therapy. Bristol: Institute of Physics Publishing; 2000.
36. Herman M. G., Balter J. M., Jaffray D. A., McGee K. P., Munro P., Shalev S., Van Herk M., Wong J. W. Clinical use of electronic portal imaging: report of AAPM Radiation Therapy Committee Task Group 58. Med Phys 2001;28(5):712-37.
37. Antonuk L. E. Electronic portal imaging devices: a review and historical perspective of contemporary technologies and research. Phys Med Biol 2002;47(6):R31-65.
38. The Royal College of Radiologists The Society and The College of Radiographers, Institute of Physics and Engineering in Medicine. Development and Implementation of Conformal Radiotherapy in the United Kingdom: The Royal College of Radiologists; 2002.
39. START-Standardisation of Breast Radiotherapy, trial protocol; 1998.
40. Hoole A.C.F., Twyman N., Langmack K.A., Hebbard M., Lowrie D. Laser Scanning of Patient Outlines for Three-Dimensional Radiotherapy Treatment Planning. Physiological Measurement 2001(22):605-610.
41. Wilks R. J. An optical system for measuring surface shapes for radiotherapy planning. Br J Radiol 1993;66(784):351-9.

42. Liljegren G., Lindgren A., Bergh J., Nordgren H., Tabar L., Holmberg L. Risk factors for local recurrence after conservative treatment in stage I breast cancer. Definition of a subgroup not requiring radiotherapy. Ann Oncol 1997;8(3):235-41.
43. Wilks R. J., Childs P. J., Donovan E. M. Comparison of a lung fitting algorithm with CT data for tangential fields in radiotherapy of the breast. Br J Radiol 2004;77(917):414-9.
44. Vincent D., Beckham W., Delaney G. An assessment of the number of CT slices necessary to plan breast radiotherapy. Radiother Oncol 1999;52(2):179-83.
45. Wilks R. J., Bliss P. The use of a compensator library to reduce dose inhomogeneity in tangential radiotherapy of the breast. Radiother Oncol 2002;62(2):147-57.
46. Kestin L. L., Sharpe M. B., Frazier R. C., Vicini F. A., Yan D., Matter R. C., Martinez A. A., Wong J. W. Intensity modulation to improve dose uniformity with tangential breast radiotherapy: initial clinical experience. Int J Radiat Oncol Biol Phys 2000;48(5):1559-68.
47. Zackrisson B., Arevarn M., Karlsson M. Optimized MLC-beam arrangements for tangential breast irradiation. Radiother Oncol 2000;54(3):209-12.
48. Evans P. M., Hansen V. N., Mayles W. P., Swindell W., Torr M., Yarnold J. R. Design of compensators for breast radiotherapy using electronic portal imaging. Radiother Oncol 1995;37(1):43-54.
49. Fisher B., Anderson S., Redmond C. K., Wolmark N., Wickerham D. L., Cronin W. M. Reanalysis and results after 12 years of follow-up in a randomized clinical trial comparing total mastectomy with lumpectomy with or without irradiation in the treatment of breast cancer. N Engl J Med 1995;333(22):1456-61.
50. Veronesi U., Marubini E., Mariani L., Galimberti V., Luini A., Veronesi P., Salvadori B., Zucali R. Radiotherapy after breast-conserving surgery in small breast carcinoma: long-term results of a randomized trial. Ann Oncol 2001;12(7):997-1003.
51. Fentiman I. S., Poole C., Tong D., Winter P. J., Gregory W. M., Mayles H. M., Turner P., Chaudary M. A., Rubens R. D. Inadequacy of iridium implant as sole radiation treatment for operable breast cancer. Eur J Cancer 1996;32A(4):608-11.
52. Vicini F. A., Chen P. Y., Fraile M., Gustafson G. S., Edmundson G. K., Jaffray D. A., Benitez P., Pettinga J., Madrazo B., Ingold J. A., Goldstein N. S., Matter R. C., Martinez A. A. Low-dose-rate brachytherapy as the sole radiation modality in the management of patients with early-stage breast cancer treated with breast-conserving therapy: preliminary results of a pilot trial. Int J Radiat Oncol Biol Phys 1997;38(2):301-10.
53. Baglan K. L., Martinez A. A., Frazier R. C., Kini V. R., Kestin L. L., Chen P. Y., Edmundson G., Mele E., Jaffray D., Vicini F. A. The use of high-dose-rate brachytherapy alone after lumpectomy in patients with early-stage breast cancer treated with breast-conserving therapy. Int J Radiat Oncol Biol Phys 2001;50(4):1003-11.
54. King T. A., Bolton J. S., Kuske R. R., Fuhrman G. M., Scroggins T. G., Jiang X. Z. Long-term results of wide-field brachytherapy as the sole method of radiation therapy after segmental mastectomy for T(is,1,2) breast cancer. Am J Surg 2000;180(4):299-304.
55. Arthur D. W., Koo D., Zwicker R. D., Tong S., Bear H. D., Kaplan B. J., Kavanagh B. D., Warwicke L. A., Holdford D., Amir C., Archer K. J., Schmidt-Ullrich R. K. Partial breast brachytherapy after lumpectomy: low-dose-rate and high-dose-rate experience. Int J Radiat Oncol Biol Phys 2003;56(3):681-9.
56. Wazer D. E., Berle L., Graham R., Chung M., Rothschild J., Graves T., Cady B., Ulin K., Ruthazer R., DiPetrillo T. A. Preliminary results of a phase I/II study of HDR brachytherapy alone for T1/T2 breast cancer. Int J Radiat Oncol Biol Phys 2002;53(4):889-97.
57. Polgar C., Sulyok Z., Fodor J., Orosz Z., Major T., Takacsi-Nagy Z., Mangel L. C., Somogyi A., Kasler M., Nemeth G. Sole brachytherapy of the tumor bed after conservative surgery for T1 breast cancer: five-year results of a phase I-II study and initial findings of a randomized phase III trial. J Surg Oncol 2002;80(3):121-8; discussion 129.
58. Perera F., Engel J., Holliday R., Scott L., Girotti M., Girvan D., Chisela F., Venkatesan V. Local resection and brachytherapy confined to the lumpectomy site for early breast cancer: a pilot study. J Surg Oncol 1997;65(4):263-7; discussion 267-8.
59. Padhani A. R., Yarnold J. R., Regan J., Husband J. E. Magnetic resonance imaging of induration in the irradiated breast. Radiother Oncol 2002;64(2):157-62.
60. Keisch M., Vicini F., Kuske R. R., Hebert M., White J., Quiet C., Arthur D., Scroggins T., Streeter O. Initial clinical experience with the MammoSite breast brachytherapy applicator in women with early-stage breast cancer treated with breast-conserving therapy. Int J Radiat Oncol Biol Phys 2003;55(2):289-93.

61. Vaidya J.S., Tobias J.S., Houghton J., Joseph D., Wenz F., Hilaris B.S., et al. Intra-operative breast radiation: the targeted intra-operative radiotherapy (Targit) trial. Breast Cancer Research and Treatment 2003;82(1):S2.
62. Veronesi U., Orecchia R., Luini A., Gatti G., Intra M., Zurrida S., Ivaldi G., Tosi G., Ciocca M., Tosoni A., De Lucia F. A preliminary report of intraoperative radiotherapy (IORT) in limited-stage breast cancers that are conservatively treated. Eur J Cancer 2001;37(17):2178-83.
63. Vaidya J. S., Baum M., Tobias J. S., D'Souza D. P., Naidu S. V., Morgan S., Metaxas M., Harte K. J., Sliski A. P., Thomson E. Targeted intra-operative radiotherapy (Targit): an innovative method of treatment for early breast cancer. Ann Oncol 2001;12(8):1075-80.
64. Ribeiro G. G., Magee B., Swindell R., Harris M., Banerjee S. S. The Christie Hospital breast conservation trial: an update at 8 years from inception. Clin Oncol (R Coll Radiol) 1993;5(5):278-83.
65. Baglan K. L., Sharpe M. B., Jaffray D., Frazier R. C., Fayad J., Kestin L. L., Remouchamps V., Martinez A. A., Wong J., Vicini F. A. Accelerated partial breast irradiation using 3D conformal radiation therapy (3D-CRT). Int J Radiat Oncol Biol Phys 2003;55(2):302-11.
66. Fisher E. R., Sass R., Fisher B., Gregorio R., Brown R., Wickerham L. Pathologic findings from the National Surgical Adjuvant Breast Project (protocol 6). II. Relation of local breast recurrence to multicentricity. Cancer 1986;57(9):1717-24.
67. Vicini F. A., Remouchamps V., Wallace M., Sharpe M., Fayad J., Tyburski L., Letts N., Kestin L., Edmundson G., Pettinga J., Goldstein N. S., Wong J. Ongoing clinical experience utilizing 3D conformal external beam radiotherapy to deliver partial-breast irradiation in patients with early-stage breast cancer treated with breast-conserving therapy. Int J Radiat Oncol Biol Phys 2003;57(5):1247-53.
68. van Tienhoven G., Lanson J. H., Crabeels D., Heukelom S., Mijnheer B. J. Accuracy in tangential breast treatment set-up: a portal imaging study. Radiother Oncol 1991;22(4):317-22.
69. Lirette A., Pouliot J., Aubin M., Larochelle M. The role of electronic portal imaging in tangential breast irradiation: a prospective study. Radiother Oncol 1995;37(3):241-5.
70. Fein D. A., Fowble B. L., Hanlon A. L., Hoffman J. P., Sigurdson E. R., Eisenberg B. L. Does the placement of surgical clips within the excision cavity influence local control for patients treated with breast-conserving surgery and irradiation. Int J Radiat Oncol Biol Phys 1996;34(5):1009-17.
71. Hector C. L., Webb S., Evans P. M. The dosimetric consequences of inter-fractional patient movement on conventional and intensity-modulated breast radiotherapy treatments. Radiother Oncol 2000;54(1):57-64.
72. Valdagni R., Italia C. Early breast cancer irradiation after conservative surgery: quality control by portal localization films. Radiother Oncol 1991;22(4):311-3.
73. Hector C. L., Evans P. M., Webb S. The dosimetric consequences of inter-fractional patient movement on three classes of intensity-modulated delivery techniques in breast radiotherapy. Radiother Oncol 2001;59(3):281-91.
74. Nalder C. A., Bidmead A. M., Mubata C. D., Tait D., Beardmore C. Influence of a vac-fix immobilization device on the accuracy of patient positioning during routine breast radiotherapy. Br J Radiol 2001;74(879):249-54.
75. Yu C. X., Jaffray D. A., Wong J. W. The effects of intra-fraction organ motion on the delivery of dynamic intensity modulation. Phys Med Biol 1998;43(1):91-104.
76. Remouchamps V. M., Vicini F. A., Sharpe M. B., Kestin L. L., Martinez A. A., Wong J. W. Significant reductions in heart and lung doses using deep inspiration breath hold with active breathing control and intensity-modulated radiation therapy for patients treated with locoregional breast irradiation. Int J Radiat Oncol Biol Phys 2003;55(2):392-406.
77. Denham J. W., Sillar R. W., Clarke D. Boost dosage to the excision site following conservative surgery for breast cancer: it's easy to miss! Clin Oncol (R Coll Radiol) 1991;3(5):257-61.
78. Bedwinek J. Breast conserving surgery and irradiation: the importance of demarcating the excision cavity with surgical clips. Int J Radiat Oncol Biol Phys 1993;26(4):675-9.
79. Machtay M., Lanciano R., Hoffman J., Hanks G. E. Inaccuracies in using the lumpectomy scar for planning electron boosts in primary breast carcinoma. Int J Radiat Oncol Biol Phys 1994;30(1):43-8.
80. Harrington K. J., Harrison M., Bayle P., Evans K., Dunn P. A., Lambert H. E., Saidan Z., Lynn J., Stewart J. S. Surgical clips in planning the electron boost in breast cancer: a qualitative and quantitative evaluation. Int J Radiat Oncol Biol Phys 1996;34(3):579-84.
81. Hunter M. A., McFall T. A., Hehr K. A. Breast-conserving surgery for primary breast cancer: necessity for surgical clips to define the tumor bed for radiation planning. Radiology 1996;200(1):281-2.
82. Krawczyk J. J., Engel B. The importance of surgical clips for adequate tangential beam planning in breast conserving surgery and irradiation. Int J Radiat Oncol Biol Phys 1999;43(2):347-50.
83. Kovner F., Agay R., Merimsky O., Stadler J., Klausner J., Inbar M. Clips and scar as the guidelines for breast radiation boost after lumpectomy. Eur J Surg Oncol 1999;25(5):483-6.

84. Regine W. F., Ayyangar K. M., Komarnicky L. T., Bhandare N., Mansfield C. M. Computer-CT planning of the electron boost in definitive breast irradiation. Int J Radiat Oncol Biol Phys 1991;20(1):121-5.
85. Florell K., Halvorsen P. An accurate method for localization of the boost volume in breast radiotherapy. Med Dosim 1997;22(4):283-91.
86. DeBiose D. A., Horwitz E. M., Martinez A. A., Edmundson G. K., Chen P. Y., Gustafson G. S., Madrazo B., Wimbish K., Mele E., Vicini F. A. The use of ultrasonography in the localization of the lumpectomy cavity for interstitial brachytherapy of the breast. Int J Radiat Oncol Biol Phys 1997;38(4):755-9.
87. Haba Y., Britton P., Sinnatamby R., Moody A. , Sycamore C., Wilson C. Can ultrasound improve the accuracy of delivery of electron boost treatment following breast conserving surgery? Eur J Cancer 2001;37(37):38.
88. Leonard C., Harlow C. L., Coffin C., Drose J., Norton L., Kinzie J. Use of ultrasound to guide radiation boost planning following lumpectomy for carcinoma of the breast. Int J Radiat Oncol Biol Phys 1993;27(5):1193-7.
89. Smitt M. C., Birdwell R. L., Goffinet D. R. Breast electron boost planning: comparison of CT and US. Radiology 2001;219(1):203-6.
90. Vicini F. A., Jaffray D. A., Horwitz E. M., Edmundson G. K., DeBiose D. A., Kini V. R., Martinez A. A. Implementation of 3D-virtual brachytherapy in the management of breast cancer: a description of a new method of interstitial brachytherapy. Int J Radiat Oncol Biol Phys 1998;40(3):629-35.
91. Whipp E.C., Hartley-Davies R., Carroll J., Devrell C., Wells T., McKenzie A., Appleby H., Goddard P., Wakeley C., Halliwell M. The inaccuracies of conventional breast radiotherapy planning defined by MR imaging. In. ASCO; 2003. p. 25.
92. Horiot J. C., van der Schueren E., Johansson K. A., Bernier J., Bartelink H. The programme of quality assurance of the EORTC radiotherapy group. A historical overview. Radiother Oncol 1993;29(2):81-4.
93. Bolla M., Bartelink H., Garavaglia G., Gonzalez D., Horiot J. C., Johansson K. A., van Tienhoven G., Vantongelen K., van Glabbeke M. EORTC guidelines for writing protocols for clinical trials of radiotherapy. Radiother Oncol 1995;36(1):1-8.
94. SMAC. Quality Assurance in Radiotherapy: Standing Committee on Cancer of the Standing Medical Advisory Committee; 1991.
95. Aird E. G., Williams C., Mott G. T., Dische S., Saunders M. I. Quality assurance in the CHART clinical trial. Radiother Oncol 1995;36(3):235-44.
96. Venables K., Winfield E., Deighton A., Aird E., Hoskin P. A survey of radiotherapy quality control practice in the United Kingdom for the START trial. Radiother Oncol 2001;60(3):311-8.
97. IPEM. 81 Physics aspects of Quality Control in Radiotherapy. York: IPEM; 1999.
98. Hansen V. N., Evans P. M., Budgell G. J., Mott J. H., Williams P. C., Brugmans M. J., Wittkamper F. W., Mijnheer B. J., Brown K. Quality assurance of the dose delivered by small radiation segments. Phys Med Biol 1998;43(9):2665-75.
99. Aaronson N. K., Ahmedzai S., Bergman B., Bullinger M., Cull A., Duez N. J., Filiberti A., Flechtner H., Fleishman S. B., de Haes J. C., et al. The European Organization for Research and Treatment of Cancer QLQ-C30: a quality-of-life instrument for use in international clinical trials in oncology. J Natl Cancer Inst 1993;85(5):365-76.
100. Sprangers M. A , et al. The European Organisation for Research and Treatment of Cancer Breast Cancer-Specific Quality-of Life Questionnaire Module: First results from a three-country field study. J Clin Oncol 2001;5(Classic Papers and Current Comments):917-929.
101. Hopwood P., Fletcher I., Lee A., Al Ghazal S. A body image scale for use with cancer patients. Eur J Cancer 2001;37(2):189-97.
102. Zigmond A. S., Snaith R. P. The hospital anxiety and depression scale. Acta Psychiatr Scand 1983;67(6):361-70.
103. Cox D.R., et al. Quality of Life Assessment: Can we keep it simple? JRSSA 1992;155:353-392.
104. Agresti A. A survey of models for repeated ordered categorical response data. Stat Med 1989;8(10):1209-24.
105. McCullagh P., Nelder J.A. Generalised Linear Models. London: Chapman & Hall; 1989.
106. Liang K.Y., Zeger S.L. Longitudinal Data Analysis using Generalised Linear Models. Biometrika 1986;73:13-22.
107. Ibbotson T., Maguire P., Selby P., Priestman T., Wallace L. Screening for anxiety and depression in cancer patients: the effects of disease and treatment. Eur J Cancer 1994;30A(1):37-40.
108. The New NHS - 2002 Reference Cost. In. London: NHS Executive; 2002.

109. Sculpher M.J. The role and estimation of productivity costs in economic evaluation. In: Drummond MF, McGuire AE, editors. Theory and Practice of Economic Evaluation in Health. Oxford: Oxford University Press; 2001.
110. Kind P. The EuroQoL instrument: an index of health-related quality of life. In: Spilker B, editor. Quality of Life and Pharmacoeconomics in Clinical Trials. 2nd ed. Philadelphia: Lippincott-Raven; 1996.
111. Dolan P., Gudex C., Kind P., Williams A. A Social Tariff for EuroQol: Results from a UK General Population Survey. In: Centre for Health Economics Discussion Paper. University of York: CHE; 1995.
112. Lin D. Y., Feuer E. J., Etzioni R., Wax Y. Estimating medical costs from incomplete follow-up data. Biometrics 1997;53(2):419-34.
113. Hoch J. S., Briggs A. H., Willan A. R. Something old, something new, something borrowed, something blue: a framework for the marriage of health econometrics and cost-effectiveness analysis. Health Econ 2002;11(5):415-30.

<u>Appendix 3 – Risk stratification of local relapses according to the pathological characteristics at diagnosis</u>

Risk Stratification*	Clinical Characteristics	Patients with local relapse n=18	Total patients within risk group in trial population
**<u>Very low risk:</u>** **G1-2, ER+ve, HER2-ve, N-ve, LVSI absent**	G1-2, ER+ve, HER2-ve, N-ve, LVSI absent	10	1088
**<u>Higher risk including 1 or more of the following:</u>** **G3, ER poor, HER2+ve, N+ve, LVSI present**	G1-2, ER+ve, HER2+ve, N-ve, LVSI absent	1	30
	G1-2, ER poor, HER2-ve, N+ve, LVSI absent	1	1
	G1-2, ER poor, HER2-ve, N-ve, LVSI absent	1	20
	G3 ER+ve, HER2-ve, N-ve, LVSI absent	1	68
	G3 ER+ve, HER2+ve, N-ve, LVSI absent	1	10
	G3 ER poor, HER2-ve, N-ve, LVSI absent	3	33

*Arbitrary stratification into low and higher risk features. NB. Patients with grade 3 tumours only became eligible for the trial following a protocol amendment to eligibility criteria in March 2008

Appendix 4 - Summary of lung cancer laterality in relation to treated breast

Patient	Treatment group	Age at lung cancer diagnosis	Relationship to treated breast	Histology	Time from randomisation to development of lung cancer (months)
1	Whole	57	Ipsilateral	SCLC	39
2	Whole	76	Ipsilateral	Adenocarcinoma	35
3	Whole	87	Ipsilateral	Adenocarcinoma	28
4	Whole	60	Ipsilateral	Adenocarcinoma	55
5	Whole	66	Ipsilateral	SCLC	9
6	Whole	66	Ipsilateral	SCLC	64
7	Whole	70	Contralateral	ND	35
8	Whole	82	Contralateral	ND	6
9	Whole	61	Contralateral	ND	4
10	Whole	64	Contralateral	NSCLC	30
11	Whole	68	ND	Squamous	26
12	Reduced	67	Ipsilateral	Squamous	23
13	Reduced	62	Contralateral	Adenocarcinoma	20
14	Reduced	72	Contralateral	ND	40
15	Reduced	61	Contralateral	ND	1
16	Partial	73	Ipsilateral	Squamous	40
17	Partial	69	Contralateral	SCLC	44
18	Partial	64	Contralateral	ND	55
19	Partial	61	ND	Adenocarcinoma	53*

*note interval calculated using date of death as date of new primary unknown, ND=not documented

NB. Data on smoking status was not collected in the trial

Appendix 5 -Summary of cardiac deaths in relation to treated breast

Patient	Treatment group	Age at randomisation	Breast treated	Cause of cardiac death	Time from randomisation to cardiac death (months)
1	Whole	81	Right	Cardiac failure	28
2	Whole	80	Left	Ischaemic heart disease	14
3	Whole	85	Right	Ischaemic heart disease	29
4	Whole	80	Left	Hypertensive heart disease	35
5	Whole	76	Right	Acute coronary syndrome	30
6	Reduced	72	Left	Cardiac failure	36
7	Reduced	62	Right	Ischaemic heart disease	7
8	Partial	77	Right	Ischaemic heart disease	6
9	Partial	70	Left	Arrhythmia	16

NB. Data was not collected on smoking or cardiac history

Appendix 6 - Incidence of other severe late adverse effects by treatment group

	**Whole N=674**	**Reduced N=673**	**Partial N=669**
**Symptomatic rib fracture**			
**Reported**	3 (0.5%)	6 (0.9%)	1 (0.1%)
**Confirmed**	0	4 (0.6%)	0
**Symptomatic lung fibrosis**			
**Reported**	4 (0.6%)	5 (0.7%)	4 (0.6%)
**Confirmed**	3[1] (0.5%)	3 (0.5%)	2[2]
**Ischaemic heart disease**			
**Reported**	5 (0.7%)	3 (0.5%)	4 (0.6%)
**Confirmed**	3[3] (0.5%)	0	2 (0.3%)

[1] One patient with lung fibrosis confirmed but not related to radiotherapy
[2] One patient confirmed lung fibrosis but not related to radiotherapy
[3] Two cases of IHD confirmed as not related to radiotherapy due to medical history

Appendix 7 – The IMPORT LOW centres

Current principal investigators, past principal investigators, main co-investigators, surgeons and trials teams (including physicists, radiographers, nurses and data managers). Number of patients recruited in brackets; current PI in bold; past PI/PIs in bold italics.

* IMPORT trial management group member

Addenbrooke's Hospital, (89), **Dr C Coles***, Dr C Wilson, J Benson, P Forouhi, G Wishart, N Twyman*, J Wilkinson, V Joslin, C Spain, B Stratton; Aintree Hospital, (1), **Dr P Robson**, L Beresford, L Pauls; Basildon Hospital, (2), **Dr C Trask**, A Salih, H Taylor, K Maitland; Beatson West of Scotland Cancer Centre, (120), **Dr A Alhasso***, Dr P Canney, Dr G Fraser, Dr D Ritchie, Dr M Rizwanullah, C Wilson, L Romics, I Reid, M McKirdy, K Krupa, E Eltahir, J Ferguson, K Ogston, R Ferguson, A McIntyre*, J Dixon, J Fleming; Bedford Hospital, (4), **Dr S Smith**, L Kelly, K Newton, D Pearson, A Willis; Belfast City Hospital, (8), **Dr J Clarke**, S McIntosh, S McCarron, J Cousins, S Hynds, S Murray, O Stewart, G Totten; Bradford Royal Infirmary, (8),**Dr E Thomas**, Dr C Bradley, Dr A Raine, L Bamford, A Kay, L Kenyon, H Robertshaw, E Waldron; Bristol Haematology and Oncology Centre, (45), **Dr C Comins**, ***Dr A Bahl***, Dr M Beresford, J Cook, Z Rayter, Z Winters, H Appleby, S Cowley, D Griffiths, L Pope, M Simmonds, D Weston, S Yarrow, E Harris, E Hiscott, H Saldana; Burnley General Hospital, (17), **Dr M Hogg**, Dr W Appel, Dr P Desai, J Iddon, D Sandilands, S Brothwood, H Clements, S Ainsworth, S Ashworth, K Riley, V Tickle; Charing Cross Hospital, (33), **Dr S Cleator**, ***Dr C Lowdell***, Dr R Ahmad, Dr P Riddle, Dr C Lewanski, A Corder, J Donnelley, R McLauchlan, S Magwaro, A Thoi, C Welgemoed, Y Fatola; Cheltenham General Hospital, (36), **Dr P Jenkins**, Dr K Benstead, Dr J Bowen, Dr R Counsell, Dr S Elyan, Dr R Owen, C Chan*, A Sammon, C Fowler, N Bulmer, B Milliner, S Webster, J Chittock, J Forkes, K Trigg-Hogarth, S Wronski; Christie Hospital, (66), **Dr J Loncaster**, Dr J Coote, Dr B Magee, Dr V Misra, Dr A Stewart, Dr R Welch, N Bundred, W Julian, S March, C Burke, E Molloy, A Walker; City Hospital, Birmingham, (3), **Dr D Spooner**, J Dasgin, B Gammon; Clatterbridge Cancer Centre/Warrington and Halton Hospitals, (73), **Dr R Sripadam/Dr S Tolan**, ***Dr I Syndikus****, Dr P Schofield, G Copeland, J Lund, J Kirk*, H Mayles, A Baker, G Herbert, M Horton, R Madew, L Lee, C Lowthian; Colchester General Hospital, (18), **Dr M Mukesh**, ***Dr P Murray***, Dr D Basu, M Porter, J Cheyne, L Dewar, C Driscoll, N Barke, B Elvin, J Taylor, L Thorogood; County Hospital, Stafford, (3), **Dr A Jegganathen**, ***Dr C Brammer, Dr A Al-Niaimi***, Dr L Pettit, S Newman, C Harvey, C Parton, D Sirdefield, J Stacey; Derriford Hospital, Plymouth, (3), **Dr U Panwar**, ***Dr S Kelly***, N Blackler, C Meyerhoff, H Congdon, J Pascoe, H Smith; Guy's and St Thomas' Hospital, (114), **Dr Elinor Sawyer***, ***Dr J Dobbs***, Dr S Ahmad, Dr L Brazil, Dr S Harris, Dr A Swampillai, Prof A Tutt*, N Beechey-Newman, P Fentiman, A Kothari, C Thomas, L Bower, D Nunnery, C Williams, S Donaghey, G Kuenzig; Hereford County Hospital, (24), **Dr J Scaife**, ***Dr S Guglani*** , A Corder, J Donnelley, J Birch, S Boyd, G Horsfield, Z Roberts; Ipswich Hospital, (26), **Dr E Sherwin**, ***Dr J Le Vay***, Dr V Ramachandran, T Archer, C Mortimer H James*, A Poynter, C MacKenzie, Y Tricker, N Lloyd, A Riley, P Taylor-Neale; James Paget Hospital, (32), **Dr A Harnett***, Dr S Alexander, S Downey, J Pereira, A Brooks, J Harman, C Whitehouse; Kettering General Hospital, (25), **Mr S Musa**, ***Dr R Matthew***, Dr S Reza, S Ahmad, A Datta, S Khan, M Rashed, R Stewart, A Guerdette, J Walsh; Kidderminster Hospital, (28), **Dr M Churn**, Dr P Ramachandra, S Davis, S Stringer, H Tranter; Leeds Cancer centre/St James Institute of Oncology, (44), **Dr S Kumar**, Dr J Adlard, Prof D Dodwell, Dr F Roberts, R Achuthan, K Horgan, M Lansdown, P Turton, S Allen, S Hartup, A Henson, R Thorpe; Leicester Royal Infirmary, (4), **Dr L Aznar-Garcia**, ***Dr I Boiangui***, Dr C Esler, Dr A Gore, Dr K Sampson, A Stotter, S Dzumhur, S Wright, J Potterton, A King, L Nelson; Lincoln County Hospital, (15), **Dr A Chaudhuri**, Dr A Papakostidi, Dr T Sreenivaran, Dr D Threkinniin, Dr N Vaithilingam, R Nair, M El-Sheemy, I Copson, L Walsh, O Francis, C Lockwood, A Sloan; Macclesfield District General Hospital, (40), **Dr L Barraclough**, ***Dr Pooja Jain***, Dr R Hunter, J Kokan, D MacKenzie, C Roshanlall, S Entwhistle, C Gaskell, L Hardstaff, P Hill, M McCurrie, B Townley; Milton Keynes University Hospital, (16), **Dr H Eldeeb**, ***Dr J Stewart***, Dr K Chin, Dr A Taylor, R Fletcher, C Ford, R Streeton, H Thomas, V Webb; Mount Vernon Hospital, (73), **Dr P Ostler**, Dr A Makris, Dr M Ah-See, Dr M Harrison, Dr N Shah, M Pittam, D Ravichandran, S Raymond, J Wood, C Anderson, J Bowes, J Dixon, J Evans, M Masters, E

Windmill; New Cross Hospital, (35), **Dr M Khan**, ***Dr M Churn***, Dr P Ramachandra, Dr C Brammer, Dr R Allerton, B Isgar, P Matey, H Howard, R Goldfinch, N Begum, V Carter, A Grant, R Pawarwoo, E Sharman; Norfolk and Norwich University Hospital, (108), **Dr A Harnett***, Dr A Bulman, Dr L Senarath, Dr H Stubbings, S Barber*, A Williams, A Cooper, A Hoosenbux, E Malone, K Oosterom, S Walker; North Manchester General Hospital, (15), **Dr J Loncaster**, Dr B Magee, J Howat, J Walls, T Hodgkiss, J Johnson; Northampton General Hospital, (38), **Dr R Agarwal**, ***Dr J Stewart, Dr H Eldeeb, Dr C MacMillan***, Dr R Matthew, Dr L Houghton, P Evans, N Wallbank, E Weaver, L Evans, S Jose, V Mason; Peterborough City Hospital, (27), **Dr C Jephcott**, Dr J Wrigley, E Duggan, J Rezulski, G Selenica, L Thurston, E Edwards, M Hartley, M Mulligan; Pinderfields General Hospital, (22), **Dr S Kumar**, Dr R Mekki, Dr F Roberts, J Ball, S Buckley, T Lowry; Queen Elizabeth Hospital, Birmingham, (45), **Dr S Anwar**, Dr D Spooner, Dr A Stevens, M Tiffany, V Harrop, E Southgate, R Stephens, S Chetiyawardana, R Spence; Queen Elizabeth Hospital, King's Lynn, (13), **Dr M Daly**, Dr A Ahmad, Dr S Aslam, Dr I Haidar, Dr G Horan, C Rankin, S Shedwell, N Ward, H Webb; Royal Albert Edward Infirmary, (7), **Dr C Anandadas**, ***Dr N Bayman***, L Perchard, J Bradshaw, L Devereaux, A Power; Royal Blackburn Hospital, (9), **Dr M Hogg**, L Jones, A Podder, R Watson, K Beard, J Hargreaves, K Jewers; Royal Bolton Hospital, (8), **Dr R Welch**, S Cocks, E Curran, L Dawson, K Jewers, Z Gall; Royal Cornwall Hospital, (136), **Dr D Wheatley***, Dr A Gould, Dr A Thomson, I Brown, R Laney, D Beech, L Johns, T King, M Kirkpatrick; Royal Liverpool University Hospital, (17), **Dr N Thorp**, C Holcombe, S Poonawala, A Tansley, A Waghorn, N Stephenson, M Horton, N Higgins, B Raynor; Royal Oldham Hospital, (2), **Dr V Misra**, W Cook, J Johnson; Royal Preston Hospital, (12), **Dr M Hogg**, Dr E Young, Dr W Appel, Dr A Birtle, Dr G Skailes, Dr E Young, G Shentall, E Fillingham, A Smith, S Brothwood, S Cornthwaite, C Robinson, H Spickett; Royal Shrewsbury Hospital, (76), **Dr S Khanduri**, ***Dr R Agrawal****, P Evans, N Wallbank, E Weaver, M Adams, L Evans, S Jose, S Pope; Royal Stoke University Hospital, (125), **Prof M Brunt***, Dr A Al-Niaimi, Dr D Gahir, J Adjogatse, T Duffy, R Kirby, K Thorley, R Smith, K Parkinson, J Storer; Royal Sussex County Hospital, (1), **Dr D Bloomfield**, Dr R Simcock, E O'Malley, A Yelland, C Zammit, L Alexander, J Tremlett, J Greatbatch; Royal United Hospital, Bath, (12), **Dr M Beresford**, ***Dr H Newman***, Dr S Mancero, Dr S Masson, E Matthews, S Whittle, S MacGregor, S Curtis, T Tylee; Russells Hall Hospital, Dudley, (13), **Dr R Allerton**, ***Dr P Ramachandra***, Dr G Georgiev, K Kanyi, K McGarry; Southend University Hospital, (81), **Dr H Algurafi**, ***Dr A Robinson***, E Gray, N Rothnie, C Tsokadayi, L Romero, J Nippard, L Googe, A McPherson, E Mphansi; Southport and Formby District General Hospital, (9), **Dr K Hayat**, K Gardner, J Griffiths, A Morris, L Schinkel; St George's Hospital, Tooting, (2), **Dr A Kirby***, ***Dr I Locke***, L Gothard; St Mary's Hospital, Paddington, (3), **Dr S Cleator**, Dr S Ball, Dr O Hatcher, M Allnutt; The Royal Marsden Hospital, Surrey, (116), **Dr A Kirby***, ***Prof J Yarnold****, Dr M Dryzmala, Dr I Locke, Dr D Tait, A Agrawal, A Alabassi, W Allum, G Gui, F MacNeill, P Pilarisetti, C Pogson, N Roche, H Convery, E Donovan*, S Eagle, L Gothard, C Lucy, S Martin, G Sharp; University College Hospital, London, (5), **Dr M McCormack**, Dr G Blackman, Dr A Cassoni, Dr M Gaze, Dr R Stein, P Davies, L Allington, J Heywood, S Moinuddin, T Patel, S Wickers; Velindre Cancer Centre, Cardiff, (30), **Dr P Barrett-Lee**, Dr A Borley, Dr N Iqbal, S Slade, C Haymann, L Penketh, A McQueen, A Weaver; West Suffolk Hospital, (12), **Dr M Moody**, Dr C Woodward, Dr I Goumaris, Dr A Ahmad, G Brett, D Clements-Dimmock, F Flynn, S Hale; Weston General Hospital, (12), **Dr T Wells**, ***Dr M Tomlinson***, Dr H Berry, Dr C Graham, Dr D Radstone, D Coles, H Lloyd-Jones, V Pixton; Whiston Hospital, (16), **Dr R Sripadam**, Dr Z Malik, H Donaghy, M Harvey, N Hornby, M Robinson; Worcester Royal Hospital, (32), **Dr M Churn**, ***Dr J Bowen***, Dr R Counsell, Dr K Benstead, K Ledger, S Anderson, J Tyler; Worthing Hospital, (9), **Dr D Bloomfield**, ***Dr S Westwell***, Dr R Simcock, Dr A Moss, Dr S Castell, Dr S Mitra, Dr A Moss, Dr A Ring, S House, R Gomez, S Moore.

The following centres also opened: Blackpool Victoria Hospital; Ealing Hospital; Eastbourne Hospital; Hinchingbrooke Hospital; Leighton Hospital, Crewe; Queen's Hospital, Romford; Torbay General Hospital; Chorley and South Ribble Hospital; Tameside General Hospital

Appendix 8 – The IMPORT Trial Management Group

Current membership

Rajiv Agrawal
Abdulla Alhasso
Wail Al Sarakbi
Sarah Barber
Gillian Barnett
Judith Bliss
Murray Brunt
Charlie Chan
Charlotte Coles
Ellen Donovan
David Eaton
Ian Ellis
Philip Evans
Clare Griffin
Adrian Harnett
Emma Harris
Jo Haviland
Penny Hopwood
Hayley James
Monica Jefford
Anna Kirby
Cliona Kirwan
Julie Kirk
Anne McIntyre
Andrew Poynter
Elena Provenzano
Christine Rawlings
Elinor Sawyer
Mark Sculpher
Mark Sydenham
Isabel Syndikus
Jenny Titley
Yatman Tsang
Andrew Tutt
Nicola Twyman
Karen Venables
Duncan Wheatley
Maggie Wilcox
Gordon Wishart
John Yarnold

Past members

Peter Bliss
Laura Ciurlionis
John Dewar
Stephen Ebbs
John Le Vay (deceased March 2008)
Helen Mayles
Judith Mills
Judith Robinson
Georges Sumo
Anna Winship
John Winstanley
